# Signalling inhibition by ponatinib disrupts productive alternative lengthening of telomeres (ALT)

**DOI:** 10.1038/s41467-023-37633-3

**Published:** 2023-04-06

**Authors:** Frances Karla Kusuma, Aishvaryaa Prabhu, Galen Tieo, Syed Moiz Ahmed, Pushkar Dakle, Wai Khang Yong, Elina Pathak, Vikas Madan, Yan Yi Jiang, Wai Leong Tam, Dennis Kappei, Peter Dröge, H. Phillip Koeffler, Maya Jeitany

**Affiliations:** 1 School of Biological Sciences, Nanyang Technological University, Singapore, Singapore; 2 Cancer Science Institute of Singapore, National University of Singapore, Singapore, Singapore; 3 Department of Biochemistry, Yong Loo Lin School of Medicine, National University of Singapore, Singapore, Singapore; 4 Genome Institute of Singapore, Agency for Science, Technology and Research (A*STAR), Singapore, Singapore; 5 NUS Center for Cancer Research, Yong Loo Lin School of Medicine, National University of Singapore, Singapore, Singapore; 6 Cedars-Sinai Medical Center, Division of Hematology/Oncology, UCLA School of Medicine, Los Angeles, CA USA; 7 Department of Hematology-Oncology, National University Cancer Institute of Singapore (NCIS), National University Hospital, Singapore, Singapore; 8 Present Address: Hefei Institutes of Physical Science, Chinese Academy of Sciences, Hefei, P. R. China

**Keywords:** Cancer therapy, Cell signalling, DNA damage and repair

## Abstract

Alternative lengthening of telomeres (ALT) supports telomere maintenance in 10–15% of cancers, thus representing a compelling target for therapy. By performing anti-cancer compound library screen on isogenic cell lines and using extrachromosomal telomeric C-circles, as a bona fide marker of ALT activity, we identify a receptor tyrosine kinase inhibitor ponatinib that deregulates ALT mechanisms, induces telomeric dysfunction, reduced ALT-associated telomere synthesis, and targets, in vivo, ALT-positive cells. Using RNA-sequencing and quantitative phosphoproteomic analyses, combined with C-circle level assessment, we find an ABL1-JNK-JUN signalling circuit to be inhibited by ponatinib and to have a role in suppressing telomeric C-circles. Furthermore, transcriptome and interactome analyses suggest a role of JUN in DNA damage repair. These results are corroborated by synergistic drug interactions between ponatinib and either DNA synthesis or repair inhibitors, such as triciribine. Taken together, we describe here a signalling pathway impacting ALT which can be targeted by a clinically approved drug.

## Abstract (web summary)

The kinase inhibitor Ponatinib inhibits an ABL1-JUN signalling axis and alters telomere homeostasis, reduces telomere synthesis, and provokes telomere dysfunction in cancer cells which employ the alternative lengthening of telomeres mechanisms.

## Introduction

Telomeres are nucleoprotein structures at the ends of chromosomes that undergo gradual shortening in normal dividing somatic cells, eventually leading to replicative senescence^1,2^. Cancer cells can overcome this proliferative control system and achieve immortality by activating telomere elongation mechanisms. Most cancer cells re-express telomerase, a reverse transcriptase responsible for adding TTAGGG repeat sequences at human chromosome ends^3^. However, a subset of cancer cells relies on homology-directed repair (HDR) mechanisms termed Alternative Lengthening of Telomeres (ALT)^4,5^. ALT is detected in ~10–15% of all cancers, and is especially prevalent in several tumour types, such as osteosarcoma, soft tissue sarcoma and glioblastoma^6^.

Telomeres of ALT cells are prone to heightened levels of replicative stress and potential fork stalling and exhibit spontaneous nicks and breaks that can elicit activation of DNA damage repair (DDR) pathways^7^. Both collapsed replication forks and DNA breaks can prime for homology-directed repair at telomeres of ALT cells. HDR can engage several distinct pathways including RAD51-dependent Homologous Recombination (HR)^8,9^ or RAD51-independent break-induced replication (BIR)^10^.

Recent discoveries and understanding of the machineries implicated in ALT telomere homeostasis have uncovered potential therapeutical candidates in ALT-dependent cancers. For instance, ALT cancer cells are highly sensitive to ATR inhibition, a core regulator of DNA recombination^11^. Similarly, depletion of FANCM, a protein promoting resolution of stalled replication forks at ALT telomeres, results in increased levels of telomere dysfunction^12,13^. Co-depletion of FANCM and BLM or FANCM and BRCA1^13^, as well as disruption of the FANCM-BTR complex, selectively decreases ALT cell viability^12^. In addition to these strategies, other targets and drug compounds have been proposed, such as targeting TSPYL5^14^ or the chromatin assembly factor HIRA^15^, inhibiting a DNA damage-p53-AKT pathway^16^, inhibiting lysine acetyl transferases^17^, using the cisplatin derivative Tetra-Pt(bpy)^18^ or preventing ATM activity^19^. Despite the progress in identifying potentially targetable key molecular players in ALT, there is a lack of clinical management specific for patients with ALT cancers. Moreover, our understanding of signalling pathways critical for ALT and how these may be clinically exploited is limited.

To uncover therapeutic vulnerabilities that may be unique to ALT processes, we performed an anti-cancer compound library screen on IMR90-derived ALT- or telomerase-positive isogenic cell lines^20^. We identified a multi-receptor tyrosine kinase inhibitor (RTKI), ponatinib, which affects ALT activity. Ponatinib was initially designed to inhibit native and mutant forms of the chimeric kinase BCR-ABL^21,22^, but was also shown to have a broad inhibitory effect on multiple other kinases^22^.

We show that ponatinib elicited enhanced killing of ALT-positive cells, increased levels of extrachromosomal telomeric C-circles, mediated telomeric dysfunction and replicative stress in ALT cells, and specifically induced DNA damage in p53-deficient ALT cells. Importantly, ponatinib limited ALT-associated telomere synthesis. Mechanistically, we describe an ABL1-JNK-JUN signalling circuit which is implicated in ponatinib’s deregulation of ALT activity. Furthermore, we determine synergistic interactions between ponatinib and other anti-cancer drugs, such as DNA synthesis inhibitor, triciribine, and ATM inhibitor, KU-60019, and show that combining ponatinib and triciribine is highly effective in killing ALT cells.

Overall, our study uncovers a therapeutic avenue for ALT cancers and offers a repurposing opportunity of a clinically approved anti-cancer agent towards ALT cancer management.

## Results

### Ponatinib and PD173074 reduce ALT cell viability and increase levels of extrachromosomal telomeric C-circles

**Fig. 1:**
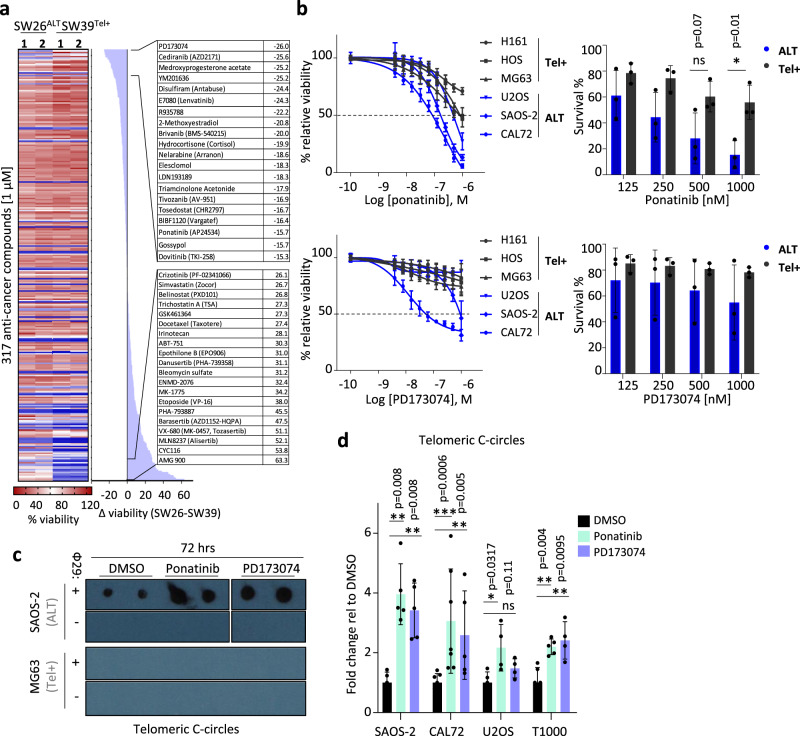
RTKIs ponatinib and PD173074 reduce ALT cell viability and increase levels of extrachromosomal telomeric C-circles. **a** Heatmap showing viability of SW26 (ALT) and SW39 (telomerase-positive) cells treated with 317 anti-cancer compounds (at 1 µM) ranked according to higher sensitivity in SW26 cells. The right panel shows difference of viability between SW26 and SW39 for the top hits. **b** Viability assays on telomerase-positive (Tel + ) or ALT sarcoma cell lines treated with increasing concentrations of ponatinib or PD173074 for 72 h. Values represent mean ± SD of percentage of absorbance relative to DMSO of at least three experiments each performed with three biological replicates (*n* = 9). The right graphs show the percentage of survival of the ALT and telomerase-positive cells in the independent experiments for selected concentrations (*N* = 3). (*P < 0.05, as determined by two-tailed *t* test). **c** Representative dot blot for telomeric C-circle assay showing levels of telomeric C-circles in SAOS-2 and MG63 cells treated for 72 h with either ponatinib (at 250 nM), PD173074 (at 500 nM) or DMSO as a control. Rolling circle amplification reactions with or without the φ29 polymerase are spotted on the membrane. **d** Quantification of telomeric C-circles in several ALT cell lines treated with either ponatinib or PD173074 for 72 h. Ponatinib was used at 250 nM for SAOS-2, U2OS and T1000, and at 125 nM for CAL72. PD173074 was used at 500 nM for SAOS-2 and U2OS, at 250 nM for T1000 and at 125 nM for CAL72. Each dot represents a biological replicate (SAOS-2: *n* = 5; CAL72: *n* = 7 for DMSO and ponatinib and *n* = 5 for PD173074; U2OS: *n* = 5 for DMSO and *n* = 4 for ponatinib and PD173074; T1000: *n* = 6 for DMSO, *n* = 5 for ponatinib and *n* = 4 for PD173074). Error bars represent ± SD. (*P < 0.05, **P < 0.01, ns not significant, as determined by two-tailed Mann–Whitney test). Source data are provided as a Source Data file.

To identify potential drug vulnerabilities in cells using ALT mechanisms, we performed a comparative anti-cancer compound library screen on two immortalized cell lines, both derived from the same parental cell line (IMR90): SW26 (ALT-positive) and SW39 (telomerase-positive)^20^ (Fig. 1a). This screen revealed a subset of drugs to which SW26 ALT cells showed increased sensitivity. Subsequent testing of shortlisted compounds (PD173074, cediranib, YM201636, nelarabine, tivozanib, ponatinib and dovitinib) in a panel of osteosarcoma (OS) cell lines and a pair of well-differentiated liposarcoma (LPS) cell lines validated two receptor tyrosine kinase inhibitors (RTKIs) (ponatinib, a pan-BCR-ABL inhibitor^22^ and PD173074, an inhibitor of FGF and VEGF receptors^23^) as compounds exhibiting enhanced killing of ALT cells (Fig. 1b and Supplementary Fig. 1a–d). Sensitivity to ponatinib of normal lung-derived IMR90 fibroblasts was comparable to that of telomerase-positive cells (Supplementary Fig. 1e), and increased sensitivity of ALT cells to ponatinib was confirmed in clonogenic assays (Supplementary Fig. 1f, g). ALT cell lines used in these experiments were ATRX-deficient (ref. ^11^ and Supplementary Fig. 1d). To assess whether the two RTKIs interfere with ALT mechanisms, we evaluated the drug effects on levels of extrachromosomal telomeric C-circles, an established specific marker of ALT activity^24^. Ponatinib induced an increase of telomeric C-circles after 72 h (Fig. 1c, d and Supplementary Fig. 2a) or 24 h (Supplementary Fig. 2b) of treatment in all tested ALT cell lines, but neither in telomerase-positive cells (Fig. 1c) nor in IMR90 normal fibroblasts (Supplementary Fig. 2d). PD173074 had similar effect to ponatinib in some but not all tested cell lines. The increase in C-circle generation was specific to these two RTKIs since treatment of ALT cells with other RTKIs or anti-cancer compounds identified in the drug screen (dovitinib, cediranib, R778 and YM201636) or hydroxyurea did not increase telomeric C-circles at the tested concentrations (Supplementary Fig. 2c). The effect of ponatinib on telomeric C-circles was further validated using a (CCCTAA)_4_-DIG labelled probe (Supplementary Fig. 2e) and shown to be dependent on the presence of APBs, since SAOS-2 cells lacking PML did not show aberrant levels of C-circles after ponatinib treatment (Supplementary Fig. 2f, g). In clonogenic assays, PML-deficient cells, while showing lower clonogenic potential overall, were more resistant to ponatinib treatment (Supplementary Fig. 2h). These results indicate that ponatinib’s effects on ALT cancer cells depend at least partly on the presence of APBs and the ALT activity.

### Ponatinib provokes telomeric dysfunction in ALT cells

**Fig. 2:**
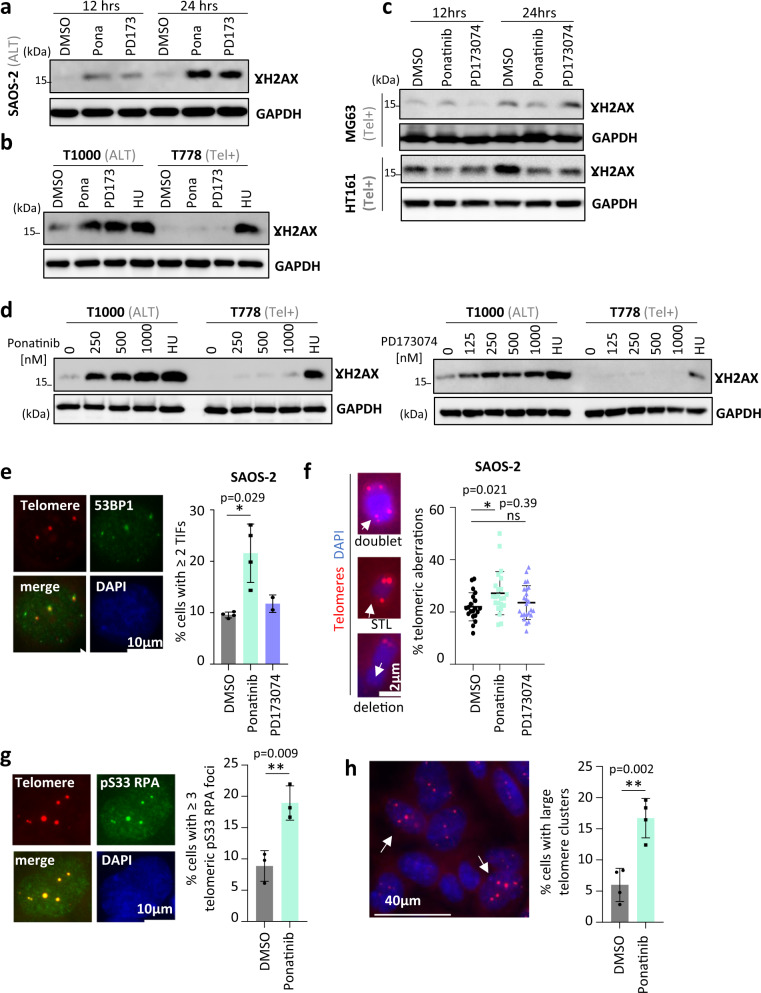
Ponatinib provokes telomeric dysfunction in ALT cells. **a**–**d** Western blots of γH2AX after treatment with ponatinib (250 nM) or PD173074 (500 nM) (**a**; **c**), or ponatinib (250 nM), PD173074 (250 nM) or hydroxyurea (HU; 2 mM) for 24 h (**b**). Findings in (**a**, **b**) were reproduced at least once more. (For (**c**), *n* = 1 for MG63 and *n* = 2 for HT161) **d** Cells were treated with increasing drug concentrations or hydroxyurea (HU; 2 mM) for 24 h (*n* = 1). (GAPDH = loading control). **e** Telomere dysfunction-induced foci (TIFs) detected by telomere (TelG-cy3) FISH staining and 53BP1 immunostaining in cells treated for 48 h with ponatinib (250 nM) or PD173074 (500 nM). The graph shows mean percentage of cells (± SD) with 2 or more TIFs from at least two biological replicates. (*P < 0.05, determined by two-tailed Mann–Whitney test). (Number of analysed cells = 511, 604 and 231 for DMSO, ponatinib, and PD173074, respectively). **f** Telomeric aberrations analysis by telomeric FISH staining on metaphases after 72 h of treatments. The left panel shows examples of aberrations: doublet telomeres, single telomere loss (STL) or telomere deletion. The graph shows percentage of telomeric aberrations per metaphase (mean ± SD). (*P < 0.05, ns = not significant, determined by two-tailed unpaired *t* test); (number of analysed chromosome extremities: DMSO (1727 from 20 metaphases), ponatinib (2279 from 23 metaphases) and PD173074 (2073 from 24 metaphases), from two independent experiments). **g** Detection of pS33 RPA (green) at telomeres (red) in SAOS-2 cells treated with ponatinib (250 nM) for 48 h. Mean (± SD) percentage of cells with three or more colocalizations events is depicted. (**P < 0.01, determined by two-tailed unpaired *t* test; analysed cell number = 413 (DMSO) and 407 (ponatinib), from three biological replicates of two independent experiments). **h** Quantification of cells with at least one large telomere cluster/focus (≥2 microns). Values are mean ± SD. (**P < 0.01, determined by two-tailed unpaired *t* test; total number of analysed cells = 552 (DMSO) and 587 (ponatinib), from four biological replicates of two independent experiments). The image shows examples of cells with large telomere foci (arrows). The lower right cell is shown in (**g**) with pS33 RPA staining. Source data are provided as a Source Data file.

While the origin of formation of extrachromosomal telomeric C-circles in ALT cells is not fully determined, studies indicated that they could be products of either telomere-based recombination activity^25,26^ or telomeric damage^27,28^. In ALT cells, telomere recombination activity is marked and promoted by ALT-associated PML bodies (APBs)^29,30^. Cells treated with either ponatinib or PD173074 did not show changes in the number of APBs (Supplementary Fig. 3a). We therefore hypothesized that the drug-induced accumulation of telomeric C-circles may result from damaged telomeres and assessed global levels of DNA damage using γH2AX as a marker. Both ponatinib and PD173074 caused high levels of DNA damage shown by western blot and immunostaining (Fig. 2a, b and Supplementary Fig. 3b), in ALT cells but not in telomerase-positive cells (Fig. 2b, c), even at high drug concentrations that kill both ALT and telomerase-positive cells in an equal manner (Fig. 2d). In ALT cell lines, induction of global DNA damage was seen in p53-deficient cell lines SAOS-2 (TP53^null^) and T1000 (TP53^WT^/MDM2^AMP^), but not in p53 WT U2OS ALT cells (Supplementary Fig. 3c). To evaluate whether these drugs caused specific telomere damage, we quantified the levels of telomere dysfunction-induced foci (TIFs)^31^. Here, only ponatinib increased the frequency of TIFs, as marked by 53BP1 and telomere staining in SAOS-2 and CAL72 ALT cells (Fig. 2e and Supplementary Fig. 3d) but neither in telomerase-positive HT161 and HOS cell lines (Supplementary Fig. 3e) nor in IMR90 fibroblasts (Supplementary Fig. 3f). DNA breaks created by ponatinib were also visualized by pulse-field gel electrophoresis of embedded cells (Supplementary Fig. 4a) and subsequent Southern blotting revealed the presence of telomeric DNA in genomic fragments ranging between 30 and 100 kbs (Supplementary Fig. 4b). Consistent with its effect in damaging telomeres, ponatinib increased the frequency of telomere aberrations visualized on metaphase spreads (Fig. 2f). Telomeric RNA molecules (TERRA) at several chromosome ends were also increased after treatment with either ponatinib or PD173074 (Supplementary Fig. 4c), another indication of potential dysfunction^32^ or replication stress at telomeres^33^. In addition, a significantly higher frequency of micronuclei (Supplementary Fig. 4d) and an increase of phosphorylated RPA (Replication protein A) at Serine 33 (pS33 RPA) (Supplementary Fig. 4e), were seen in cells treated with ponatinib, indicating ongoing genomic instability^34^ and forms of replicative stress^35–37^. Indeed, specific telomere-associated replicative stress, revealed by the colocalization of pS33 RPA and telomeric DNA, was increased in ponatinib-treated cells (Fig. 2g). Furthermore, ponatinib promoted the formation of large telomeric foci after 24 or 48 h of treatment (Fig. 2h and Supplementary Fig. 4f), potentially due to telomere aggregation following telomeric DNA damage and stalled replication forks^8,38^. In parallel, ponatinib’s-induced increase of telomeric C-circles did not result in telomere elongation, as shown by telomere restriction fragment (TRF) analysis (Supplementary Fig. 4g). Furthermore, ponatinib treatment triggered senescence in two of the three ALT cell lines but not in telomerase-positive cells (Supplementary Fig. 5a, b). The induction of senescence was independent of p53 since SAOS-2 is p53-deficient and transcript levels of p21 were not increased after ponatinib treatment in both SAOS-2 and CAL72 (p53 WT) cells (Supplementary Fig. 5c). Instead, an increase of other senescence-related effectors was seen after treatment with ponatinib, including the cell cycle inhibitor p27 and the stress-driven transcription factor ATF4^39–41^. The induction of p27 was further confirmed by Western blot in CAL72 cells (Supplementary Fig. 5d). Moreover, in CAL72 cells, an induction of Rb and a reduction of relative phosphorylation of Rb were detected by western blot after 72 h of treatment with ponatinib (Supplementary Fig. 5d). These results indicate that ponatinib may activate several distinct senescence pathways, including a p27-Rb axis in CAL72 cells and an ATF4- and/or p27-dependent pathway in SAOS-2 (Rb-deficient^42^) cells. Altogether, these findings suggest that treatment of ALT cells with ponatinib can result in DNA damage, dysfunctional telomeres, and induction of senescence.

### Ponatinib intercepts telomere synthesis in ALT cells

**Fig. 3:**
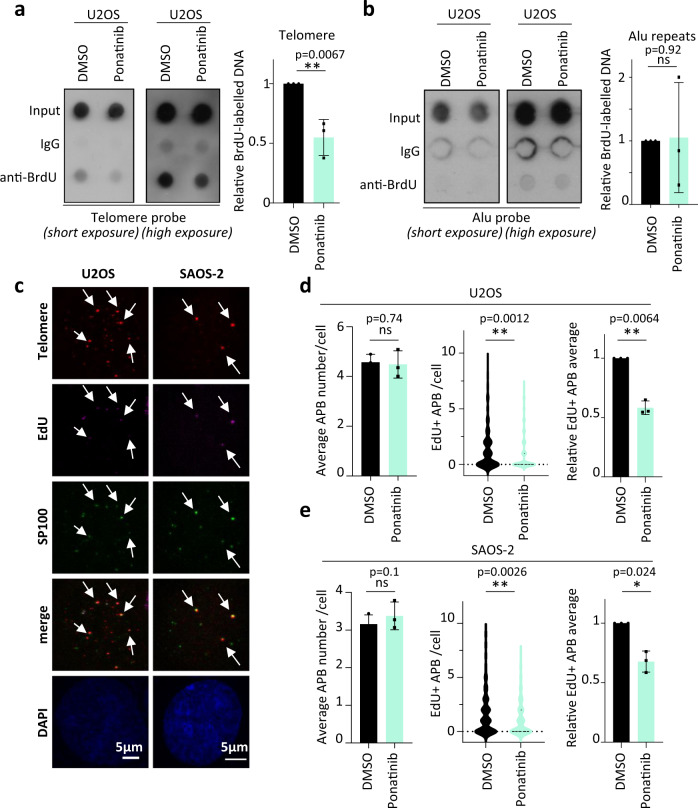
Ponatinib intercepts telomeric synthesis. **a**, **b** BrdU pull-down dot blots to detect newly synthesized telomeres (**a**) or Alu repeats (**b**). Graphs show mean ± SD of three experiments. (**P < 0.01, ns = not significant, as determined by two-tailed *t* test). **c** Representative images of ATSA (ALT telomere DNA synthesis in APB) assays in U2OS and SAOS-2 cells analysed in (**d**, **e**). ATSA was scored as a colocalization between telomeres (Red), EdU (purple) and SP100 (green) (highlighted with arrows). **d**, **e** Scoring of ATSA in APB-positive U2OS (**d**) and SAOS-2 (**e**) cells. The left panels show the average total number of APBs per cell, the middle graphs depict the distribution of EdU+ APBs per cell, while the right graphs show the relative average of EdU+ APBs per experiment. A total number of more than 200 cells per condition were scored from three independent experiments (*N* = 3, error bars represent ± SD). (**P < 0.01, *P < 0.05, ns = not significant, as determined by two-tailed paired or unpaired *t* tests). Source data are provided as a Source Data file.

Among the ALT osteosarcoma cell lines, U2OS had the lowest response to ponatinib treatment (Fig. 1b and Supplementary Fig. 5b), while still showing a moderate increase in telomeric C-circle levels after treatment (Fig. 1d). Having longer telomeres than the other tested ALT cells (Supplementary Fig. 4g), we reasoned that this cell line may be a suitable model to assess whether telomere synthesis is affected by ponatinib and to test whether the increase of C-circles is associated with de novo telomere synthesis. We performed a pulsed-BrdU incorporation assay followed by a telomeric dot blot (Fig. 3a). Ponatinib interfered with telomeric replication and synthesis evidenced by a decrease of newly BrdU-labelled telomeres (Fig. 3a), while it had no noticeable effect on Alu repetitive elements (Fig. 3b). These events were not due to cell cycle differences, as 24 h of ponatinib treatment did not affect overall BrdU incorporation (Supplementary Fig. 6a) nor did it alter cell cycle distribution of neither ALT nor telomerase-positive cells (Supplementary Fig. 6b). Specific telomere synthesis in APBs, assessed by ATSA (ALT telomere DNA synthesis in APBs) assay^43^, was also significantly reduced in both U2OS and SAOS-2 cells after 18–20 h of treatment with ponatinib (Fig. 3c–e). These results corroborate that ponatinib-induced telomeric C-circle levels reflect an increase of telomere damage and replicative stress concomitant with reduced telomere synthesis.

### ALT cells are targeted by ponatinib in vivo

**Fig. 4:**
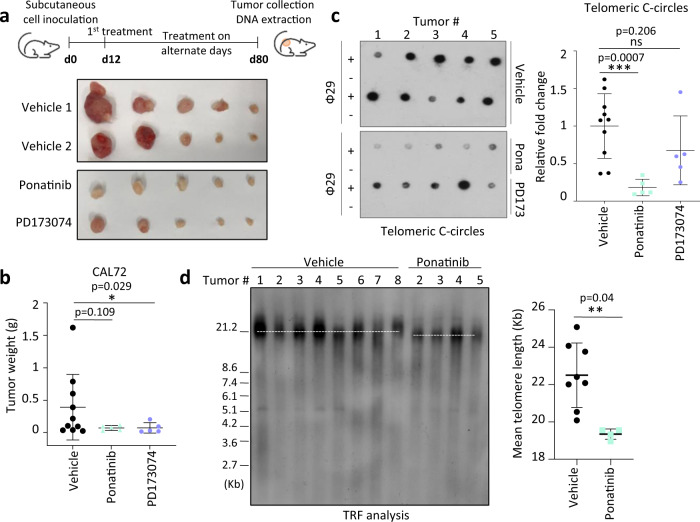
ALT cells are targeted by ponatinib in vivo. **a** Upper panel: experimental design for efficacy testing of ponatinib and PD173074 in subcutaneous CAL72 xenograft tumors. Lower panels: tumor pictures at the end of the experiment. **b** Tumor weight in mice treated with either vehicle (control) (*n* = 10), ponatinib (*n* = 5) or PD173074 (*n* = 5) (both 20 mg/kg). Error bars represent mean ± SD. (*P < 0.05, as determined by one-tailed Mann–Whitney test). **c** Telomeric C-circles in each tumor depicted in (**a**). Right graph represents relative fold change ± SD compared to the mean of vehicle group. (***P < 0.001, ns = not significant, as determined by two-tailed Mann–Whitney test; *n* = 10 for DMSO and 5 for ponatinib- and PD173074-treated groups). **d** TRF (Telomere Restriction Fragment) analysis of tumors treated with either vehicle (control) or ponatinib to determine average telomere length. Mean telomere length ± SD is shown in the right panel. (**P < 0.01, as determined by two-tailed Mann–Whitney test; *n* = 8 for DMSO and *n* = 4 for ponatinib). Source data are provided as a Source Data file.

To evaluate the effect of ponatinib on the ALT phenotype in vivo, CAL72 ALT osteosarcoma cells were subcutaneously inoculated in immunodeficient mice. These cells were previously reported to form tumors in mice^44^. Mice were treated by oral gavage with either ponatinib, PD173074 or vehicle as a control (Fig. 4a). Both ponatinib and PD173074 reduced the tumor burden in mice (Fig. 4a, b) without affecting their body weight (Supplementary Fig. 7a). The levels of telomeric C-circles were then measured in each tumor to assess the ALT potential. Residual tumors from mice treated with ponatinib, but not with PD173074, exhibited a marked reduction in the levels of their telomeric C-circles (Fig. 4c). By Telomere Restriction Fragment (TRF) analysis, we noticed that remaining tumors from mice treated with ponatinib had a slightly shorter average telomere length when compared to tumors from the control group (Fig. 4d and Supplementary Fig. 7b). These results potentially indicate that cells with higher ALT activity were particularly sensitive to ponatinib during initial treatments and were inhibited for in vivo growth or that the remaining tumors have a lower ALT potential. We conclude that ponatinib is potent at altering the telomeric homeostasis of ALT cells both in vitro and in vivo.

### Ponatinib affects several signalling pathways and alters transcription of genes associated with DNA replication and repair

**Fig. 5:**
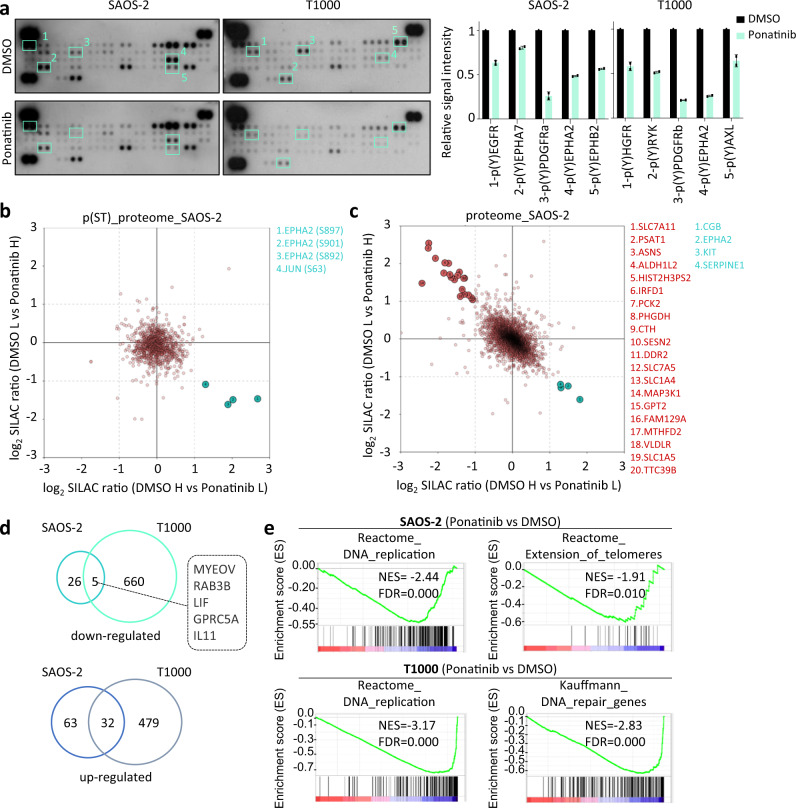
Ponatinib inhibits several signalling pathways and alters transcription of DNA repair genes. **a** Receptor tyrosine kinase (RTK) array using protein extracts from either SAOS-2 or T1000 cells treated with ponatinib (250 nM) for 6 h. Graphs show relative signal quantification of spots with differential intensity (marked in boxes and numbers) between DMSO and ponatinib conditions (*n* = 2 technical replicates for each spot, error bars are ± SD). **b**, **c** Two-dimensional SILAC ratio plots for quantitative phosphoproteomics (**b**) and proteomics (**c**) showing peptides with lower (in green) phosphorylation (**b**) or total expression levels (**c**) after 24 h of ponatinib treatment (lower right quadrants). Up-regulated proteins upon ponatinib treatment are annotated in red (upper left quadrant). **d** Venn diagrams of overlapping differentially expressed genes (down- or up-regulated) after ponatinib (250 nM) treatment of SAOS-2 and T1000 cells for 24 h, identified by RNA-sequencing analysis. **e** Gene set enrichment (GSE) plots showing broad reduction in expression of genes implicated in DNA replication or repair. NES normalized enrichment score, FDR false discovery rate. Source data are provided as a Source Data file.

To identify ponatinib’s mode of action on ALT telomeres, targets as well as cellular effects elicited by its treatment were identified. Ponatinib’s RTK inhibitory profile was determined using a phospho-RTK array in SAOS-2 and T1000 ALT cell extracts after 6 h of drug treatment (Fig. 5a). While tyrosine phosphorylation of multiple RTKs was reduced by ponatinib, only EPHA2 was the common hit between the two cell lines (Fig. 5a). Additional sustained inhibition of serine or threonine (S/T) signalling and global protein level changes were furthermore identified by SILAC-based quantitative phosphoproteome analysis (Fig. 5b and Supplementary Data 1) and proteomic analysis (Fig. 5c and Supplementary Data 2) in SAOS-2 cells treated with either DMSO or ponatinib for 24 h. Here, specific phosphopeptides of EPHA2 and JUN were found to be less abundant in ponatinib-treated cells (Fig. 5b). Total protein levels of EPHA2, as well as few other proteins were decreased, while levels of some other proteins implicated in various biological processes (e.g., amino acid biosynthesis or transport, metabolic pathways) were increased (Fig. 5c). Using western blot, we validated that phosphorylation of JUN at Serine 63 as well as total protein levels of JUN and EPHA2 were reduced upon ponatinib treatment (Supplementary Fig. 8a).

To delineate further cellular changes induced by ponatinib, we measured by RNA-sequencing gene expression changes after 24 h of drug treatment (Fig. 5d, e and Supplementary Data 3). The number of overlapping differentially expressed genes (DEGs) between SAOS-2 and T1000 ALT cells was low, with only 5 genes down-regulated by ponatinib in both cell lines (Fig. 5d). Interestingly, Gene Set Enrichment Analysis (GSEA) showed that ponatinib reduced the expression of gene sets related to either DNA repair or replication in both cell lines (Fig. 5e).

The 32 common up-regulated genes included those related to cellular response to stress and other metabolic processes (Supplementary Fig. 8b), including ATF4 and the endoplasmic reticulum stress-inducible protein FAM129A/Niban^45^. The up-regulation of both ATF4 and FAM129A was further confirmed by western blot (Supplementary Fig. 8c). These data confirm the earlier observation that cells treated with ponatinib activate a stress-response that may lead to senescence (Supplementary Fig. 5c).

### JUN and ABL1 suppress telomeric C-circles formation

**Fig. 6:**
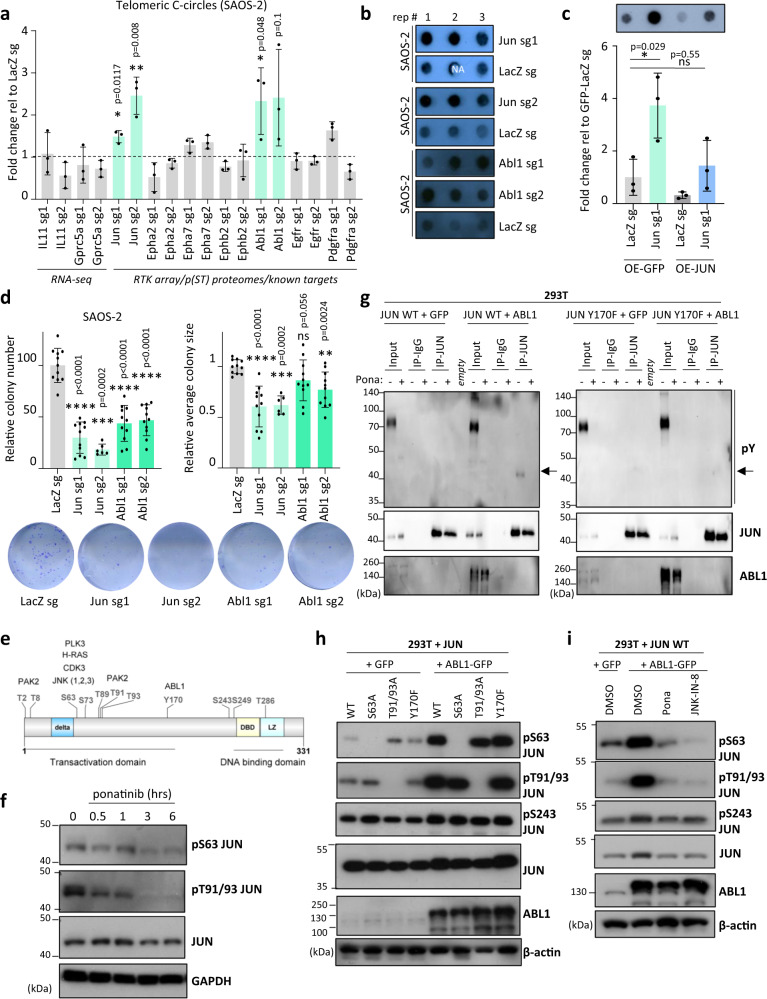
Ponatinib inhibits an ABL1-JNK-JUN signalling circuit. **a** Telomeric C-circle screen in SAOS-2 cells transduced with CRISPR-Cas9 and guide RNAs (sg1 and sg2) targeting individual candidates. Fold change is relative to corresponding control (LacZ sg cells). Graph shows mean ± SD from three biologically independent replicates (*n* = 3). (*P < 0.05, **P < 0.01, determined by two-tailed unpaired *t* test). **b** Telomeric C-circle dot blots from JUN or ABL1-depleted and corresponding LacZ sg SAOS-2 cells. **c** Telomeric C-circles in SAOS-2 cells either lacking endogenous, overexpressing (OE) JUN or both. C-circle levels were normalized to those in LacZ sg/OE-GFP cells. (*P < 0.05, ns = not significant, determined by two-tailed *t* test; *n* = 3; error bars represent ± SD). **d** Colony formation assays of JUN- or ABL1-depleted cells. Graphs represent relative colony number or size (average ± SD of three experiments (*N* = 3) (*n* = 6 or 11 biological replicates)). Lower panel shows representative images. (**P < 0.01, ***P < 0.001, ****P < 0.0001, ns=not significant, determined by two-tailed Mann–Whitney test). **e** Schematic of human JUN protein depicting selected phosphosites and kinases known to regulate these sites. (Created with DOG 2.0). **f** Western blot for either JUN or phosphorylated forms of JUN (at serine 63 (pS63) or threonines 91 and 93 (pT91/93) in SAOS-2 cells treated with 250 nM ponatinib. GAPDH = sample processing control. (*n* = 2). **g** Detection of tyrosine phosphorylated JUN by immunoprecipitating (IP) JUN followed by a western blot using anti-phospho-tyrosine (pY) antibody. The arrows show the expected band for phosphorylated JUN. 293 T cells were transfected with either Wild-type (WT) or phospho-mutant (Y170F) JUN, together with ABL1 or GFP as a control. Sixteen hours after transfection, cells were treated with ponatinib (250 nM) or DMSO for 6 h. Levels of JUN and ABL1 were evaluated in the immunoprecipitates and protein extracts (input). (*n* = 2) **h**, **i** Western blots for phosphorylated forms of JUN or total levels of JUN and ABL1 after overexpressing wild-type (WT) or phosphomutant forms of JUN (S63A, T91/93 A, Y170F) together with GFP or ABL1 in 293T cells for 20 h (**h**). In (**i**), cells were also treated for 6 h with either ponatinib (250 nM), JNK-IN-8 (500 nM), or DMSO as control. β-actin = sample processing control. (*n* = 1). Source data are provided as a Source Data file.

To test the effect of ponatinib’s targets on telomeres of ALT cells, telomeric C-circle levels were used as a functional read-out after silencing these targets. Known targets of the drug (e.g., ABL1) as well as candidates identified in our proteomic and transcriptomic analyses (Fig. 5) were selected. These were individually depleted in SAOS-2 cells using CRISPR-Cas9 system, followed by telomeric C-circle levels measurement. Depletion of JUN and ABL1 led to an increase in telomeric C-circles (Fig. 6a, b and Supplementary Fig. 9a, b), reminiscent of the increase induced by ponatinib. Similar effects of either ABL1 or JUN depletion on telomeric C-circles were seen in other but not all tested ALT cell lines (Supplementary Fig. 9c, d). The effect of JUN on telomeric C-circles was further confirmed by a rescue experiment with overexpression of gRNA-resistant JUN in SAOS-2 cells lacking endogenous JUN (Supplementary Fig. 9e). Restoring JUN in these cells reduced levels of C-circles (Fig. 6c), confirming a role of JUN in modulating levels of telomeric C-circles. In contrast, knocking-down of EPHA2 (kinase inhibited by ponatinib in both SAOS-2 and T1000 cells) did not affect levels of telomeric C-circles (Fig. 6a and Supplementary Fig. 9f), suggesting that EPHA2 is not involved in ponatinib’s action on telomeres. Importantly, cells lacking JUN and treated with ponatinib did not exhibit a further increase in levels of telomeric C-circles (Supplementary Fig. 9g, h). In addition, depletion of JUN increased telomere-induced dysfunctional foci in SAOS-2 cells (Supplementary Fig. 9i).

Moreover, depletion of either JUN or ABL1 reduced cell survival shown by a reduction of colony number and size in clonogenic assays in all tested ALT cell lines (Fig. 6d and Supplementary Fig. 10a, b). We also verified that ponatinib reduced JUN levels in the other ALT cells (Supplementary Fig. 10c). In parallel, JUN depletion was less deleterious on telomerase-positive HOS, HT161 and MG63 cells (Supplementary Fig. 10d, e). These results suggest that inhibition of JUN and ABL1 axis mediates ponatinib’s action on telomeres and survival of ALT cells.

### Ponatinib inhibits an ABL1-JNK-JUN signalling circuit

While ABL1 is known to be directly inhibited by ponatinib^22^, JUN is not a known direct target of the drug. JUN contains several phosphorylation sites, both in its DNA binding domain (e.g., Serine at position 243) and in its transactivation domain (e.g., Serine at position 63, Threonines at positions 91 and 93, and Tyrosine at position 170) (Fig. 6e). These phosphorylation events can alter the binding activity, as well as the stability of JUN. For instance, phosphorylation of JUN at C-terminal residues T231, S243 and S249 alters its DNA binding ability^46^, while phosphorylation at S63, S73, T91 and/or T93 potentiates its transcriptional activity and increases its stability^47–50^. We found that phosphorylation at S63 and T91/93, but not at S243 were reduced after ponatinib treatment (Fig. 6f and Supplementary Fig. 10f). Loss of phosphorylation at these sites occurred as early as 30 min after treatment with ponatinib, potentially impacting the stability of JUN and subsequently leading to its degradation by 24 h (Supplementary Figs. 8a and 10c).

Tyrosine phosphorylation of JUN at position 170 has been reported to be mediated by nuclear ABL1^51^. To test whether ABL1 phosphorylates JUN, JUN and ABL1 were overexpressed in 293T cells and JUN was immunoprecipitated to detect its tyrosine phosphorylation levels by western blot using an antibody against phosphotyrosines (pY) (Fig. 6g). JUN phosphorylation was substantially enhanced in the presence of ABL1, and lost when cells were treated with ponatinib, or when a phospho-mutant (JUN Y170F) was expressed (Fig. 6g). This result shows that JUN is regulated by an ABL1-dependent signalling pathway that is targetable by ponatinib and confirms that Y170 is the main tyrosine regulated by this signalling.

To test whether JUN phosphorylation at Y170 regulates its stability and/or phosphorylation at other phosphosites, we examined the phosphorylation levels of JUN at S63, T91/93 and S243 after expressing JUN either alone or with ABL1. In the presence of ABL1, JUN’s phosphorylation at S63 and T91/93 but not at S243 was enhanced, even in a mutant form of JUN (Y170F) that is not phosphorylated by ABL1 (Fig. 6h, i), indicating that ABL1 additionally regulates the phosphorylation of these residues indirectly. Indeed, these phosphorylation events were reduced upon treatment with ponatinib (Fig. 6i). S63 and T91/93 sites are phosphorylation substrates of several S/T kinases, including JNKs (JUN N-terminal Kinases)^47,49^. To test whether JNK kinases are implicated in the ABL1 signalling pathway regulating JUN, we used a selective pan-JNK inhibitor, JNK-IN-8^52^. Treating SAOS-2 cells with JNK-IN-8 for 6 h decreased phosphorylation of JUN at S63 (Supplementary Fig. 10g). Likewise, JNK-IN-8 inhibited phosphorylation at S63 and T91/93 induced by overexpression of ABL1 (Fig. 6i), showing that JNK kinases are intermediate effectors in the ABL-JUN signalling axis. JNKs inhibition using JNK-IN-8 alone, was not sufficient to induce an increase in telomeric C-circles (Supplementary Fig. 10h), indicating that ponatinib’s effects on ALT telomeres is likely to involve additional pathways.

### JUN regulation of ALT activity in SAOS-2 cells may be independent of its transcriptional function

JUN is a central member of the AP-1 transcriptional family^53^ and has been described as a transcription activator of several genes. To pinpoint JUN’s potential transcriptional targets in SAOS-2 cells, RNA-sequencing was performed to compare cells either lacking or overexpressing JUN and their corresponding control cells (Supplementary Fig. 11a–e). We identified two sub-clusters of RNAs that are either up-regulated in the absence of JUN and down-regulated after JUN overexpression (sub-cluster 1; Supplementary Fig. 11b) or that show the inverse expression pattern (sub-cluster 2; Supplementary Fig. 11d). Common genes between these sub-clusters and differentially expressed genes (DEGs) after ponatinib treatment in SAOS-2 cells were determined (Supplementary Fig. 11c, e). Only three overlapping genes were identified between sub-cluster 1 and ponatinib-induced differentially expressed genes (DEGs) (Supplementary Fig. 11c), while seven transcripts were commonly reduced either in the absence of JUN or after ponatinib treatment (Supplementary Fig. 11e). Among these genes, only IL11, which does not alter telomeric C-circle levels (Fig. 6a), was also found down-regulated in ponatinib-treated T1000 cells (Fig. 5d), indicating that JUN’s effect on telomeric C-circle levels is unlikely to involve its transcriptional activity.

**Fig. 7:**
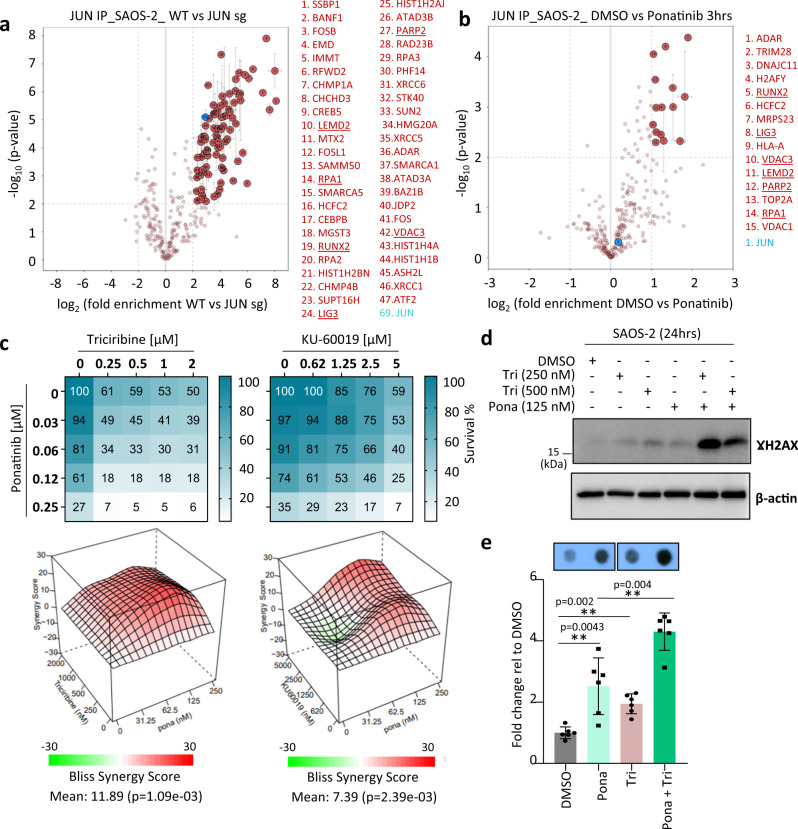
Ponatinib can synergize with DNA repair or synthesis inhibitors. **a**, **b** Volcano plots showing JUN interactome (in red) identified by label-free quantitative (LFQ) mass-spectrometry (MS) after JUN IP in JUN sgRNA-expressing vs control SAOS-2 cells (**a**) or in SAOS-2 cells treated with Ponatinib for 3 h (**b**). Proteins that were identified in (**a**) and display reduced enrichment by JUN after ponatinib treatment are underlined. Specifically enriched proteins (numbered circles) are distinguished from background binders by a two-dimensional cut-off of >four- (**a**) or two-fold (**b**) enrichment and *P* < 0.01. Two-dimensional error bars represent the standard deviation based on iterative imputation cycles during the label-free analysis to substitute zero values (e.g., no detection in the JUN sg samples). **c** Viability assays of SAOS-2 cells treated with combinations of ponatinib and either triciribine or KU-60019. Values represent percentage of survival relative to DMSO-treated cells and are mean of two experiments performed in duplicates. Bliss synergy scores for each drug combination are calculated using Synergyfinder software^86^ (www.synergyfinder.org) and shown in the lower panels. A score >10 indicates synergy between the two drugs. **d** Western blot for γH2AX in SAOS-2 cells treated for 24 h with either ponatinib, triciribine (tri) or combinations of both. (β-actin = loading control). (*n* = 2). **e** Telomeric C-circle levels in SAOS-2 cells treated with either ponatinib (250 nM), triciribine (125 nM) or both for 72 h. Values (mean ± SD; two independent experiments with three biological replicates each) are represented as fold change relative to the corresponding DMSO control. (**P < 0.01, determined by two-tailed Mann–Whitney test). Source data are provided as a Source Data file.

To investigate this possibility further, we used label-free quantitative (LFQ) proteomics and identified proteins that interact with JUN in SAOS-2 cells (Fig. 7a and Supplementary Data 4), including known JUN interactors, such as AP-1 subunits (FOSL1, FOSB and FOS)^54^. Intriguingly, several DNA repair proteins were found to bind to JUN (e.g., XRCC6, XRCC5, RPA1, SMARCA5, LIG3, PARP2 and RPA2), as well as RUNX2, previously described to bind to telomeric DNA^55^. Importantly, interactions with some of the DNA repair proteins, but not with co-transcription factors, were disrupted after 3 h of ponatinib treatment (Fig. 7b). These results suggest that JUN may act directly on DNA repair processes to regulate ALT activity rather than indirectly as a transcription factor.

### Synergetic interactions between ponatinib and inhibitors of DNA repair or synthesis

Given that ponatinib is a multi-RTK inhibitor and the potential function of JUN in regulating DNA repair processes, we sought to identify synergistic drug interactions with ponatinib. We performed a drug library screen of anti-cancer compounds in the presence of DMSO as a control or two concentrations of ponatinib (Supplementary Fig. 12a–c). As expected, interactions with other RTK inhibitors (such as Afatinib and Dacomitinib) were identified and validated (Supplementary Fig. 12c, d). Interestingly, three out of the nine drugs identified (Supplementary Fig. 12c) were inhibitors of DNA repair proteins: ATM/DNA-PKcs (KU-60019^56^), CHK1 (LY2603618^57^), and DNA synthesis (Triciribine^58^). Synergism on cell killing for ponatinib was greater with triciribine and KU-60019 (Fig. 7c) than with LY2603618 (Supplementary Fig. 12d). Among these combinations, only triciribine synergized with low doses of ponatinib to induce DNA damage (Fig. 7d and Supplementary Fig. 12e). Likewise, treating SAOS-2 cells with a combination of ponatinib and triciribine exacerbated telomeric C-circle formation (Fig. 7e). These synergistic effects corroborate an impact of ponatinib on DNA homeostasis in ALT cells and an implication of DNA repair machineries in antagonizing this effect. Interestingly, the combination of ponatinib and triciribine had synergistic or additive effects in killing ALT cells but not telomerase-positive ones (Supplementary Fig. 13).

## Discussion

**Fig. 8:**
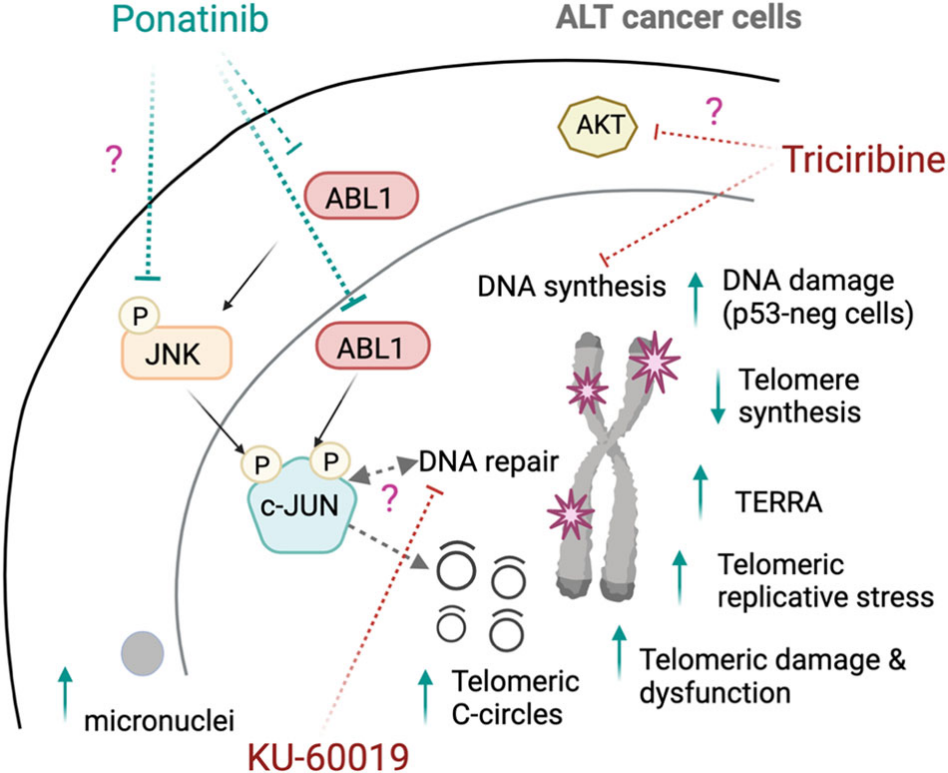
Schematic summary of mechanisms of action of ponatinib in ALT cells. Ponatinib disrupts alternative lengthening of telomeres mechanisms by inducing telomeric C-circles and telomere dysfunction concomitant with an inhibition of telomere synthesis in ALT cells. The effects of ponatinib on ALT activity are mediated by at least an inhibition of an ABL1-JNK-JUN signalling circuit leading to JUN degradation. Furthermore, synergistic combinations of ponatinib and either triciribine or KU-60019 could be effective on cancer cells relying on ALT. P phosphorylation, neg negative. Created with BioRender.com.

Contrary to telomerase-positive cells, telomeres in ALT cells are hot spots for replication stress and DNA damage and repair^7^. Here, we report that the tyrosine kinase inhibitor ponatinib exacerbates ALT telomere dysfunction and replicative stress and concomitantly interferes with telomere synthesis (Fig. 8). Importantly, a key finding in our study is the identification of ABL1-JNK-JUN signalling network that disrupts telomeric homeostasis and contributes to enhanced killing by ponatinib of ALT cells (Fig. 8). Replication problems at telomeres of ALT cells are mitigated by proteins which allow fork regression and resolution of replication stress, including FANCM^12,13,33^ and SMARCAL1^38,59^. Depletion of either proteins rapidly induces telomeric C-circles, telomere dysfunction and ALT activity^12,38^. These phenotypic effects are similar to those observed in ponatinib-treated ALT cells. However, in contrast to ponatinib, FANCM depletion causes increased telomere synthesis^12^. Moreover, DNA damage signalling or telomeric damage after inhibition of these proteins is also observed in telomerase-positive cells^12,60–62^. While ponatinib’s impact on telomeres and DNA damage is specific to ALT cells, potential inhibitory effects of ponatinib on either of these two proteins or related partners warrant further investigation, especially since FANCM depletion selectively affects ALT cell viability^12^.

Failure to resolve replication stress at telomeres leads to fork collapse and subsequent formation of double strand DNA breaks, which can prime for distinct homology-directed repair pathways and enable telomere maintenance via processes analogous to break-induced replication^8,10,43,63^. We showed that ALT cells treated with ponatinib accumulated telomeric replicative stress but were no longer able to engage in either productive telomere synthesis or extension mechanisms, in vitro. Whether ponatinib interferes with processing of telomeric recombination intermediates or specific machineries of break-induced replication remains to be determined. However, our results suggest that ponatinib increases unresolved replicative stress, leading to fork collapse and C-circle generation. The increase of extrachromosomal telomeric C-circles, the most robust ALT marker^24^, after ponatinib treatment was observed across all tested ALT cell lines, despite differences in other phenotypic effects induced by ponatinib. The latter differences could be attributed to inherent characteristics of each cell line, as well as to the different types of ALT pathways that can distinctively support telomere maintenance^64,65^.

While we did not observe an increase in APB formation, ponatinib treatment led to a significant and rapid inhibition of ALT-associated telomere synthesis occurring in APBs. This suggests that the ponatinib-induced increase of telomeric C-circles may originate from unresolved damage or replication defects at telomeres rather than from an increase in productive ALT activity.

In the last decades, several kinase inhibitors of signalling pathways sustaining cancer cell survival have been successfully developed and used in oncology therapies^66^. Concerning telomere biology, our understanding of the implication of extracellular or cytoplasmic signalling networks remains rather limited. Mostly, PI3K/AKT and RAS pathways have been shown to modulate telomeric protection in cancer cells, by reducing TRF1 protein levels^67–69^. We identified a signalling network that impacts telomeric C-circle generation and can account at least partially for the activity of ponatinib on ALT telomeres (Fig. 8). The effect of JUN on telomeric C-circle suppression seems to be independent of its transcriptional activity. Based on our interactome data and previous reports, JUN might be affecting DNA repair activity in ALT cells. In agreement with this hypothesis, several studies have previously linked JUN with the DNA damage response. In fact, JUN levels are rapidly induced by DNA damaging agents, such as exposure to either UV irradiation or H_2_O_2_ in human cells^70^. In murine embryonic fibroblasts, JUN deficiency leads to premature senescence and accumulation of spontaneous and genotoxic-induced DNA damage, suggesting reduced DNA repair capacity^71^. Interestingly, following UV irradiation, phosphorylated JUN interacts and colocalizes with PML in newly formed microspeckles^72^. Furthermore, JUN localizes with ATM and γH2AX at sites of DNA damage^71^ and interacts with proteins of the DNA repair machinery such as BLM^73,74^, BRCA1^75^, LIG3^76^, DNA–PKcs^77^ and XRCC6^78^.

A potential impairment of DNA repair by ponatinib is further supported by down-regulation of DNA repair genes in ponatinib-treated cells and by induction of DNA damage, which is not limited to telomeres, in p53-deficient ALT cells. In agreement with this, ponatinib synergized with DNA repair protein inhibitors (KU-60019 and LY2603618), as well as with a DNA synthesis inhibitor, the nucleoside analogue triciribine. Specifically, combining ponatinib with triciribine provoked higher levels of telomeric C-circles and DNA damage and was more effective in reducing proliferation of ALT but not telomerase-positive cells. Interestingly, triciribine has also been described to inhibit AKT signalling^79^. These results suggest that multiple signalling pathways may be cooperating in preserving telomeres of ALT cells and that deregulating ALT activity by ponatinib-based combinatorial treatments may represent one compelling therapeutic option for ALT cancers (Fig. 8).

Importantly, ponatinib is already FDA-approved^80^, and therefore, may be rapidly repurposed for ALT cancers. Moreover, the effects of ponatinib on cell death of ALT cells and their telomeric C-circles was not limited to sarcoma but was also observed in ALT glioma stem cells (GSCs) (TG20 cells)^81,82^ (Supplementary Fig. 14a–c). Finally, the demonstrated effects of targeting a signalling circuit in ALT cancers on DNA damage and generation of C-circles which, in turn, potentially generate immune responses, are likely to become clinically relevant in the context of an emerging immuno-oncology field^83^.

## Methods

### Drug library screens

In all, 600–1000 SW26, SW39 or SAOS-2 cells were plated in 384-well plates. The following day, 317 drugs from the Selleckchem anti-cancer compound library (Selleckchem) were added using an Agilent Bravo Automated Liquid Handling Platform (Agilent) at a final concentration of 1 µM. For drug synergy studies, DMSO or ponatinib at 75 nM or 150 nM were additionally added to the whole plate. Three days post-treatment, CellTiter-Glo® reagent (Promega) was added using a MultiFlo Microplate Dispenser (BioTek), and luminescence was measured with an Infinite M1000 Pro Microplate Reader (Tecan).

### In vivo experiments

In total, 3 × 10^6^ CAL72 cells mixed with Matrigel matrix (Corning) were inoculated subcutaneously in the flank of 6–8 weeks old female NSG (NOD-SCID gamma) mice. DMSO (vehicle control), ponatinib or PD173074 diluted in Citrate buffer (25 mM, pH=4) were administered by oral gavage on alternate days. At the end of the experiment, mice were sacrificed; tumors were collected and weighed. In vivo experiments were performed in compliance with ethical regulations of Institutional Animal Care and Use Committee (IACUC) of National University of Singapore.

### Immunoprecipitation experiments

Fresh nuclear protein extracts were obtained using the NE-PER™ nuclear and cytoplasmic extraction reagents (ThermoFisher Scientific) and quantified. Antibodies (0.5–2 µg) were coupled to Dynabeads™ protein G (Life Technologies) by incubation for 30 min at room temperature on a rotating wheel. Coupled beads were then washed and incubated on a rotator for 2 h at 4 °C with 150–300 µg of nuclear extracts diluted in protein binding buffer (PBB) (150 mM NaCl, 50 mM Tris, 5 mM MgCl_2_, 0.25% NP40, 1 mM DTT) supplemented with protease and phosphatase inhibitors. The beads were then washed thrice with cold PBB, and proteins were eluted by adding SDS loading buffer (3×) to the beads and heating for 10 min at 95 °C.

### Combined fluorescence in situ hybridization and immunostaining (FISH-IF)

Cells were fixed with 4% formaldehyde, permeabilized with PBS and 0.5% Triton-X for 15 min and blocked with 3% BSA and 0.1% Triton-X in PBS for 30 min before dehydrating with 50%, 80% and 100% ethanol at 5-min intervals. The telomeric PNA probes (Alexa488-O-O-(CCCTAA)_3_ (TelC) or Cy3-O-O-(TTAGGG)_3_ (TelG)) (Panagene) diluted in a hybridization buffer (70% formamide, 10 mM Tris pH = 7.2, 1% BSA) were added and slides were then denatured for 5 min at 80 °C. Following denaturation, the slides were hybridized at room temperature for 2 h in the dark. A series of washes were performed with wash buffer 1 (70% formamide, 10 mM Tris pH = 7.2; two washes of 15 min each) and wash buffer 2 (50 mM Tris pH = 7.2, 150 mM NaCl, 0.05% Tween20; two washes of 5 min each), before re-fixation with 4% PFA for 5 min, permeabilization for 5 min and blocking for 30 min. Subsequent antibody staining was then performed as described in the Supplementary Methods.

### Telomere restriction fragment (TRF) analysis

DNA was extracted from cell pellets using DNeasy Blood & Tissue Kit (Qiagen) and quantified using the Qubit™ 1X dsDNA HS Assay Kit (Invitrogen). TRF was performed using the TeloTAGGG telomere length Assay kit (Roche). DNA samples were digested with restriction enzymes HinfI and RsaI enzymes (New England Biolabs) for 6 h at 37 °C and pulse-field gel electrophoresis (PFGE) was then performed using 1–3 µg of digested DNA in 0.8% megabase agarose (Bio-Rad) gel at 3 V/cm for 17 h in a CHEF-DR II system (Bio-Rad). The digestion efficiency was verified by staining the gel with 0.5 µg/mL ethidium bromide (Bio-Rad) in water. The gel was then de-stained with water, washed with HCl solution, denatured and neutralized, as per the TELOTAGGG Telomere Length Assay kit (Roche) instructions. DNA was transferred onto a Hybond-N + nylon membrane (GE Healthcare) by capillary transfer overnight using saline-sodium citrate (20X SSC). Once transferred onto the membrane, UV crosslinking was performed twice at 120 mJ using HL-2000 Hybrilinker (UVP Lab Products), followed by the steps according to the TELOTAGGG Telomere Length Assay kit manual. Average telomere length estimation was performed using the TeloTool software^84^.

### Telomeric C-circle assay

Cells were seeded into 6-well plates at 1 × 10^5^ cells per well and collected after 72 h of culture or treatment. Genomic DNA was extracted using DNeasy Blood & Tissue Kit (Qiagen), and subsequently quantified using the Qubit™ 1X dsDNA HS Assay Kit (Invitrogen). Overall, 50–75 ng of DNA was digested with restriction enzymes HinfI and RsaI (New England Biolabs) for 2 h at 37 °C. Following digestion, rolling circle amplification reactions on 5–7.5 ng of the digested DNA samples were performed using ɸ29 polymerase in ɸ29 buffer, 0.1 mg/mL BSA and 2 mM dATP, dGTP, dTTP (New England Biolabs). Reactions without the ɸ29 polymerase enzyme were performed as a negative control. Amplification was conducted for 5–6 h at 30 °C and then stopped at 70 °C for 20 min. Following amplification, 1–5 µL of the reaction was diluted in saline-sodium citrate (2X SSC) and dot blotted on a Hybond-N + nylon membrane (GE Healthcare) either manually or using a 96-well dotBLOT apparatus (Cleaver Scientific). The membrane was then cross-linked by ultraviolet radiation at 120 mJ, rinsed with 2× SSC, and hybridization with a telomere probe was then performed as instructed by the TeloTAGGG Telomere Length Assay kit (Roche).

### BrdU pull-down for detection of newly synthesized telomeric DNA

BrdU (5-Bromo-2′-deoxyuridine) (Santa Cruz) was added at 100 µM to cell cultures for 2 h. Cells were collected afterwards, and genomic DNA was extracted using DNeasy Blood and Tissue kit (Qiagen). Samples were sonicated using Vibra-Cell™ ultrasonic processor (Sonics) and fragments sizes (100–500 bp) were verified by gel electrophoresis. Sheared DNA was quantified using the Qubit™ 1× dsDNA HS Assay Kit (Invitrogen) and 1–4 µg of sonicated DNA were denatured for 10 min at 95 °C and cooled immediately on ice. Samples were then incubated with 2 µg of either anti-BrdU antibody (IIB5) or anti-IgG control antibody diluted in immunoprecipitation buffer (IP) (0.0625% (v/v) Triton-X-100 in PBS) and incubated overnight on a turning wheel at 4 °C. The following day, 15 µl of prewashed Dynabeads™ protein G (Life Technologies) were added, and reactions were further rotated for one hour at 4 °C. Beads were then washed thrice with IP buffer, once with TE buffer and incubated with 100 µL of elution buffer (1% (w/v) SDS in TE) twice for 15 min at 65 °C. Eluted DNA was then purified using QIAquick PCR purification kit (Qiagen) and subjected to a telomeric dot blot in denaturing conditions as described above, together with input samples. For detection of Alu repeats, oligonucleotides (GTGATCCGCCCGCCTCGGCCTCCCAAAGTG) were purchased from IDT and 50 pmol were tail-labelled with digoxigenin (DIG) using 100 U of terminal transferase (New England Biolabs), 0.05 mM of DIG-ddUTP (Roche) and dATP.

### ATSA (ALT telomere DNA synthesis in APBs) assay

ATSA assay was performed according to Zhang et al.^43^ to detect telomere synthesis in APBs in G2 cells. U2OS or SAOS-2 cells were seeded in chamber slides and treated the next day first with either DMSO or ponatinib, then after 3 h, a CDK1 inhibitor (Ro-3306; MedchemExpress) was added for 15–16 h to synchronize the cells in G2 phase. EdU was then added for 2 h, and cells were fixed with 4% formaldehyde for 10 min, permeabilized for 15 min with PBS Triton-X 0.5% and stained for telomeric DNA (using a TelG-PNA-probe) as per the FISH protocol. After telomere staining, cells were fixed again, permeabilized and EdU was stained using a Click reaction according to the manufacturer’s protocol (Click-&-Go Plus EdU 647; Click Chemistry Tools). The slides were fixed again, permeabilized and stained for PML bodies (using an antibody against SP100, a component of the PML bodies). Images were captured with a LSM710 confocal microscope and APB-positive cells were scored for their total number of APBs as well as the EdU+ APBs.

### Reagents and additional methods

List of reagents (primers, gRNA, plasmids and antibodies) can be found in Supplementary Data 5 and additional methods are described in the Supplementary Information file.

### Statistics and reproducibility

Statistical analyses as well as number of replicates and independent reproducible experiments are described in each figure legends for the corresponding assays. GraphPad Prism 9.4.1 was used for data presentation and statistical analyses.

### Reporting summary

Further information on research design is available in the Nature Portfolio Reporting Summary linked to this article.

## Supplementary information

Supplementary Information

Peer Review File

Description of Additional Supplementary Files

Supplementary Data 1

Supplementary Data 2

Supplementary Data 3

Supplementary Data 4

Supplementary Data 5

Reporting Summary

## Data Availability

The RNA-sequencing data have been deposited to NCBI’s Gene Expression Omnibus and are accessible through GEO Series accession number GSE190438. The mass-spectrometry data was deposited to the ProteomeXchange Consortium via PRIDE^85^ under the accession number PXD037501. Source data are provided with this paper.

## Source data

Source Data

## References

[1] Harley CB, Futcher AB, Greider CW (1990). Telomeres shorten during ageing of human fibroblasts. Nature.

[2] Lundblad V, Szostak JW (1989). A mutant with a defect in telomere elongation leads to senescence in yeast. Cell.

[3] Kim NW (1994). Specific association of human telomerase activity with immortal cells and cancer. Science.

[4] Bryan TM (1997). Evidence for an alternative mechanism for maintaining telomere length in human tumors and tumor-derived cell lines. Nat. Med..

[5] Bryan TM (1995). Telomere elongation in immortal human cells without detectable telomerase activity. EMBO J..

[6] Heaphy CM (2011). Prevalence of the alternative lengthening of telomeres telomere maintenance mechanism in human cancer subtypes. Am. J. Pathol..

[7] Cesare AJ (2009). Spontaneous occurrence of telomeric DNA damage response in the absence of chromosome fusions. Nat. Struct. Mol. Biol..

[8] Cho NW (2014). Interchromosomal homology searches drive directional ALT telomere movement and synapsis. Cell.

[9] Dunham MA (2000). Telomere maintenance by recombination in human cells. Nat. Genet..

[10] Dilley RL (2016). Break-induced telomere synthesis underlies alternative telomere maintenance. Nature.

[11] Flynn RL (2015). Alternative lengthening of telomeres renders cancer cells hypersensitive to ATR inhibitors. Science.

[12] Lu R (2019). The FANCM-BLM-TOP3A-RMI complex suppresses alternative lengthening of telomeres (ALT). Nat. Commun..

[13] Pan X (2017). FANCM, BRCA1, and BLM cooperatively resolve the replication stress at the ALT telomeres. Proc. Natl Acad. Sci. USA.

[14] Episkopou H (2019). TSPYL5 depletion induces specific death of ALT cells through USP7-dependent proteasomal degradation of POT1. Mol. Cell.

[15] Hoang SM (2020). Regulation of ALT-associated homology-directed repair by polyADP-ribosylation. Nat. Struct. Mol. Biol..

[16] Ge Y (2019). Inhibition of p53 and/or AKT as a new therapeutic approach specifically targeting ALT cancers. Protein Cell.

[17] Bakhos-Douaihy D (2019). ALT cancer cells are specifically sensitive to lysine acetyl transferase inhibition. Oncotarget.

[18] Zheng, X. H. et al. A cisplatin derivative tetra-Pt(bpy) as an oncotherapeutic agent for targeting ALT cancer. *J. Natl Cancer Inst.* **109**, djx061 (2017). 10.1093/jnci/djx061 28521363

[19] Koneru, B. et al. ALT neuroblastoma chemoresistance due to telomere dysfunction-induced ATM activation is reversible with ATM inhibitor AZD0156. *Sci. Transl. Med.* **13**, eabd5750 (2021). 10.1126/scitranslmed.abd5750 PMC9208664 34408079

[20] Bechter OE (2003). Homologous recombination in human telomerase-positive and ALT cells occurs with the same frequency. EMBO Rep..

[21] Huang, W. S. et al. Discovery of 3-[2-(imidazo[1,2-b]pyridazin-3-yl)ethynyl]−4-methyl-N-{4-[(4-methylpiperazin-1-yl)methyl]−3-(trifluoromethyl)phenyl}benzamide (AP24534), a potent, orally active pan-inhibitor of breakpoint cluster region-abelson (BCR-ABL) kinase including the T315I gatekeeper mutant. *J. Med. Chem.* **53**, 4701–4719 (2010). 10.1021/jm100395q 20513156

[22] O’Hare T (2009). AP24534, a pan-BCR-ABL inhibitor for chronic myeloid leukemia, potently inhibits the T315I mutant and overcomes mutation-based resistance. Cancer Cell.

[23] Mohammadi M (1998). Crystal structure of an angiogenesis inhibitor bound to the FGF receptor tyrosine kinase domain. EMBO J..

[24] Henson JD (2009). DNA C-circles are specific and quantifiable markers of alternative-lengthening-of-telomeres activity. Nat. Biotechnol..

[25] Tomaska L (2009). Telomeric circles: universal players in telomere maintenance?. Nat. Struct. Mol. Biol..

[26] Wang RC, Smogorzewska A, de Lange T (2004). Homologous recombination generates T-loop-sized deletions at human telomeres. Cell.

[27] Mazzucco G (2020). Telomere damage induces internal loops that generate telomeric circles. Nat. Commun..

[28] Zhang T (2019). Strand break-induced replication fork collapse leads to C-circles, C-overhangs and telomeric recombination. PLoS Genet..

[29] Draskovic I (2009). Probing PML body function in ALT cells reveals spatiotemporal requirements for telomere recombination. Proc. Natl Acad. Sci. USA.

[30] Yeager TR (1999). Telomerase-negative immortalized human cells contain a novel type of promyelocytic leukemia (PML) body. Cancer Res..

[31] Takai H, Smogorzewska A, de Lange T (2003). DNA damage foci at dysfunctional telomeres. Curr. Biol..

[32] Porro A (2014). Functional characterization of the TERRA transcriptome at damaged telomeres. Nat. Commun..

[33] Silva B (2019). FANCM limits ALT activity by restricting telomeric replication stress induced by deregulated BLM and R-loops. Nat. Commun..

[34] Lovejoy CA (2012). Loss of ATRX, genome instability, and an altered DNA damage response are hallmarks of the alternative lengthening of telomeres pathway. PLoS Genet..

[35] Anantha RW, Vassin VM, Borowiec JA (2007). Sequential and synergistic modification of human RPA stimulates chromosomal DNA repair. J. Biol. Chem..

[36] Oakley GG, Patrick SM (2010). Replication protein A: directing traffic at the intersection of replication and repair. Front. Biosci..

[37] Olson E (2006). RPA2 is a direct downstream target for ATR to regulate the S-phase checkpoint. J. Biol. Chem..

[38] Cox KE, Marechal A, Flynn RL (2016). SMARCAL1 resolves replication stress at ALT telomeres. Cell Rep..

[39] Abbadie C, Pluquet O (2020). Unfolded protein response (UPR) controls major senescence hallmarks. Trends Biochem. Sci..

[40] Lin HK (2010). Skp2 targeting suppresses tumorigenesis by Arf-p53-independent cellular senescence. Nature.

[41] Pluquet O, Pourtier A, Abbadie C (2015). The unfolded protein response and cellular senescence. A review in the theme: cellular mechanisms of endoplasmic reticulum stress signaling in health and disease. Am. J. Physiol. Cell Physiol..

[42] Zoumpoulidou G (2021). Therapeutic vulnerability to PARP1,2 inhibition in RB1-mutant osteosarcoma. Nat. Commun..

[43] Zhang JM (2019). Alternative lengthening of telomeres through two distinct break-induced replication pathways. Cell Rep..

[44] Lauvrak SU (2013). Functional characterisation of osteosarcoma cell lines and identification of mRNAs and miRNAs associated with aggressive cancer phenotypes. Br. J. Cancer.

[45] Sun GD (2007). The endoplasmic reticulum stress-inducible protein Niban regulates eIF2alpha and S6K1/4E-BP1 phosphorylation. Biochem. Biophys. Res. Commun..

[46] Lin A (1992). Casein kinase II is a negative regulator of c-Jun DNA binding and AP-1 activity. Cell.

[47] Karin M, Hunter T (1995). Transcriptional control by protein phosphorylation: signal transmission from the cell surface to the nucleus. Curr. Biol..

[48] Musti AM, Treier M, Bohmann D (1997). Reduced ubiquitin-dependent degradation of c-Jun after phosphorylation by MAP kinases. Science.

[49] Pulverer BJ (1991). Phosphorylation of c-jun mediated by MAP kinases. Nature.

[50] Smeal T (1991). Oncogenic and transcriptional cooperation with Ha-Ras requires phosphorylation of c-Jun on serines 63 and 73. Nature.

[51] Barila D (2000). A nuclear tyrosine phosphorylation circuit: c-Jun as an activator and substrate of c-Abl and JNK. EMBO J..

[52] Zhang T (2012). Discovery of potent and selective covalent inhibitors of JNK. Chem. Biol..

[53] Angel P (1988). Oncogene jun encodes a sequence-specific trans-activator similar to AP-1. Nature.

[54] Huttlin EL (2021). Dual proteome-scale networks reveal cell-specific remodeling of the human interactome. Cell.

[55] Kappei D (2017). Phylointeractomics reconstructs functional evolution of protein binding. Nat. Commun..

[56] Golding SE (2009). Improved ATM kinase inhibitor KU-60019 radiosensitizes glioma cells, compromises insulin, AKT and ERK prosurvival signaling, and inhibits migration and invasion. Mol. Cancer Ther..

[57] King C (2014). Characterization and preclinical development of LY2603618: a selective and potent Chk1 inhibitor. Invest. New Drugs.

[58] Wotring LL (1990). Dual mechanisms of inhibition of DNA synthesis by triciribine. Cancer Res..

[59] Feng, E. et al. CSB cooperates with SMARCAL1 to maintain telomere stability in ALT cells. *J. Cell Sci.* **133**, jcs234914 (2020). 10.1242/jcs.234914 31974116

[60] Blackford AN (2012). The DNA translocase activity of FANCM protects stalled replication forks. Hum. Mol. Genet..

[61] Collis SJ (2008). FANCM and FAAP24 function in ATR-mediated checkpoint signaling independently of the Fanconi anemia core complex. Mol. Cell.

[62] Poole LA (2015). SMARCAL1 maintains telomere integrity during DNA replication. Proc. Natl Acad. Sci. USA.

[63] Min, J., Wright, W. E. & Shay, J. W. Alternative lengthening of telomeres mediated by mitotic DNA synthesis engages break-induced replication processes. *Mol. Cell. Biol.* **37**, e00226-17 (2017). 10.1128/MCB.00226-17 PMC5615184 28760773

[64] Sobinoff AP, Pickett HA (2017). Alternative lengthening of telomeres: DNA repair pathways converge. Trends Genet..

[65] Sobinoff AP, Pickett HA (2020). Mechanisms that drive telomere maintenance and recombination in human cancers. Curr. Opin. Genet. Dev..

[66] Roskoski R (2021). Properties of FDA-approved small molecule protein kinase inhibitors: a 2021 update. Pharmacol. Res..

[67] Bejarano L (2019). Multiple cancer pathways regulate telomere protection. EMBO Mol. Med..

[68] Mendez-Pertuz M (2017). Modulation of telomere protection by the PI3K/AKT pathway. Nat. Commun..

[69] Sanchez-Vazquez R, Martinez P, Blasco MA (2021). AKT-dependent signaling of extracellular cues through telomeres impact on tumorigenesis. PLoS Genet..

[70] Devary Y (1991). Rapid and preferential activation of the c-jun gene during the mammalian UV response. Mol. Cell. Biol..

[71] MacLaren A (2004). c-Jun-deficient cells undergo premature senescence as a result of spontaneous DNA damage accumulation. Mol. Cell. Biol..

[72] Salomoni P (2005). The promyelocytic leukemia protein PML regulates c-Jun function in response to DNA damage. Blood.

[73] Chandra S (2013). Enhancement of c-Myc degradation by BLM helicase leads to delayed tumor initiation. J. Cell. Sci..

[74] Priyadarshini R (2018). BLM potentiates c-Jun degradation and alters its function as an oncogenic transcription factor. Cell. Rep..

[75] Hu YF, Li R (2002). JunB potentiates function of BRCA1 activation domain 1 (AD1) through a coiled-coil-mediated interaction. Genes Dev..

[76] Li X (2015). Proteomic analyses reveal distinct chromatin-associated and soluble transcription factor complexes. Mol. Syst. Biol..

[77] Bannister AJ (1993). c-Jun is phosphorylated by the DNA-dependent protein kinase in vitro; definition of the minimal kinase recognition motif. Nucleic Acids Res..

[78] Abbasi S, Schild-Poulter C (2019). Mapping the Ku interactome using proximity-dependent biotin identification in human cells. J. Proteome Res..

[79] Yang L (2004). Akt/protein kinase B signaling inhibitor-2, a selective small molecule inhibitor of Akt signaling with antitumor activity in cancer cells overexpressing Akt. Cancer Res..

[80] Luciano L (2020). The multi-tyrosine kinase inhibitor ponatinib for chronic myeloid leukemia: real-world data. Eur. J. Haematol..

[81] Jeitany M (2017). Opposite effects of GCN5 and PCAF knockdowns on the alternative mechanism of telomere maintenance. Oncotarget.

[82] Jeitany M (2015). A preclinical mouse model of glioma with an alternative mechanism of telomere maintenance (ALT). Int. J. Cancer.

[83] Chabanon RM (2021). Targeting the DNA damage response in immuno-oncology: developments and opportunities. Nat. Rev. Cancer.

[84] Gohring J (2014). TeloTool: a new tool for telomere length measurement from terminal restriction fragment analysis with improved probe intensity correction. Nucleic Acids Res..

[85] Vizcaino JA (2016). 2016 update of the PRIDE database and its related tools. Nucleic Acids Res..

[86] Zheng S (2022). SynergyFinder plus: toward better interpretation and annotation of drug combination screening datasets. Genomics Proteomics Bioinforma..

