# Supplementary material for: Signalling inhibition by ponatinib disrupts productive alternative lengthening of telomeres (ALT)
Source: Nat Commun. 2023 Apr 6;14:1919. doi: 10.1038/s41467-023-37633-3 (PMC10079688; doi:10.1038/s41467-023-37633-3)
Supplement: Supplementary file 1 — Supplementary Information [file 41467_2023_37633_MOESM1_ESM.pdf]

Supplementary Information for:

Signalling Inhibition by Ponatinib Disrupts Productive Alternative Lengthening of Telomeres (ALT)

Frances Karla Kusuma[1†], Aishvaryaa Prabhu[2†], Galen Tieo[1], Syed Moiz Ahmed[1], Pushkar Dakle[2], Wai Khang Yong[2,3], Elina Pathak[4], Vikas Madan[2], Yan Yi Jiang[2,5], Wai Leong Tam[1,2,3,4], Dennis Kappei[2,3,6], Peter Dröge[1], H. Phillip Koeffler[2,7,8], Maya Jeitany[1*].

1 School of Biological Sciences, Nanyang Technological University; Singapore.

2 Cancer Science Institute of Singapore, National University of Singapore; Singapore.

3 Department of Biochemistry, Yong Loo Lin School of Medicine, National University of Singapore; Singapore.

4 Genome Institute of Singapore, Agency for Science, Technology and Research (A*STAR); Singapore.

5 Current address: Hefei Institutes of Physical Science, Chinese Academy of Sciences, Hefei; China/P. R. China

6 NUS Center for Cancer Research, Yong Loo Lin School of Medicine, National University of Singapore; Singapore.

7 Cedars-Sinai Medical Center, Division of Hematology/Oncology, UCLA School of Medicine; Los Angeles, CA, USA.

8 Department of Hematology-Oncology, National University Cancer Institute of Singapore (NCIS), National University Hospital; Singapore.

* Correspondence to: maya.jeitany@gmail.com

† These authors contributed equally to this work.

This file contains:

- Supplementary Figures 1-14
- Supplementary Methods
- Supplementary References

Supplementary Figure 1

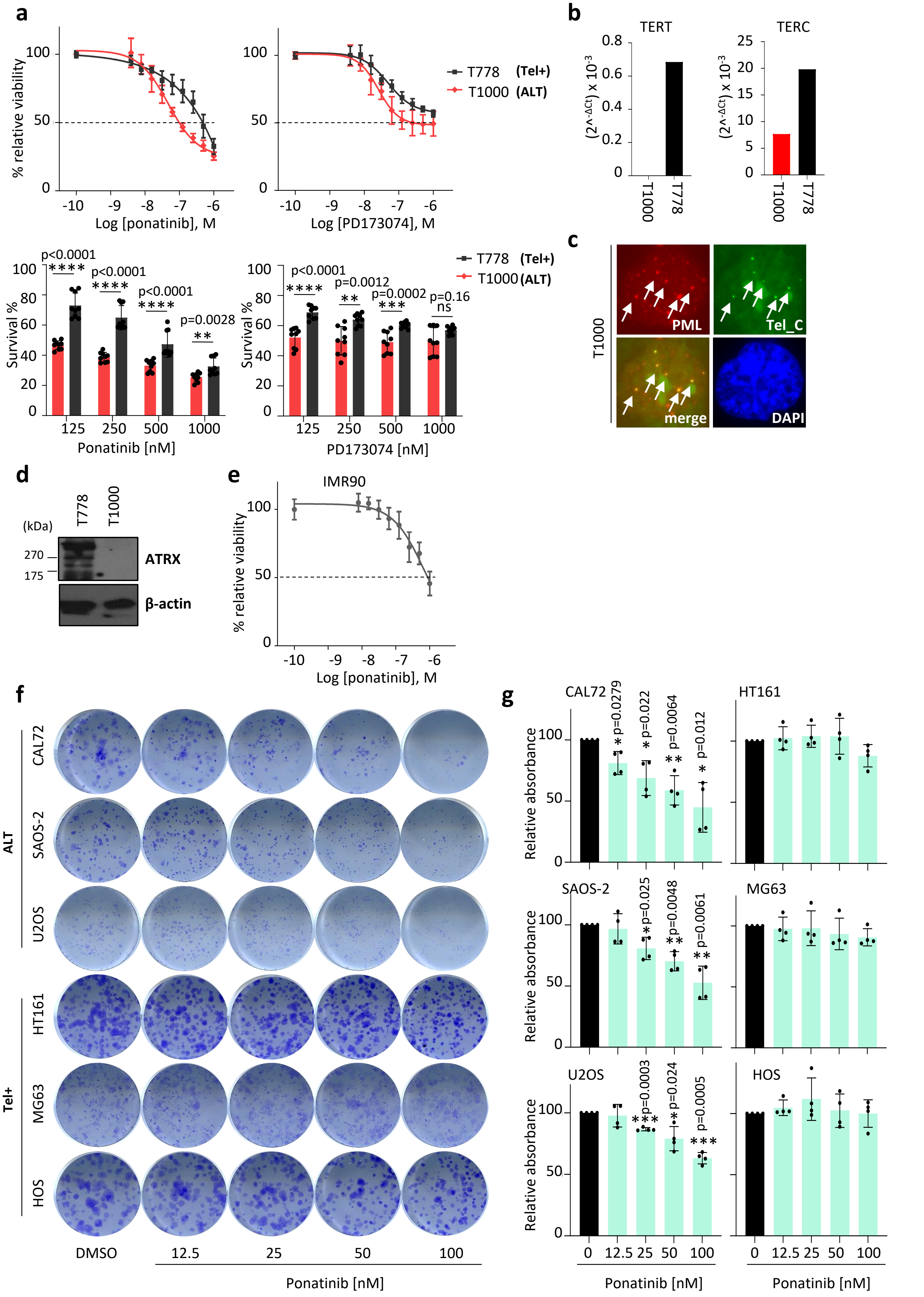

Supplementary Figure 1:

a) Viability assays of two liposarcoma cell lines: T1000 (ALT) and T778 (Tel+) treated with increasing concentrations of either ponatinib or PD173074. Values represent relative viability (mean ± SD of three independent experiments each performed in three biological replicates, n=9). Lower graphs show percentage of survival for selected concentrations. (****p<0.0001, ***p<0.001, **p<0.01, ns=not significant, as determined by two-tailed Mann-Whitney test).

b-d) Characterization of ALT phenotype in T1000 cells. **b)** RNA expression levels of telomerase subunits TERT and TERC relatively to GAPDH by Real-Time quantitative PCR. **c)** Example of presence of ALT-associated PML bodies (APBs) in T1000 ALT cell line. Arrows show colocalization of telomeres and PML bodies detected by combined immunostaining of PML and FISH of telomeres. **d)** Western blot for ATRX in T778 and T1000 cells.

e) Viability assays of IMR90 cells after treatment with different concentrations of ponatinib for 72 hours. Values are relative viability (mean ± SD of three experiments each performed in triplicates).

f) Representative images of clonogenic assays of osteosarcoma ALT (CAL72, SAOS-2 and U2OS) or telomerase-positive (HT161, MG63 and HOS) cell lines and their response to different concentrations of ponatinib.

g) Quantification of clonogenic assays in (e) showing the relative absorbance of each condition. Values are mean ± SD of 4 biological replicates from two independent experiments. (***p<0.001, **p<0.01, *p<0.05, as determined by two-tailed paired t-test).

Source data are provided as a Source Data file.

Supplementary Figure 2

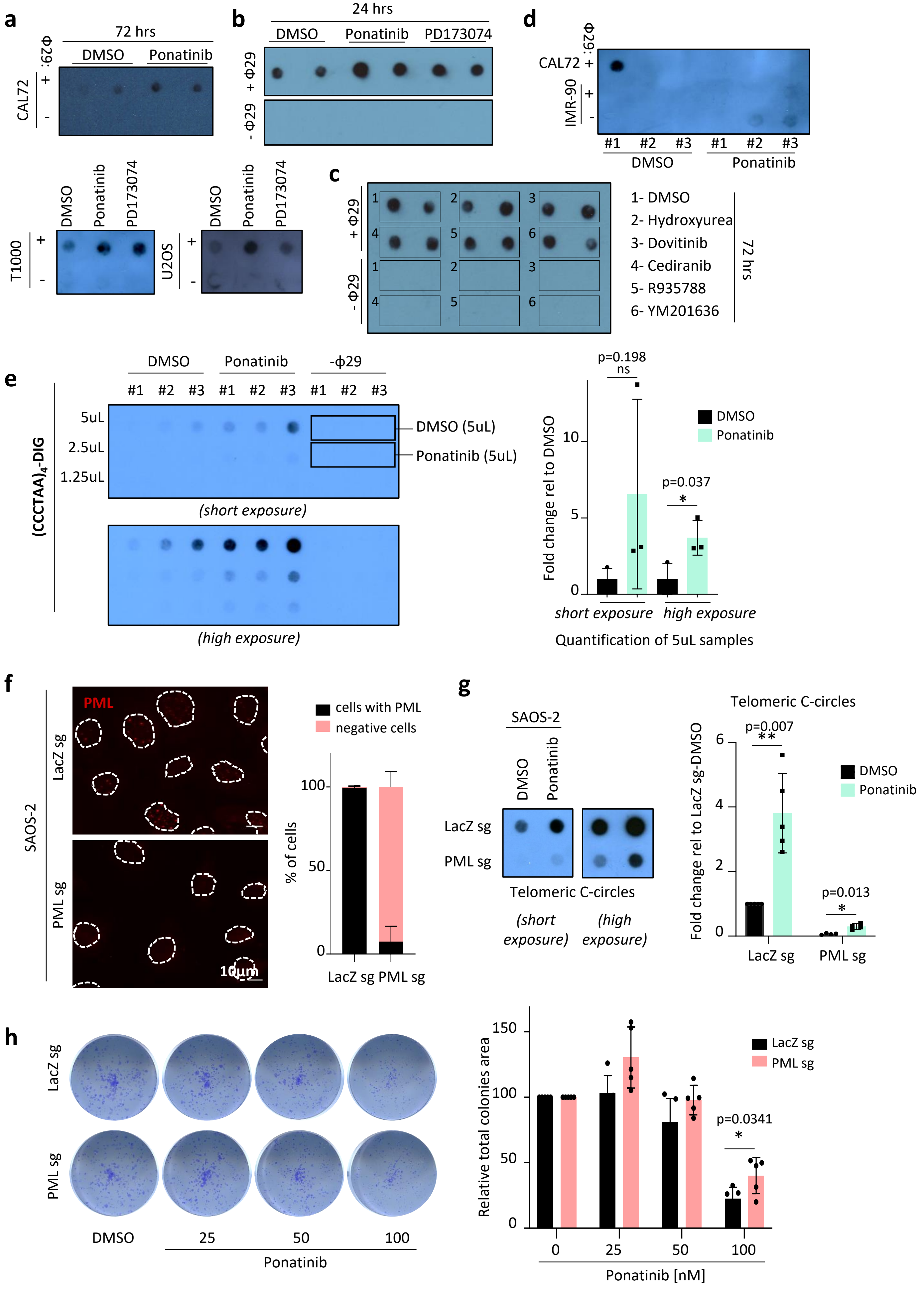

Supplementary Figure 2:

a) Representative dot blot for telomeric C-circle assays in several ALT cell lines treated for 72 hours with either ponatinib or PD173074.

b) Telomeric C-circle assay in SAOS-2 cells after 24 hours of drug treatment.

c) Telomeric C-circle assay in SAOS-2 cells treated for 72 hours with either DMSO (control), hydroxyurea (2 mM), dovitinib (500 nM), cediranib (500 nM), R778 (500 nM) or YM201636 (500 nM).

d) Telomeric C-circle assay in IMR90 cells after 72 hours of drug treatment. CAL72 serves as a positive control (#1, #2 and #3 represent three biological replicates).

e) Validation of telomeric C-circle assay in SAOS-2 cells. Different amounts (1.25, 2.5 or 5 µL) of the 20 µL rolling circle amplification reaction were loaded, and the membrane was hybridized using a DIG-labelled $(CCCTAA)_4$ probe. The graph represents the relative levels of C-circles of the 5 µL samples quantified on both short and high exposures. (*p<0.01, ns= not significant as determined by two-tailed unpaired t-test; n=3 biologically independent replicates; error bars represent ± SD).

f) Validation of PML depletion in SAOS-2 cells transduced with CRISPR-Cas9 and either LacZ or PML sgRNAs. The graph shows the percentage of cells positive or negative for PML immunostaining (red) in each condition. Nuclei are demarcated with dashed lines. A total of 361 and 285 cells from two independent experiments were counted for LacZ sg and PML sg cell lines respectively.

g) C-circle assays on SAOS-2 LacZ sg or PML sg cells after 72 hours of treatment with ponatinib (250 nM). The graph shows fold change of telomeric C-circles relative to LacZ sg-DMSO condition. (**p<0.001, *p<0.01, as determined by two-tailed paired t-test; n=5 for lacZ sg DMSO and ponatinib treated cells, and n=4 for PML sg cells; error bars are ± SD).

h) Colony formation assays on PML-deficient SAOS-2 cells treated with ponatinib. The right graph shows quantification of total colonies area relative to the 0 nM condition for each cell line calculated by ImageJ from 5 independent experiments. (*p<0.01, as determined by two-tailed paired t-test).

Source data are provided as a Source Data file.

Supplementary Figure 3

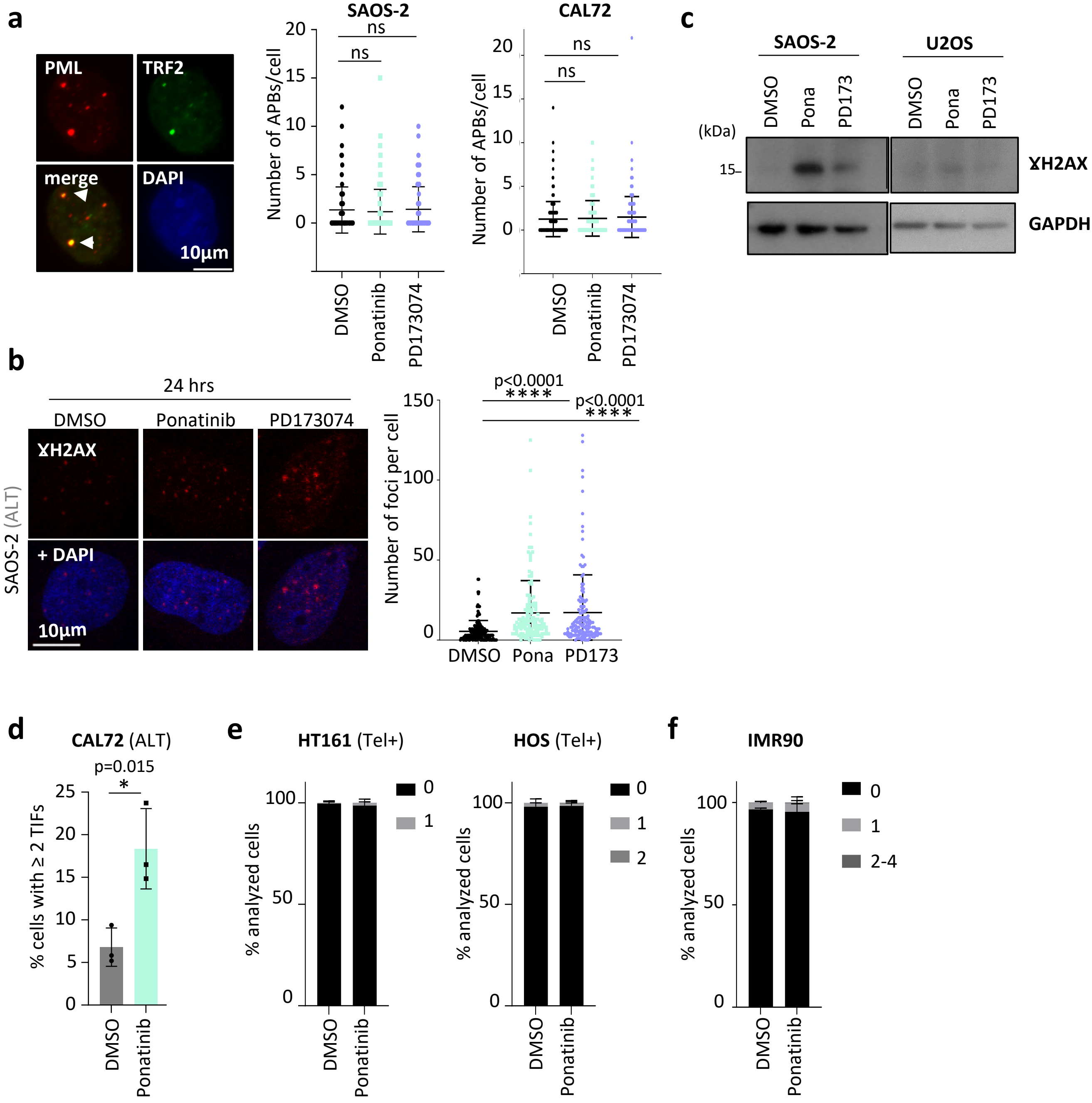

Supplementary Figure 3:

a) Detection of ALT-associated PML bodies (APBs) by co-immunostaining of PML and TRF2 in SAOS-2 cells treated with either ponatinib (250 nM) or PD173074 (500 nM) and in CAL72 cells treated with ponatinib (125 nM) or PD173074 (125 nM) for 24 hours. Graphs depict number of APBs per cell. (Number of analysed cells=188; 186 and 116 for SAOS-2 DMSO, ponatinib and PD173074, respectively and n= 267, 337 and 220 for CAL72 DMSO, ponatinib and PD173074, respectively). Data is from two biologically independent replicates and error bars represent mean ± SD (ns = not significant, as determined by two-tailed Mann-Whitney test).

b) Detection of γH2AX foci by immunostaining of SAOS-2 cells treated with either DMSO (n=162), ponatinib (250 nM; n=131) or PD173074 (500 nM; n=142) for 24 hours. Number of foci per cell was quantified using Fiji software. Error bars in the graph represent mean ± SD. (****$p<0.0001$, as determined by two-tailed unpaired t-test).

c) Western blot for γH2AX in cells treated with either ponatinib (250 nM) or PD173074 (500nM) for 24 hours. (n=2).

d) Telomere dysfunction-induced foci (TIFs) in CAL72 cells treated with ponatinib (125 nM) for 48 hours. Values are mean ± SD of 3 biological replicates from two independent experiments. Total number of analysed cells is 295 for DMSO and 289 for ponatinib-treated cells. (*$p<0.05$, as determined by two-tailed paired t-test).

e) Telomere dysfunction-induced foci in HT161 and HOS cells treated with ponatinib (250 nM) for 48 hours. The graphs show the percentage of cells (mean ± SD) with 0, 1 or 2 foci (4 biological replicates from two independent experiments). Total number of analysed cells is: DMSO (n=469), ponatinib (n=440) for HT161 and DMSO (n=491), ponatinib (n=438) for HOS.

f) Telomere dysfunction-induced foci in IMR90 cells treated with ponatinib (250 nM) for 48 hours. The graphs show the percentage of cells (mean ± SD) with 0, 1 or 2-4 foci (3 biological replicates). Total number of analysed cells is: DMSO (n=272) and ponatinib (n=232).

Source data are provided as a Source Data file.

Supplementary Figure 4

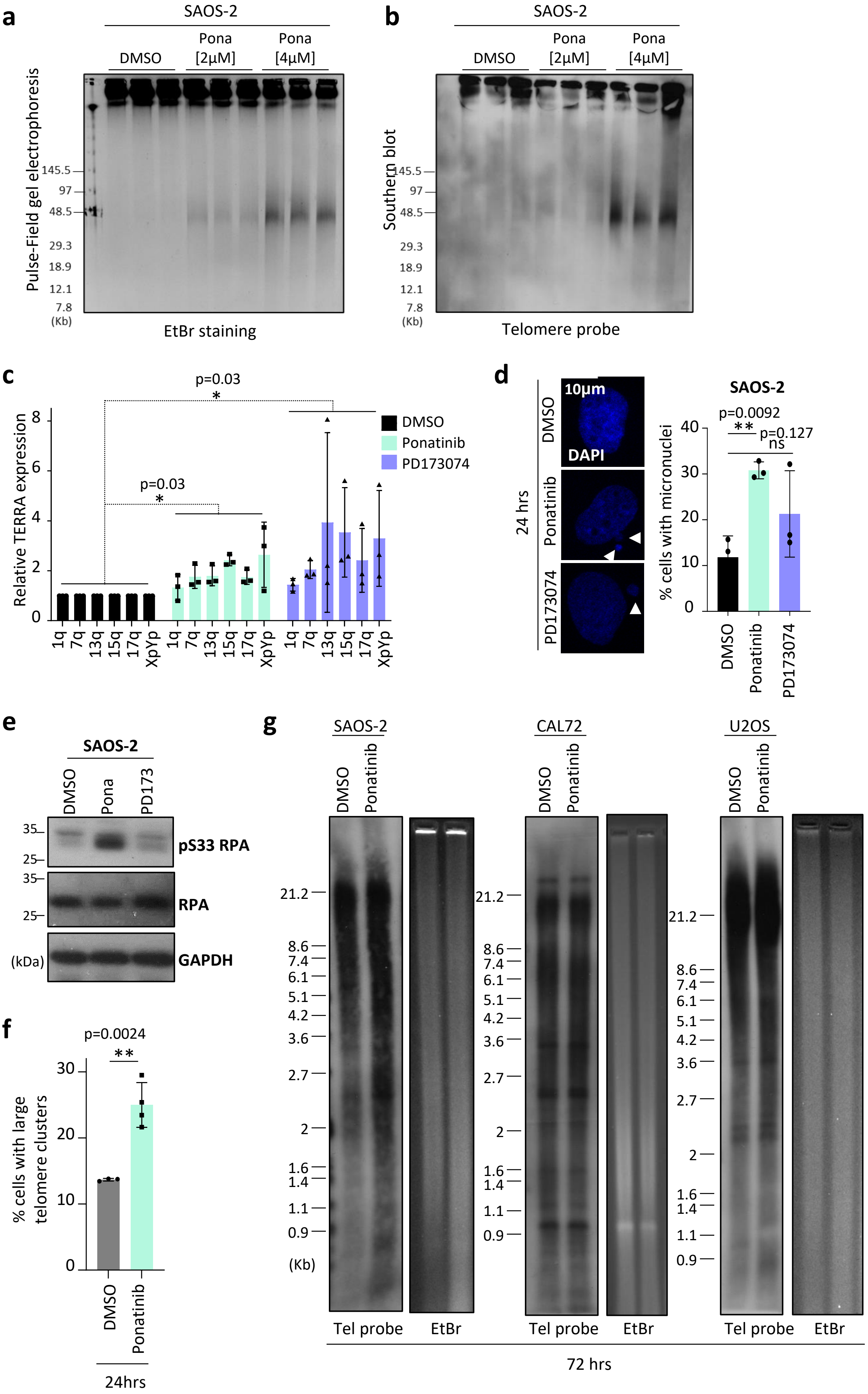

Supplementary Figure 4:

a) Detection of double-strand DNA breaks by pulse-field gel electrophoresis (PFGE) analysis of cells embedded in agarose plugs, after treatment with ponatinib for 24 hours. Each lane represents a biologically independent replicate. (n=3 for DMSO, ponatinib 2 μM and 4 μM).

b) Southern blot of gel in (a) to detect telomeric DNA fragments. Each lane represents a biologically independent replicate. (n=3 for DMSO, ponatinib 2 μM and 4 μM).

c) Chromosome specific TERRA expression levels in SAOS-2 cells treated with either ponatinib (250 nM) or PD173074 (500 nM) for 72 hours. Values represent mean ± SD of three biological replicates. (*p<0.05, as determined by two-tailed Wilcoxon test).

d) Micronuclei prevalence in SAOS-2 cells after 24 hours of treatment. Values are mean ± SD of three independent experiments. (Number of analysed cells= 297, 341 and 259 for DMSO, ponatinib and PD173074, respectively) (**p<0.01, ns=not significant, determined by two-tailed t-test).

e) Western blot showing levels of phosphorylated RPA at Serine 33 (pS33 RPA) and total levels of RPA in SAOS-2 cells treated with either ponatinib (250 nM) or PD173074 (500 nM) for 24 hours. (GAPDH=loading control; n=1).

f) Quantification of cells with at least one large telomere cluster in SAOS-2 cells treated with ponatinib for 24 hours. Values are mean ± SD. (**p<0.01, as determined by two-tailed unpaired t-test; total number of analysed cells= 380 (DMSO) and 571 (ponatinib)).

g) Telomere restriction fragment (TRF) analysis of cells treated with either ponatinib (250 nM for SAOS-2 and U2OS; 125 nM for CAL72) or PD173074 (500 nM) for 72 hours. TRF analysis was reproduced two more times for SAOS-2 (n=3), one additional time for CAL72 (n=2) and three more times for U2OS (n=4).

Source data are provided as a Source Data file.

Supplementary Figure 5

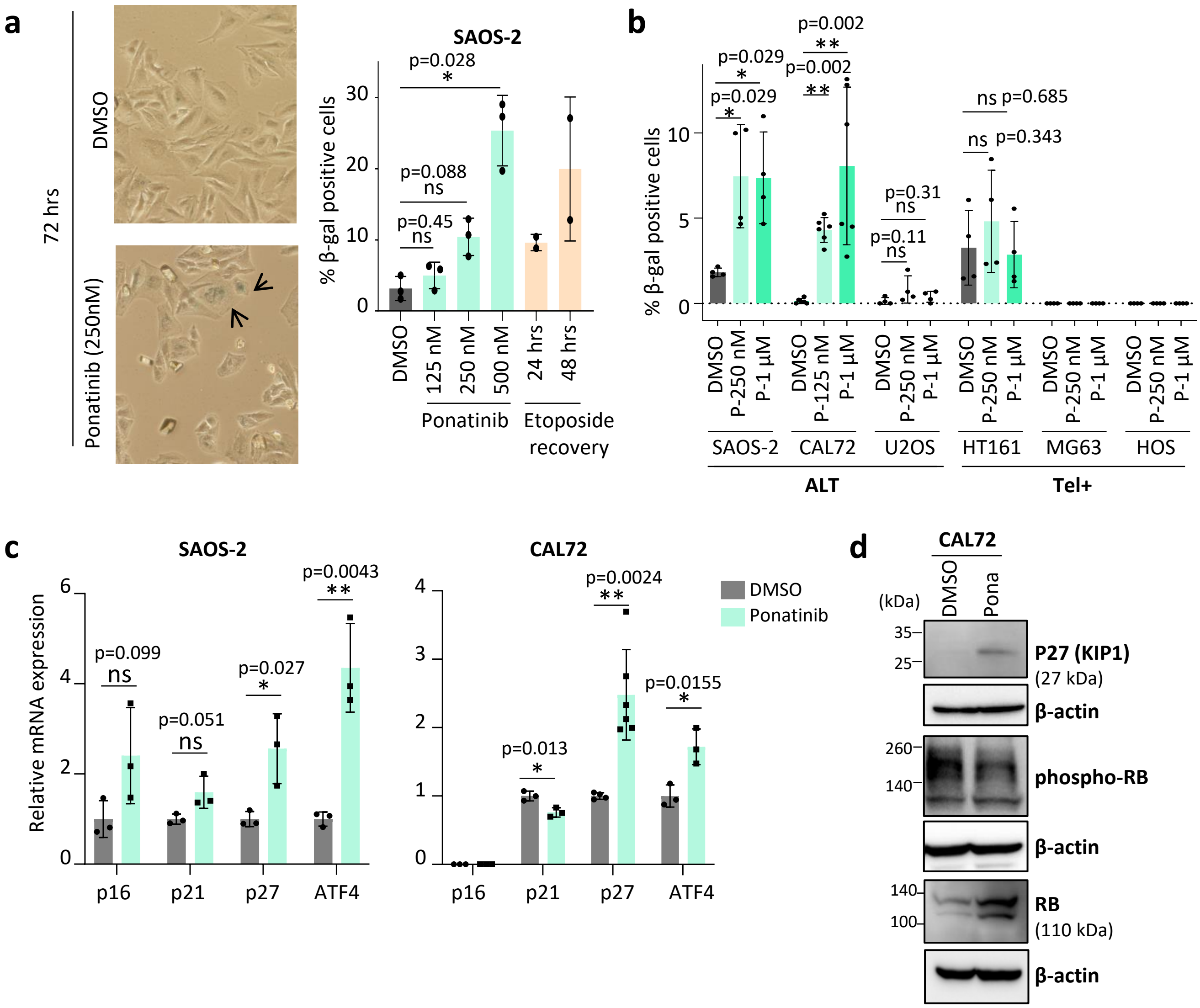

Supplementary Figure 5:

a) Senescence detection by β-galactosidase staining in SAOS-2 cells treated with different concentrations of ponatinib for 72 hours. Cells treated with etoposide (10 µM) for 24 hours and allowed to recover for 24 or 48 hours serve as a positive control. The graph shows mean percentage ± SD of β-galactosidase-positive cells from at least two independent experiments. Total analysed number of cells = 5726 for DMSO, 5586 for pona at 125 nM, 4679 for pona at 250 nM, 3702 for pona at 500 nM, 2407 for Etoposide 24 hrs and 1425 for Etoposide 48 hrs. (*$p<0.05$, ns= not significant, as determined by two-tailed t-test).

b) β-galactosidase-positive cells in different cell lines after treatment with 125 or 250 nM of ponatinib for 72 hours or with 1 µM for 24 hours and recovered for 48 hours before staining. Values are mean ± SD of at least two experiments. Total number of analysed cells is (SAOS-2: 2513, 1624, 1203), (CAL72: 6577, 3570, 1540), (U2OS: 3415, 2797, 2237), (HT161: 2458, 2751, 2975), (MG63: 3368, 2290, 2082), (HOS: > 2000) for DMSO, P-250 nM and P- 1µM respectively for each cell line. (*$p<0.05$, **$p<0.01$, ns = not significant, as determined by two-tailed Mann-Whitney test).

c) Relative mRNA expression in SAOS-2 and CAL72 cells treated for 72 hours with either DMSO or ponatinib (at 250 and 125 nM respectively). Values are mean ± SD of at least three independent biological replicates (N=3). (*$p<0.05$, **$p<0.01$, ns=not significant, as determined by two-tailed t-test).

d) Western blots showing levels of p27, phospho(Thr821/826)-Rb and Rb in CAL72 cells after treatment with ponatinib (125 nM) for 72 hours. β-actin serves as a loading control. Western blots for p27 and Rb have been reproduced twice (n=2).

Source data are provided as a Source Data file.

Supplementary Figure 6

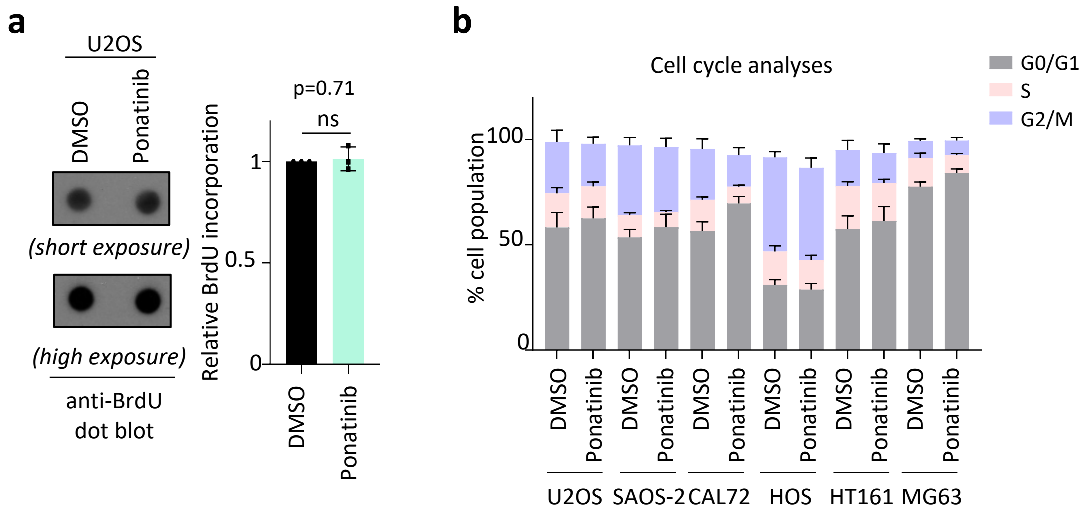

Supplementary Figure 6:

a) Total BrdU incorporation (mean ± SD) in DNA samples from Figure 3a-b using anti-BrdU antibody for dot blot detection. (ns=not significant, as determined by two-tailed t-test; n=3 biologically independent replicates).

b) Cell cycle phase distribution of cells treated with ponatinib (250 nM) for 24 hours. Values shown are mean ± SD of three independent experiments.

Source data are provided as a Source Data file.

Supplementary Figure 7

a

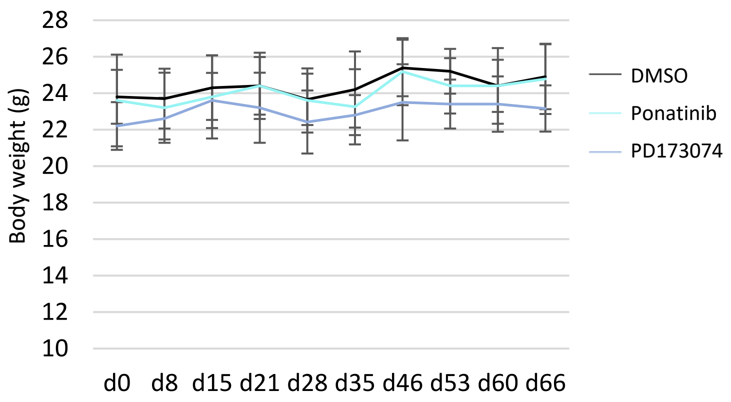

b

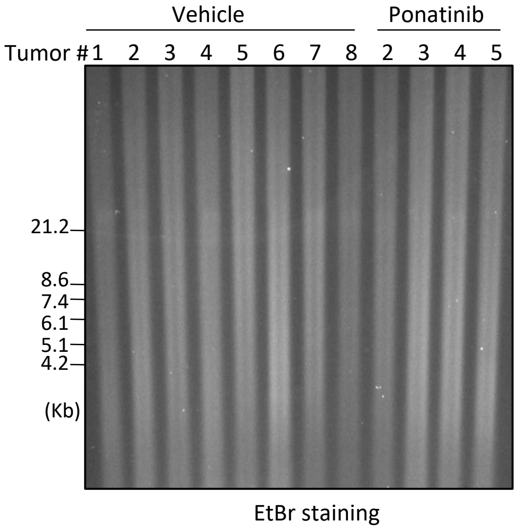

Supplementary Figure 7:

a) Body weight (mean ± SD) of mice from experiment in Figure 4a (n=10 for DMSO and 5 for ponatinib and PD173074-treated groups).

b) EtBr staining for the TRF assay in Figure 4d.

Source data are provided as a Source Data file.

Supplementary Figure 8

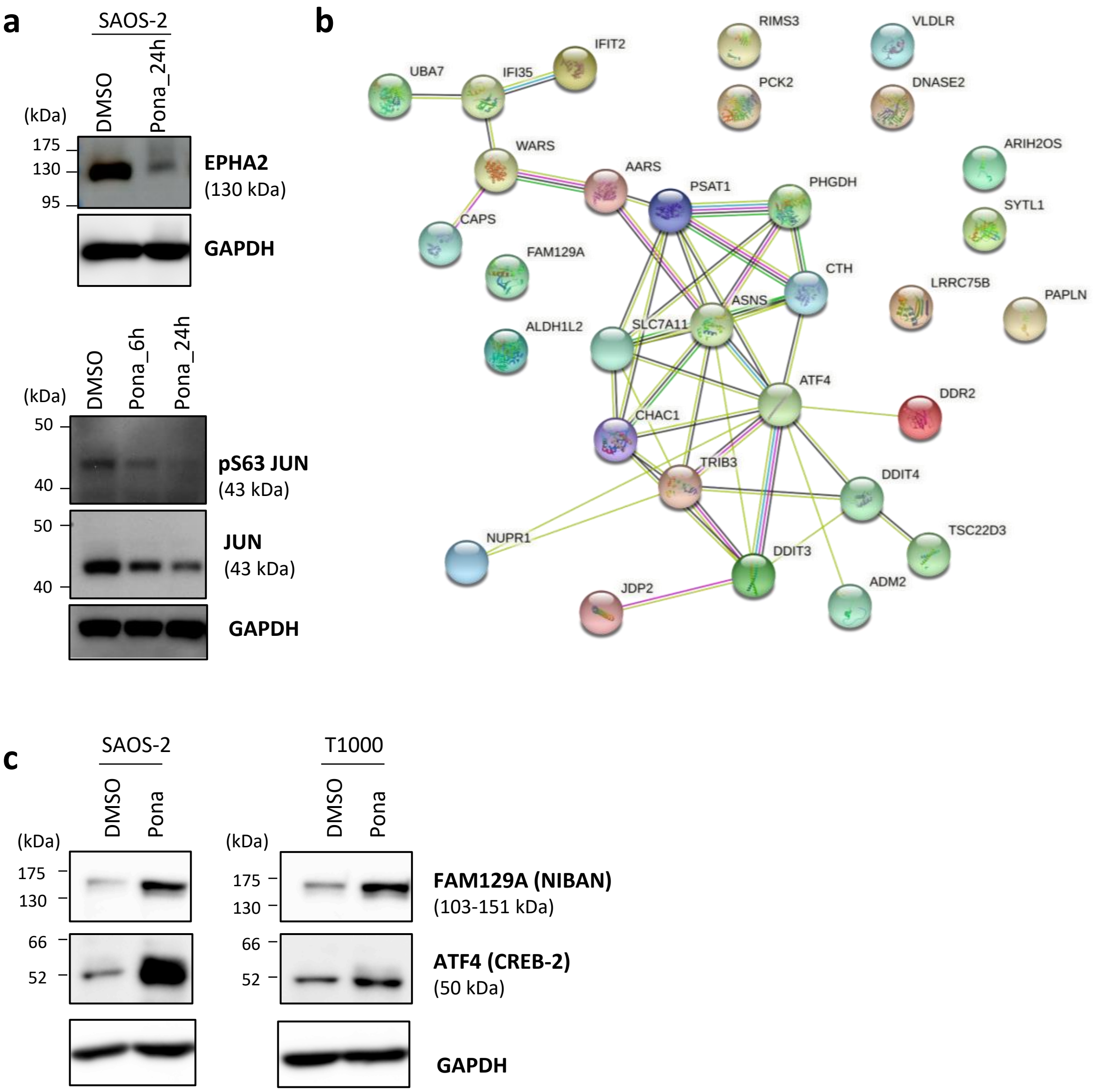

Supplementary Figure 8:

a) Western blots for EPHA2, phospho-JUN at Serine 63 (pS63 JUN) and JUN after ponatinib (250 nM) treatment. GAPDH is used as a loading control. (n=1)

b) STRING analysis (https://string-db.org/) showing functional protein networks of genes up-regulated 24 hours after ponatinib treatment (from Figure 5d). Edges between nodes represent known or predicted interactions (including gene neighborhood, gene co-occurrence or co-expression).

c) Western blots for FAM129A (Niban) and ATF4 (CREB-2) after 24 hours of ponatinib (250 nM) treatment in SAOS-2 and T1000 cells. GAPDH is a loading control. (n=1)

Source data are provided as a Source Data file.

Supplementary Figure 9

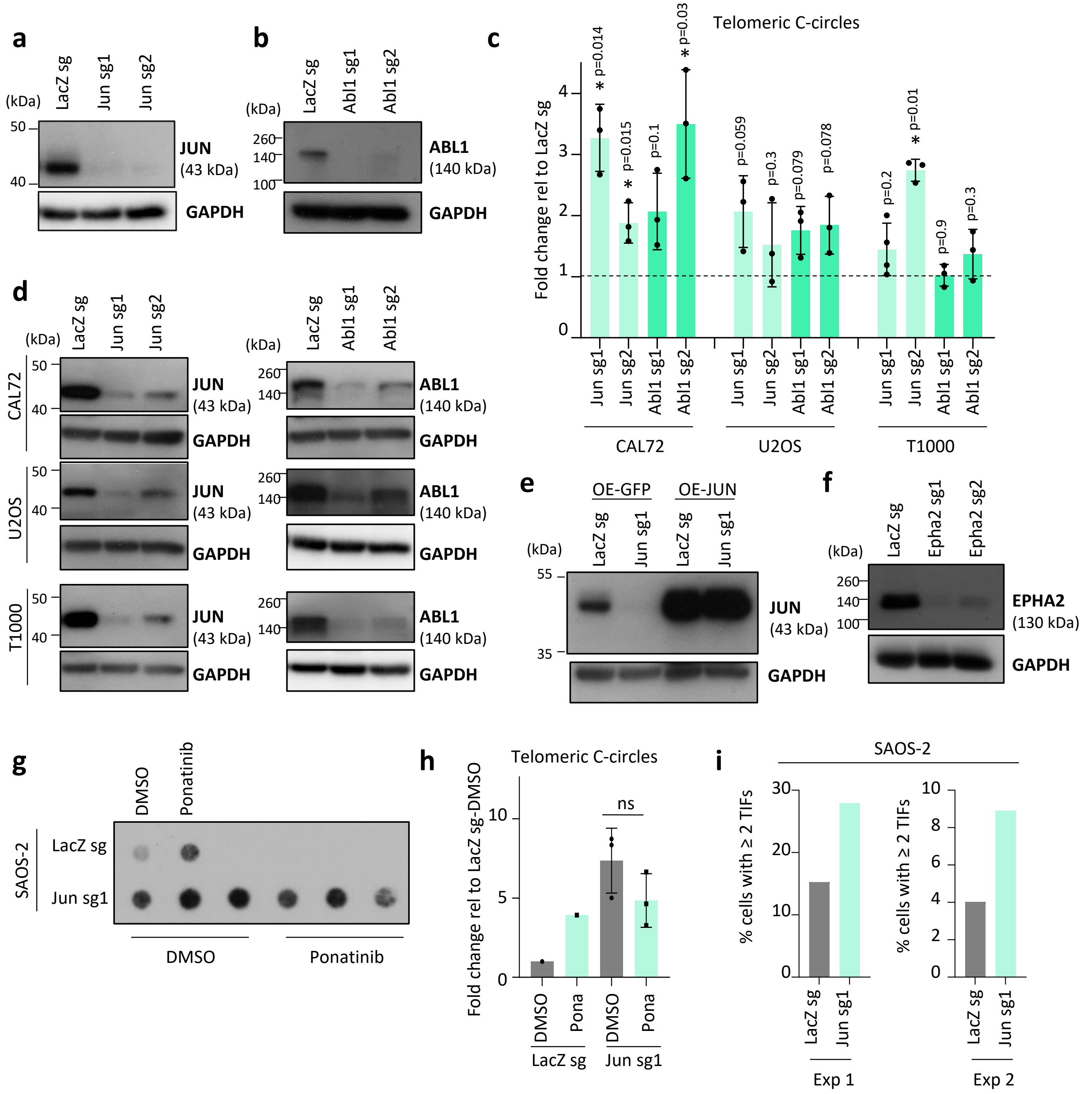

Supplementary Figure 9:

a-b) Verification of JUN and ABL1 depletion using western blot in sgRNA-transduced SAOS-2 cells. (Loading control, GAPDH).

c) Telomeric C-circle assays in CAL72, U2OS and T1000 ALT cells lacking either JUN or ABL1. Fold change of levels of telomeric C-circles is relative to those in corresponding control (LacZ sg) cells. Mean ± SD from three biological replicates is shown. (*$p<0.05$, as determined by two-tailed unpaired t-test).

d-f) Western blots for: verifying lack of JUN and ABL1 in each cell line (**d**); JUN in SAOS-2 cells used in Fig. 6d (OE= overexpression) (**e**) and EPHA2 in EPHA2 sgRNA-transduced SAOS-2 cells (**f**). GAPDH serves as a loading control.

g-h) C-circle assay on SAOS-2 cells lacking JUN after treatment with ponatinib (250 nM) for 72 hours. The graph in (h) shows the C-circle intensity quantification relative to LacZ sg-DMSO. (ns= not significant, as determined by two-tailed unpaired t-test; n=3 biologically independent replicates for Jun sg1 cells treated with DMSO or ponatinib; error bars are ± SD).

i) Telomere dysfunction-induced foci (TIFs) in SAOS-2 cells lacking JUN. Graphs are from two independent experiments (exp 1 and exp 2) on two batches of lentiviral-transduced cells. Total number of analysed cells is 118 for LacZ sg and 136 for Jun sg1 in exp 1; and 124 for LacZ sg and 101 for Jun sg1 in exp 2.

Source data are provided as a Source Data file.

Supplementary Figure 10

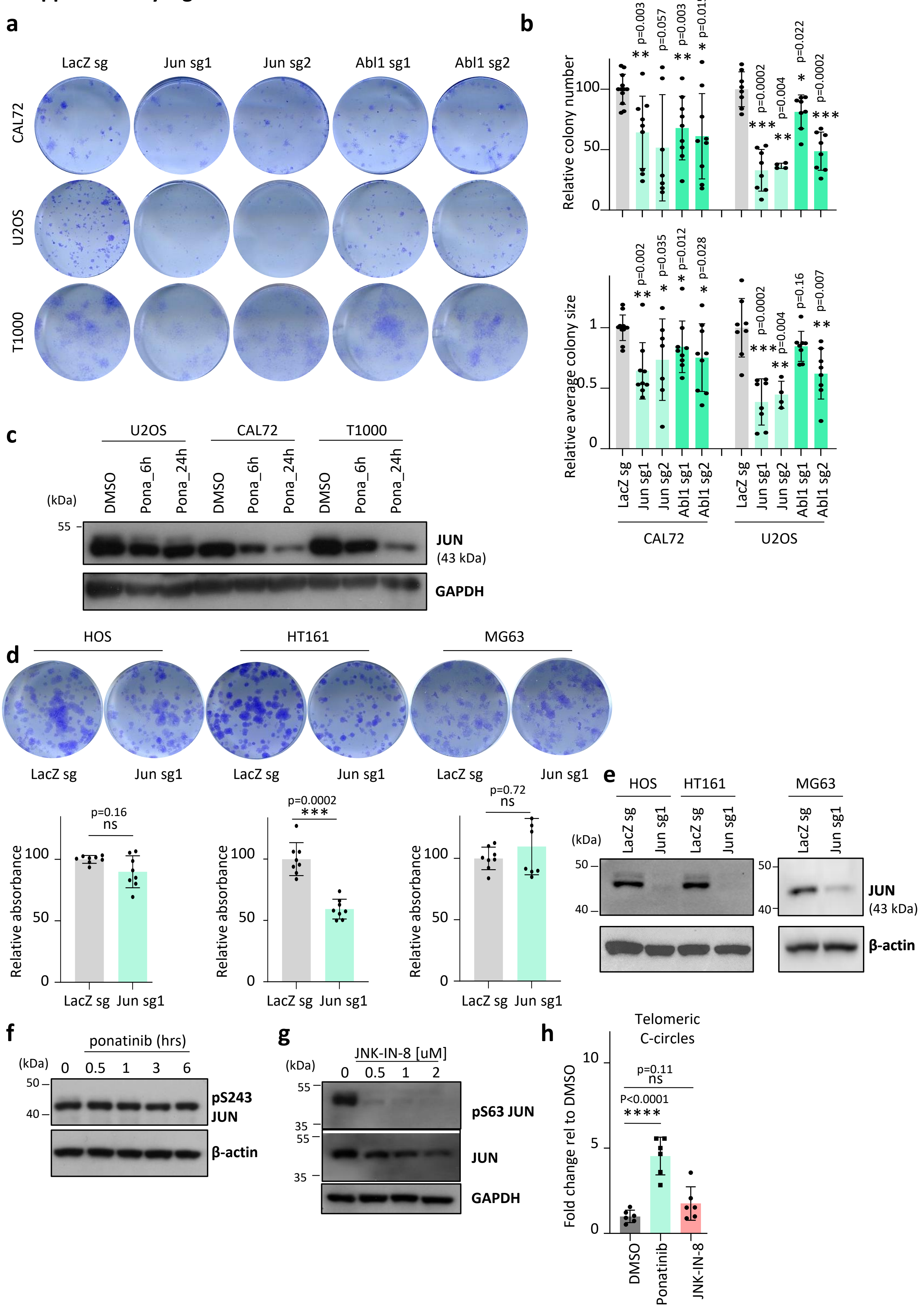

Supplementary Figure 10:

a-b) Colony formation assays either of JUN or ABL1 depleted-cells (CAL72, U2OS, T1000). Representative images of the assays are shown in (**a**). Relative colony number and size are shown (**b**). Values are average ± SD of three experiments performed each in 2-4 replicates (CAL72: n=12 for LacZ sg, n=9 for jun sg1, Abl sg1 and sg2, and n=7 for jun sg2; U2OS: n=8 for LacZ sg, Jun sg1, Abl1 sg1 and sg2, and n=4 for Jun sg2). (*p<0.05, **p<0.01, ***p<0.001, as determined by two-tailed Mann-Whitney test).

c) JUN levels detected by western blot after 6 or 24 hours of treatment with ponatinib (250 nM for U2OS and T1000, 125 nM for CAL72). (n=1)

d) Colony formation assays of telomerase-positive cells (HOS, HT161 and MG63) lacking JUN. The graphs show relative mean absorbance ± SD of lysed colonies of 8 biological replicates from two independent experiments. (***p<0.001, ns= not significant, as determined by two-tailed Mann-Whitney test).

e) Western blots showing JUN depletion in cell lines used in (d).

f-g) Western blot for: pS243 JUN in SAOS-2 cells treated with 250 nM ponatinib at different time points (**f**) (β-actin=loading control); pS63 JUN and JUN in cells treated (6 hours) with different concentrations of JNK inhibitor (JNK-IN-8) (**g**). GAPDH is used as sample processing control. (n=1)

h) Levels of telomeric C-circles in SAOS-2 cells treated for 72 hours with either ponatinib (250 nM) or JNK-IN-8 (2 µM). (****p<0.0001, ns= not significant as determined two-tailed by unpaired t-test; n=6 biological replicates for each condition).

Source data are provided as a Source Data file.

Supplementary Figure 11

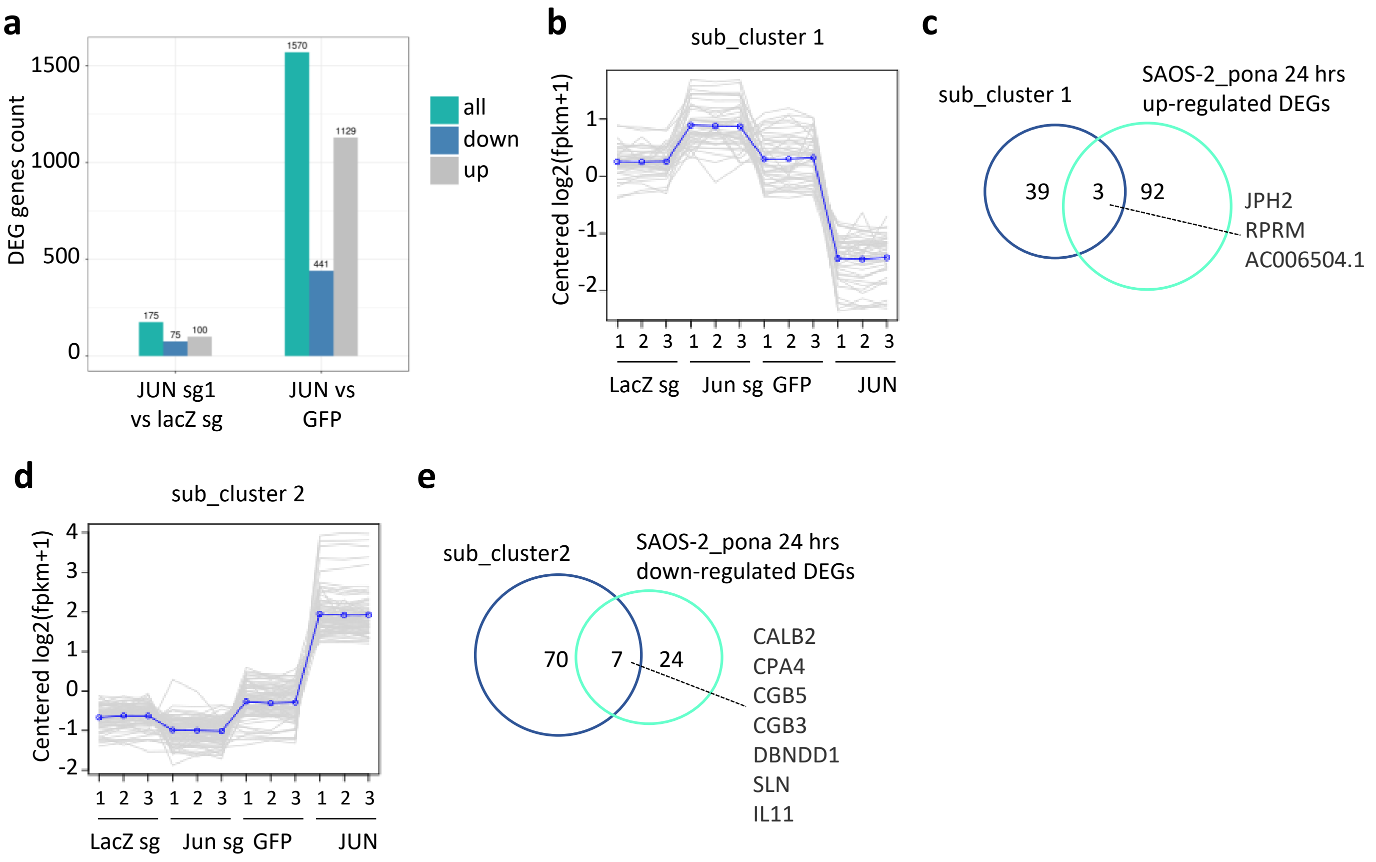

Supplementary Figure 11:

a) Count of differentially expressed genes (DEG) identified by RNA-seq analysis of SAOS-2 cells that either lack (Jun sg1 vs lacZ sg) or overexpress JUN (JUN vs GFP).

b, d) Sub-cluster of genes that display opposing expression patterns in RNA-seq comparisons.

c, e) Venn diagrams of DEGs overlapping between either sub-clusters 1 or 2 and DEGs in SAOS-2 cells treated with ponatinib for 24 hours.

Supplementary Figure 12

a

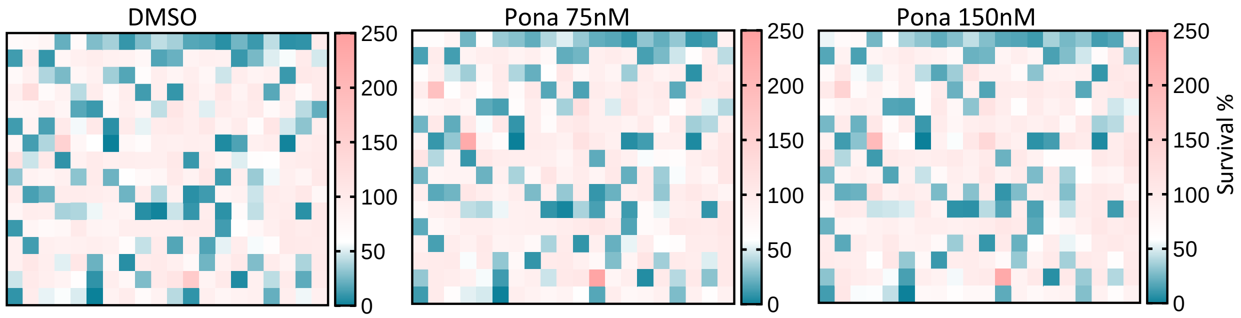

b

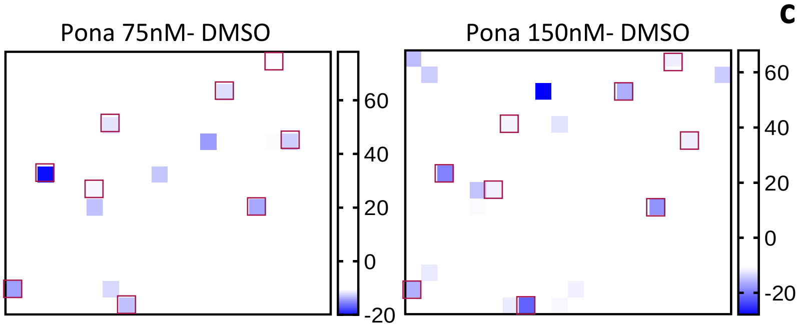

c

Compound	Targets
Triciribine	DNA synthesis, AKT
KU-60019	ATM, DNA-PKcs
Nelarabine/Arranon	DNA synthesis
Rapamycin/Sirolimus	mTOR
Dexamethasone acetate	glucocorticoid receptor agonist
CYC116	Aurora A and B
Dacomitinib/PF299804	ERBB kinases
LY2603618/IC-83	Chk1
Afatinib/BIBW2992	EGFR family

d

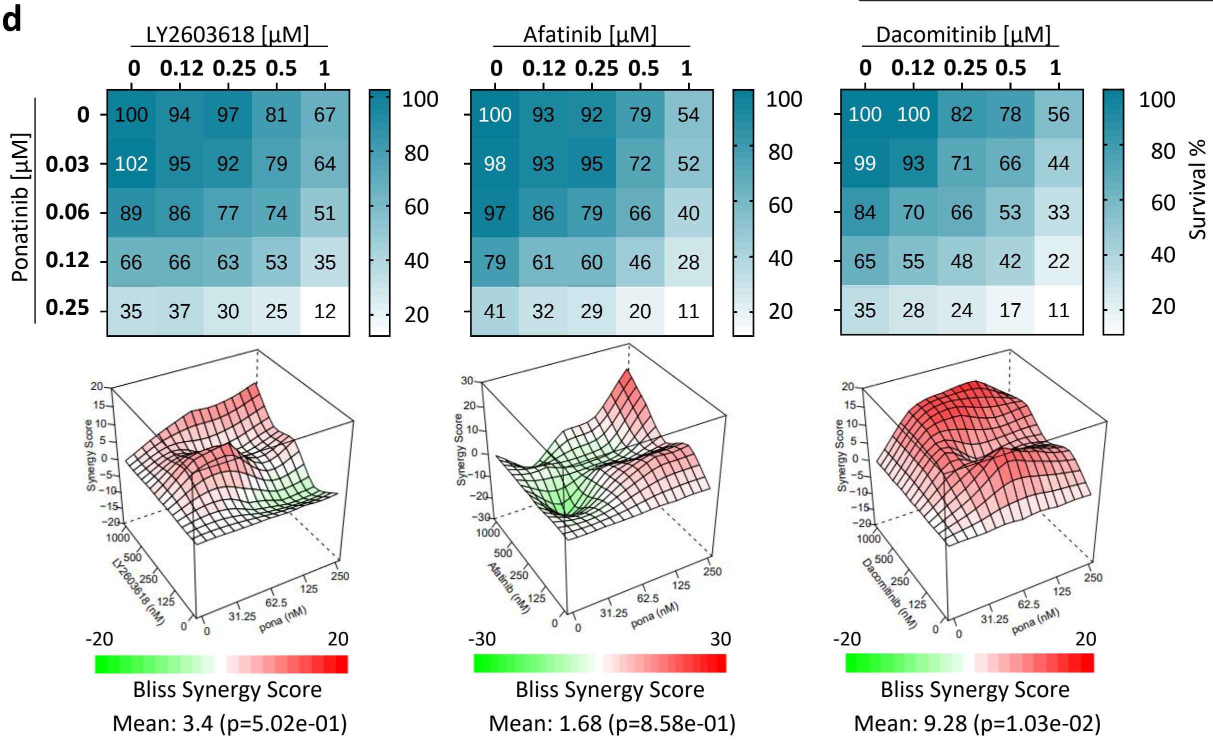

e

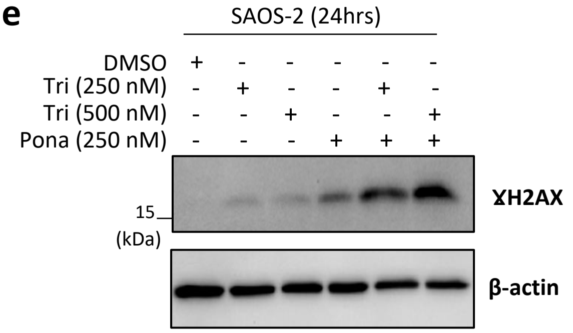

Supplementary Figure 12:

a-c) Drug library screening using SAOS-2 cells. Each heatmap shows percentage of viability of cells treated with different anti-cancer compounds relative to control cells. In addition, either DMSO (control) or ponatinib (at either 75 nM or 150 nM) were added to the whole plate. Values represent average of triplicates (**a**). Differential viability between ponatinib- or DMSO-treated plates is shown in (**b**). Only wells with at least 10% of differential survival are shown (blue) and wells identified in both screens are highlighted with red borders and listed together with their main targets in (**c**).

d) Heatmaps of viability assays of SAOS-2 cells treated with combinations of ponatinib and either LY2603618, afatinib or dacomitinib. Percentages of survival relative to DMSO-treated cells are shown (mean of two experiments performed in duplicates). Bliss synergy scores are shown in lower panels. Scores > 10 indicates synergy between the two drugs.

e) Western blot for γH2AX on SAOS-2 cells treated for 24 hours with triciribine or ponatinib or combinations of both. (n=1)

Source data are provided as a Source Data file.

Supplementary Figure 13

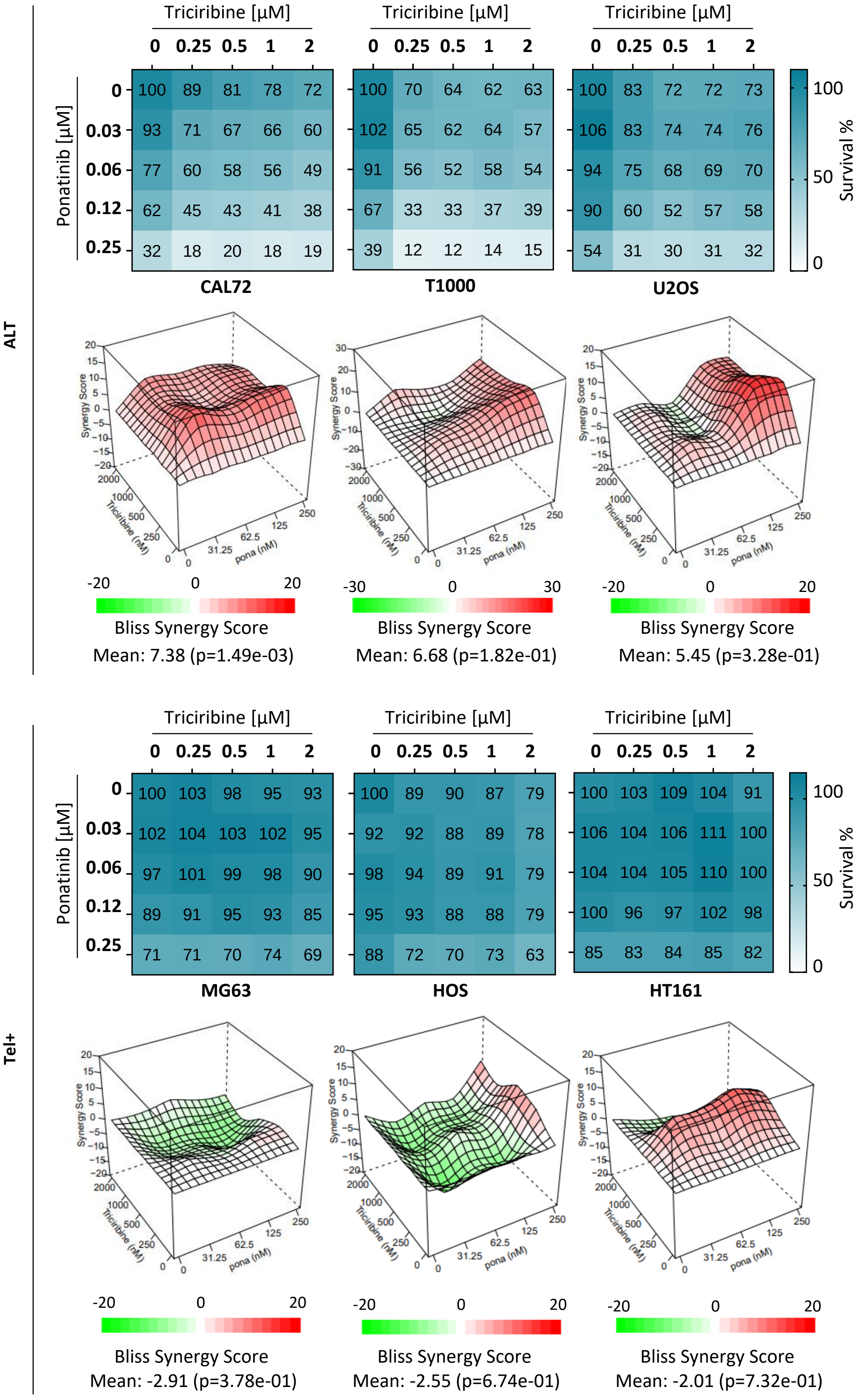

Supplementary Figure 13:

Heatmaps of viability assays of ALT or telomerase-positive (Tel+) cells treated with combinations of ponatinib and triciribine. Percentages of survival relative to DMSO-treated cells are shown (mean of three experiments performed at least in duplicates). Bliss synergy scores are shown in lower panels. Scores > 10 indicates synergy between the two drugs.

Source data are provided as a Source Data file.

Supplementary Figure 14

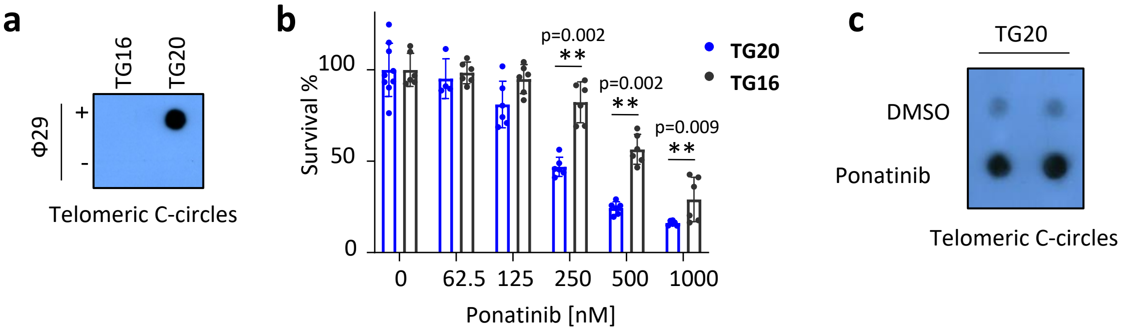

Supplementary Figure 14:

a) Telomeric C-circle levels in glioma stem-like cell line TG16 (telomerase-positive) and TG20 (ALT).

b) Viability assays showing average survival fraction ± SD of either TG16 or TG20 cells treated with ponatinib for 6 days. Values are from 4 or 6 biological replicates from two independent experiments (**$p<0.01$, as determined by two-tailed Mann-Whitney test).

c) Telomeric C-circle assay in TG20 cells treated for with ponatinib (250 nM) 72 hours.

Source data are provided as a Source Data file.

Supplementary Methods

Cell culture

Osteosarcoma (OS) cell lines (ALT-positive: SAOS-2, U2OS, CAL72 and telomerase-positive: HOS, HT161, and MG63), HEK293T, IMR90, SW26 and SW39 cells were grown in DMEM (Gibco) while liposarcoma (LPS) cell lines (T1000 (ALT) and T778 (Tel+)) were cultured in RPMI (Gibco). Media was supplemented with 10% Fetal Bovine Serum (Gibco), 100 U/mL Penicillin and 100 µg/mL Streptomycin (Gibco). All cell lines were maintained in a 37°C humidified incubator with 5% CO_2. Cell lines were mycoplasma free. CAL72, HOS, MG63, SAOS-2, U2OS and T778 cell lines were authenticated by short tandem repeat (STR) profiling in October 2020.

Glioma stem cells culture

Glioma stem-like cells (GSCs) TG20 (ALT) and TG16 (Tel+) were grown as neurospheres in serum-free DMEM/F12 supplemented with B27 minus Vitamin A (Gibco), heparin (Stem Cell Technologies), and human recombinant epidermal growth factor (EGF) and basic fibroblast growth factor (FGF2) (Stem Cell Technologies) each at 20 ng/mL. For adherent cultures, biocoat™ collagen I-coated plates (Corning) were used.

Cell viability Assay

Cells were plated at 2000 cells/well in a 96-well plate and treated the next day with corresponding drugs for 72 hours, prior to the addition of a Thiazolyl Blue Tetrazolium Bromide (MTT) reagent (Scientific Resources, Alfa Aesar) to stain the cells. The stained cells were afterwards lysed by adding detergent reagent or dimethyl sulfoxide (DMSO). Absorbance was measured at 570 nm using a TecanInfinite® M200 PRO plate reader (Tecan).

Clonogenic assays

Around 100 or 250 cells per well were seeded in 12-well or 24-well plates. After 24 hours, cells were treated with drugs and incubated for about 10-15 days. Colonies were fixed with methanol for 10 minutes and stained with 0.5% (w/v) crystal violet (Sigma-Aldrich) and methanol solution for 20

minutes. The plates were then rinsed with water and dried overnight at room temperature. Either total colony area or colony size and number were counted and calculated using FIJI software on scanned images of the wells. In Supplementary Fig. 1e-f, colonies were lysed by adding a solution of 10% acetic acid (in H2O), shaking for 15 minutes and reading the absorbance at 590 nm.

Telomere probe DIG-labelling

$(CCCTAA)_4$ oligonucleotides were purchased from IDT. 50 pmol of probe were labelled with digoxigenin (DIG) at their 3'end using 100 U of terminal transferase (New England Biolabs) and 0.05 mM of DIG-ddUTP (Roche). Reactions were incubated at 37°C for 30 min then cooled on ice. The reactions were stopped by adding EDTA.

Senescence-associated β-Galactosidase Assay

β-Galactosidase staining was performed according to the instructions of the Senescence β-Galactosidase Staining Kit (Cell Signaling). Briefly, cultured adherent cells were fixed with the fixative solution for 15 minutes and stained with the β-Galactosidase staining solution for 24-48 hours at 37°C. The presence of senescent cells noted by a blue colour was checked under an Olympus TH4-200 microscope (20-40X magnification) and quantified.

Cell cycle analysis by flow cytometry

4-5 x 10^5 cells were seeded into 10cm dishes and treated with ponatinib for 24 hours. Cells were then collected and fixed with 70% ice cold ethanol. The cells were washed, resuspended with a staining solution (50 µg/mL propidium iodide (Life Technologies), and 10 µg/mL RNAse (Thermofisher Scientific) in PBS), incubated at 37°C for 30 minutes, and then overnight at 4°C before analysis by flow cytometry. Cells were gated by forward scatter (FSC) and side scatter (SSC) to separate debris, and further gated by FSC-height (FSC-H) and FSC-area (FSC-A) to exclude doublets. Propidium iodide was used to estimate percentage of cells in each cell cycle phase.

Drugs

Ponatinib (HY-12047), PD173074 (HY-10321), Hydroxyurea (HY-B0313), JNK-IN-8 (HY-13319), Triciribine (HY-15457), KU-60019 (HY-12061), cediranib (HY-10205), R935788 (R788 disodium) (HY-13038), YM201636 (HY-13228), LY2603618 (HY-14720), afatinib (HY-10261), dacomitinib (HY-13272), Ro-3306 (HY-12529) were purchased from MedchemExpress. Dovitinib (S1018) was purchased from Selleckchem, and etoposide (E1383) from Sigma-Aldrich.

Viral production and cells transduction

Lentiviral particles were produced by transfecting HEK293T cells with 0.25 μg of gag/pol (pMDLg/pRRE), Rev (pRSV-Rev), and VSV-G (pMD2.G) plasmids each (Addgene), and the plasmid of interest, using PEI 25K™ transfection reagent (Polysciences). After 72 hours of transfection, the supernatant was collected, filtered through a 0.45μm syringe filter (Pall Corporation) and used to infect recipient cells. Media was replaced after 24 hours, and antibiotic selection (e.g., 1 μg/mL puromycin (Gibco)) was added at 48 hours.

Plug assays

Cells were collected by trypsinization, embedded in 2% low melting agarose (Sigma) and casted in plugs using CHEF disposable plug molds (Bio-Rad). Solidified plugs were then incubated overnight at 50°C in lysis buffer (EDTA (100 mM), sodium deoxycholate (0.2 %), sodium lauroyl sarcosine (1%)) and proteinase K (1 mg/mL). Plugs were washed in TE buffer 4 times for 1 hour each and inserted in 1% megabase agarose gel (Bio-Rad). Pulse-field gel electrophoresis was then performed in a CHEF-DR II apparatus (Bio-Rad) at 4 V/cm, for 14 hours, with 5-30s switch time. Gels were stained using ethidium bromide (Bio-Rad) and imaged with a Quantum ST5 imager (Vilber Lourmat). Southern blotting to detect telomeric DNA was subsequently done according to the TELOTAGGG Telomere Length Assay kit instructions.

Plasmid construction

CRISPR-Cas9 plasmids:

CRISPR-Cas9 plasmids were cloned as previously described [1]. LentiCRISPRv2puro was digested with BsmBI enzyme (New England Biolabs), and the digested plasmid was gel extracted (QIAquick gel extraction kit, Qiagen). Forward and reverse oligonucleotides specific for each gene (Supplementary Data 5) were designed using the broad institute portal [2, 3] and purchased from Integrated DNA Technologies (IDT). Oligonucleotides were phosphorylated and annealed (incubation for 1 hour at 37°C with T4 PNK (New England Biolabs) and then 5 minutes at 95°C and cooled down until room temperature). Annealed oligonucleotides were ligated with the purified digested plasmid using T4 ligase enzyme (New England Biolabs) overnight at 16°C. Following ligation, bacterial transformation into competent Stlb3 bacteria was performed. Colonies were picked and inoculated into 5mL of LB broth supplemented with ampicillin in a shaking incubator at 37°C. After 16 hours, plasmids were extracted from the mini cultures using the FavorPrep Plasmid Extraction Mini Kit (Favorgen) and sequenced to verify the oligonucleotides insertion.

Overexpression plasmids:

JUN and ABL in pDONR223 vectors were purchased from Addgene and used to generate overexpression vectors by Gateway cloning, using the Gateway® LR Clonase® II enzyme mix (Thermofisher Scientific), according to the manufacturer's instructions. Destination vectors were pLEX_307 (Addgene; for lentiviral expression) or Gateway™ pcDNA™-DEST47 Vector (Thermofisher Scientific; for transient transfection).

Site-directed mutagenesis:

Single point mutations were introduced in plasmids using Q5® Site-Directed Mutagenesis Kit (New England BioLabs), according to the manual. For sgRNA resistant plasmids, silent mutations were introduced in the NGG motif. For the phosphomutant plasmids, serines (S) and threonines (T) were replaced by alanines (A), while tyrosines (Y) were replaced by phenylalanine (F). Primers (5'→ 3')

used for site-directed mutagenesis (Supplementary Data 5) are designed using NEBaseChanger (New England BioLabs).

A list of the used plasmids is provided in Supplementary Data 5.

Western blot

Total proteins were extracted using either RIPA buffer or M-PER^{TM} (ThermoFisher Scientific) supplemented with cOmplete™ EDTA-free Protease Inhibitor Cocktail Tablets (Sigma-Aldrich) and phosphatase inhibitor PhoSTOP tablets (Sigma-Aldrich). Protein concentration was estimated using the Pierce™ BCA Protein Assay Kit (ThermoFisher Scientific). SDS loading buffer 6X was added to 10-20 µg of proteins and the samples were denatured at 95°C for 10 minutes before loading them onto a 10 or 15% SDS-PAGE gel. Electrophoresis was performed at 100-160 Volts for 1.5 hours, then proteins were transferred onto a PVDF membrane (Bio-Rad) at 40 Volts and 4°C overnight. The membrane was blocked with 5% blotting-grade blocker (Bio-Rad) or bovine serum albumin (BSA, Sigma-Aldrich) in Tris-buffered saline + 0.1% Tween20 (TBST) for 1 hour, prior to incubation with primary antibodies (Supplementary Data 5) in 3% blotting-grade blocker or BSA in Tris-buffered saline (TBS) for 1 hour at room temperature or overnight at 4°C. The membranes were washed and incubated with HRP-coupled secondary antibodies before detection using SuperSignal™ West Femto Maximum Sensitivity Substrate (ThermoFisher Scientific). When needed, membranes were stripped using a Stripping Buffer (0.2M glycine, 0.1% (w/v) sodium dodecyl sulfate (SDS), 1% Tween20, pH 2.2) for 30 minutes and re-blocked before re-probing. Uncropped scans of the blots are provided in the source data files.

Immunofluorescence (IF) staining

IF staining was performed on chamber slides. Cells were fixed with 4% formaldehyde for 10 minutes and permeabilized with PBS and 0.5% Triton-X for 15 minutes. Slides were then blocked with 3% BSA and 0.1% Triton-X in PBS for one hour, followed by an hour incubation at 37°C with primary antibodies (Supplementary Data 5) diluted in PBS+1% BSA. After washes with PBS, fluorescent-

labelled secondary antibodies were then added for an hour and incubated at 37°C. Slides were mounted with Prolong gold DAPI (Invitrogen) before sealing the slide with a coverslip. Images were captured using Nikon A1R, Zeiss LSM 880 confocal microscopes or Zeiss Axio Z1 system and analysed using Fiji/ImageJ.

Quantitative Real Time-PCR (qRT-PCR)

RNA was extracted using the ReliaPrep™ RNA Miniprep Systems kit (Promega) according to the manufacturer's instructions, and cDNA was synthesized by the EvoScript Universal cDNA Master kit (Roche). For experiment in Supplementary Figure 5c, RNA was extracted using EZ-10 DNAaway RNA mini-prep kit (Biobasic) according to the manufacturer's instructions, and cDNA was synthesized using the SuperScript™ IV First-Strand Synthesis System (ThermoFisher Scientific). Diluted cDNA was then mixed with desired primers and Precision Fast qPCR MasterMix - Low Rox-SYBR, and reactions performed in 7500 Fast Real-time cycler (Applied Biosystems).

qRT-PCR for TERRA detection

Total RNA was extracted using the ReliaPrep™ RNA Cell Miniprep Systems kit (Promega) according to the manufacturer's instructions. TERRA detection by qPCR was performed as previously described [4]. Briefly, cDNA synthesis was performed on extracted RNA using TERRA-specific RT primer (5'-CCCTAACCCTAACCCTAACCCTAACCCTAA-3') and GAPDH-specific RT primer (5'-GCCCAATACGACCAAATCC-3') and SuperScript™ IV First-Strand Synthesis System (ThermoFisher Scientific). Quantitative real-time PCR amplification was performed using chromosome-specific primers [4] (Supplementary Data 5) and Precision Fast qPCR MasterMix - Low Rox- SYBR in 7500 Fast Real-time cycler (Applied Biosystems).

Preparation of metaphase spreads

Cells were treated for 4-5 hours with 50 ng/mL of nocodazole (Sigma-Aldrich). Cells were then collected by trypsinization and resuspended with a KCl hypotonic solution (0.075 M KCl) and incubated at 37°C for 20 minutes. Drops of fixative solution (glacial acetic acid/methanol 1:3) were

added before pelleting the cells and re-fixing them with the fixative solution at 4°C overnight. The next day, cells were pelleted and resuspended with 500 μL-1 mL of fixative solution. 100 μL of the cell solution were dropped onto pre-warmed (50°C) Superfrost Plus glass slides (Fisher scientific). The preparations were then dried and stored at -20°C until use.

Telomeric FISH on metaphase spreads

Slides were hydrated with PBS and fixed with 4% formaldehyde for 2 minutes and subjected to pepsin (Sigma-Aldrich) digestion (0.5 mg/mL in 0.1 M HCl) for 4 minutes at 37°C. Slides were washed with PBS and re-fixed for 5 minutes. Serial ethanol washes (50%, 80% and 100%) were then performed prior to incubation with telomere FISH probes and denaturation for 5 minutes at 80°C. The slides were then kept in the dark to hybridize for 2 hours at room temperature. Subsequently, series of washes with wash buffer 1 and wash buffer 2 (refer to FISH-IF protocol) were performed before mounting with Prolong Gold antifade containing DAPI (Invitrogen).

Gene expression analyses by RNA-sequencing

For gene expression analysis after ponatinib treatment, total RNA was extracted using RNeasy Mini Kit (Qiagen). Poly-A enriched mRNAs were used for library preparation using TruSeq RNA Sample Preparation Kit (Illumina) according to the manufacturer's manual. Sequencing was performed on HiSeq 4000 and 100 bp paired-end reads were pseudo-aligned and quantified to Gencode [5] (GRCh38.p13) transcripts using the Kallisto program [6]. Transcript level fragment counts were summarized to gene level using the TxImport Bioconductor package [7]. Differential gene expression analysis was performed using the DESeq2 package [8] with lfc Threshold argument set to 0.1. Gene expression was quantified in FPKM units for every gene using the DESeq2::fpkm function. The p-values were obtained using the default two-sided Wald test and then adjusted for multiple testing using the Benjamini-Hochberg method. Gene set enrichment analysis (GSEA) [9] was performed on all active genes (mean FPKM > 0.5) while gene ontology analyses were done using the Goseq Bioconductor package [10].

For gene expression analysis in SAOS-2 cells lacking or overexpression JUN, total RNA was extracted using the ReliaPrep™ RNA Cell Miniprep Systems kit (Promega) and mRNAs were selected using poly-T oligo-attached magnetic beads. After fragmentation, the first strand cDNA was synthesized using random hexamer primers, followed by the second strand cDNA synthesis using dUTP for directional library. Quantified libraries were sequenced on Novaseq 6000 platform (Illumina). Paired-end reads were aligned to the reference genome using Hisat2 v2.0.5 [11]. FeatureCounts v1.5.0-p3 [12] was used to count the reads numbers mapped to each gene and subsequently, FPKM values were calculated for every gene. R package hclust was used for clustering genes while the cutree R package was used to generate subclusters. Differential expression analysis was performed using the DESeq2 R package (1.20.0). Genes with an adjusted P-value <= 0.05 found by DESeq2 were assigned as differentially expressed.

Receptor tyrosine kinase (RTK) array

SAOS-2 or T1000 cells were treated with either DMSO or ponatinib for 6 hours. 300 μg of protein extracts were used to perform kinase tyrosine phosphorylation profiling using Proteome Profiler Human Phospho-RTK Array Kit (R&D System, ARY001B) according to the manual's instructions.

Stable isotope labeling using amino acids in cell culture (SILAC)

For SILAC labelling, cells were incubated in DMEM or RPMI (-Arg, -Lys) medium containing 10% dialyzed fetal bovine serum (PAN-Biotech) supplemented with 84 mg/l $^{13}C_6{}^{15}N_4$ L-arginine and 50 mg/l $^{13}C_6{}^{15}N_2$ L-lysine (Cambridge Isotope) or the corresponding non-labelled amino acids. Successful SILAC incorporation with rates of > 98% were verified by in-gel trypsin digestion and MS analysis of 'heavy' input samples.

SILAC-based quantitative proteomics

SAOS-2 SILAC-labelled cells were treated with DMSO or ponatinib at 250 nM for 24 hours. Proteins were extracted using RIPA buffer (ThermoFisher Scientific) supplemented with cOmplete™ EDTA-free Protease Inhibitor Cocktail Tablets (Sigma-Aldrich) and phosphatase inhibitor PhoSTOP tablets

(Sigma-Aldrich). Protein concentration was estimated using BCA Protein Assay Kit and samples were processed for mass spectrometry analysis as described below. Equal amounts of corresponding heavy or light-labelled DMSO or drug-treated extracts were mixed, and 200 µg of protein were boiled at 95°C and used for MS sample preparation.

SILAC-based quantitative phosphoproteomics

SILAC-labelled SAOS-2 cells treated with either DMSO or ponatinib at 250 nM for 24 hours were collected by scraping in cold PBS on ice. Cells were lysed in RIPA buffer supplemented with cOmplete™ EDTA-free Protease Inhibitor Cocktail and PhosSTOP phosphatase inhibitor. 5 mg of proteins were used to perform a TiO2-based phosphopeptide enrichment. Heavy or light-labelled DMSO or ponatinib-treated extracts were mixed, reduced by adding 10 µL of 1M DTT per 1 mL of protein extract (final concentration: 10 mM) and incubating at 56°C for 1 hour. Next, samples were alkylated with 55 mM iodoacetamide (Sigma) by adding 100 µL of a 550 mM stock solution per 1 mL of protein extract, followed by incubation for 45 min in the dark. Prior to in-solution digestion, samples were diluted 3:1 with 50 mM ammonium bicarbonate buffer (pH 8.0). The digestion was then performed by adding trypsin (Promega) at 1:100 ratio (w/w) at 37°C overnight (i.e., 50 µg trypsin were added to each 5 mg of protein). Peptides were desalted by adding 10 µL of trifluoroacetic acid per 1 mL of protein extract (TFA, 1% final concentration) and cleaned on SEP-PAK C18 columns (Waters). The columns were primed with 10 mL of methanol followed by one wash with 10 mL of solution B (0.5% formic acid and 80% acetonitrile) and two washes with 10 mL of solution A (0.5% formic acid). Next, the digested protein extracts were applied to the column followed by a wash with 10ml of solution A. Finally, peptides were eluted in 5ml of solution B and concentrated by speed vacuum centrifugation (Eppendorf) to 50 µL. Samples were then mixed with solution C (solution B + 4M lactic acid) to obtain a final concentration of 2 M lactic acid. 100 mg of titanium dioxide (TiO2) beads (GL Sciences) were washed with 1mL of solution D (solution B + 2M lactic acid), and added to the peptides at a 1:1 ratio (w/w, 1 mg of TiO2 beads for each 1 mg of protein digest) and incubated for 1 hour on a

rotating wheel at room temperature. For washing and elution, a single layer of C8 (3M) was packed into a 200 µL pipette tip and washed with 200 µL of solution A followed by 200 µL of solution B. Subsequently, the TiO2 mix was loaded to the C8 tips, followed by 3 washes with 200 µL of solution D and 3 washes with 200 µL of solution B. Samples were eluted twice with 100 µL of 400 mM NH_4OH. 17.5 µL of formic acid were added to the samples for neutralization before C18 stage tipping as part of the general mass spectrometry analysis workflow described below.

JUN interactome identification by label-free quantification (LFQ)

Immunoprecipitation experiments were performed using non-labelled nuclear extracts of either SAOS-2 jun sg1 cells or cells treated with DMSO or ponatinib at 250 nM for 3 hours. IP reactions were performed in quadruplicates. For each reaction, 300 µg of nuclear extracts were used and 2 µg of antibodies (sc-74543; Santa Cruz Biotechnology) were non-covalently coupled to 15 µL of Dynabeads™ protein G (Life Technologies). The bead-antibody matrixes were then washed and incubated on a rotating wheel for 2 hours at 4°C with 300 µg of extracts diluted in protein binding buffer (PBB) (150mM NaCl, 50mM Tris, 5mM $MgCl_2$, 0.25% NP40, 1mM DTT) supplemented with protease and phosphatase inhibitors. Next, the beads were washed three times with cold PBB, and proteins were eluted from the beads by heating the samples in SDS loading buffer. Samples were processed for peptide identification by mass spectrometry as described below.

Mass spectrometry analysis

Samples were separated on a 4-12% (for proteome) or 12 % (for JUN interactome) NuPAGE Bis-Tris precast gel (Thermo Fisher Scientific) for 60 min (proteome) or 10 min (JUN IP) at 170 V. The gel was fixed using the Colloidal Blue Staining Kit (Thermo Fisher Scientific). For proteome samples, each lane was divided into 8 equal fractions, while for JUN IP samples, a single fraction was processed. For in-gel digestion, samples were destained (25 mM ammonium bicarbonate; 50% ethanol), reduced in 10 mM DTT for 1h at 56°C followed by alkylation with 55mM iodoacetamide (Sigma) for 45 min in the dark. Tryptic digestion was performed with 2 µg trypsin (Promega) in 50 mM ammonium

bicarbonate buffer at 37°C overnight. Peptides were desalted on stage tips by packing two layers of C18 (3M) into a 200 µL pipette tip. The stage tips were first primed with 50 µL methanol, followed by 1 wash with 50 µL solution B (0.5% formic acid in 80% acetonitrile) and 2 washes with 50 µL solution A (0.5% formic acid). Next, the samples (proteome, JUN IP or phosphoproteome) were applied followed by 1 wash with solution A. Samples were stored on stage tips upon MS analysis for which they were eluted with 30 µL of solution B, speed vacuum concentrated to 6 µL in order to remove the acetonitrile, and diluted to 12 µL with solution A. 6 µL of the sample (i.e. 50% of the original reaction) were stored at -20C as a back-up while the other 6 µL were placed into an EASY-nLC 1200 system at 7C and 5ul were injected for nanoflow liquid chromatography coupled to a Q Exactive HF mass spectrometer (Thermo Fisher Scientific) for the proteome and phosphoproteome samples and to a timsTOF fleX (Bruker) for the JUN IP samples. For the proteome and phosphoproteome, peptides were separated on a C18-reversed phase column (25 cm long, 75 µm inner diameter) packed in-house with ReproSil-Pur C18-AQ 1.9 µm resin (Dr Maisch). The column was mounted on an Easy Flex Nano Source and temperature controlled by a column oven (Sonation) at 40°C. A 215-min gradient from 2 to 40% acetonitrile in 0.5% formic acid at a flow of 225 nl/min was used. Spray voltage was set to 2.4 kV. The Q Exactive HF was operated with a TOP20 MS/MS spectra acquisition method per MS full scan. MS scans were conducted with 60,000 at a maximum injection time of 20 ms and MS/MS scans with 15,000 resolution at a maximum injection time of 50 ms. For the JUN IP, peptides were separated on an Aurora series column (25 cm x 75 µm, 1.6 µm C18 resin) with an integrated captive spray insert (IonOpticks). The column was mounted on an Easy Flex Nano Source and temperature controlled by a column oven (Sonation) at 50°C. A 105-min gradient from 2 to 40% acetonitrile in 0.1% formic acid at a flow of 400 nl/min was used. Spray voltage was set to 1.65 kV. The timsTOF flex was operated with data-dependent acquisition (DDA) in PASEF mode with 10 PASEF ramps per topN acquisition cycle (cycle time 1.17s) and a target intensity of 10,000. Singly charged precursor ions were excluded based on their position in the m/z-ion mobility plane and precursor ions that reached the target intensity

were dynamically excluded for 24 seconds. For proteome and phosphoproteome, the raw files were processed with MaxQuant [13] version 1.5.2.8 with preset standard settings for SILAC labeled samples and the re-quantify option was activated. For the JUN IP, the raw files were processed with MaxQuant version 2.0.1.0 with LFQ quantification (without fast LFQ) using at least 2 LFQ ratio counts and the match between run option activated. Carbamidomethylation was set as fixed modification while methionine oxidation and protein N-acetylation were considered as variable modifications in addition to phospho (STY) for the phosphoproteome samples. Search results were filtered with a false discovery rate of 0.01. Known contaminants, proteins groups only identified by site, and reverse hits of the MaxQuant results were removed. For volcano plots, p-values were obtained by performing a standard two-sided t-test using the scipy.stats module in python.

Supplementary References

1. Sanjana, N.E., O. Shalem, and F. Zhang, *Improved vectors and genome-wide libraries for CRISPR screening.* Nat Methods, 2014. **11**(8): p. 783-784.
2. Doench, J.G., et al., *Optimized sgRNA design to maximize activity and minimize off-target effects of CRISPR-Cas9.* Nat Biotechnol, 2016. **34**(2): p. 184-191.
3. Sanson, K.R., et al., *Optimized libraries for CRISPR-Cas9 genetic screens with multiple modalities.* Nat Commun, 2018. **9**(1): p. 5416.
4. Feretzaki, M. and J. Lingner, *A practical qPCR approach to detect TERRA, the elusive telomeric repeat-containing RNA.* Methods, 2017. **114**: p. 39-45.
5. Frankish, A., et al., *Gencode 2021.* Nucleic Acids Res, 2021. **49**(D1): p. D916-D923.
6. Bray, N.L., et al., *Near-optimal probabilistic RNA-seq quantification.* Nat Biotechnol, 2016. **34**(5): p. 525-7.
7. Soneson, C., M.I. Love, and M.D. Robinson, *Differential analyses for RNA-seq: transcript-level estimates improve gene-level inferences.* F1000Res, 2015. **4**: p. 1521.
8. Love, M.I., W. Huber, and S. Anders, *Moderated estimation of fold change and dispersion for RNA-seq data with DESeq2.* Genome Biol, 2014. **15**(12): p. 550.
9. Subramanian, A., et al., *Gene set enrichment analysis: a knowledge-based approach for interpreting genome-wide expression profiles.* Proc Natl Acad Sci U S A, 2005. **102**(43): p. 15545-50.
10. Young, M.D., et al., *Gene ontology analysis for RNA-seq: accounting for selection bias.* Genome Biol, 2010. **11**(2): p. R14.
11. Kim, D., et al., *Graph-based genome alignment and genotyping with HISAT2 and HISAT-genotype.* Nat Biotechnol, 2019. **37**(8): p. 907-915.
12. Liao, Y., G.K. Smyth, and W. Shi, *featureCounts: an efficient general purpose program for assigning sequence reads to genomic features.* Bioinformatics, 2014. **30**(7): p. 923-30.
13. Cox, J. and M. Mann, *MaxQuant enables high peptide identification rates, individualized p.p.b.-range mass accuracies and proteome-wide protein quantification.* Nat Biotechnol, 2008. **26**(12): p. 1367-72.
