# Supplementary material for: Signalling inhibition by ponatinib disrupts productive alternative lengthening of telomeres (ALT)
Source: Nat Commun. 2023 Apr 6;14:1919. doi: 10.1038/s41467-023-37633-3 (PMC10079688; doi:10.1038/s41467-023-37633-3)
Supplement: Supplementary file 3 — Description of Additional Supplementary Files [file 41467_2023_37633_MOESM3_ESM.pdf]

Description of Additional Supplementary Files

File Name: **Supplementary Data 1**
Description: List of phosphopeptides measured by SILAC-based quantitative phosphoproteomics

File Name: **Supplementary Data 2**
Description: List of proteins identified by SILAC-based quantitative proteomics

File Name: **Supplementary Data 3**
Description: List of differentially expressed genes after ponatinib treatment

File Name: **Supplementary Data 4**
Description: List of interactors of JUN identified by label-free quantitative proteomics

File Name: **Supplementary Data 5**
Description: List of reagents (primers, gRNAs, antibodies)
