# Supplementary material for: Signalling inhibition by ponatinib disrupts productive alternative lengthening of telomeres (ALT)
Source: Nat Commun. 2023 Apr 6;14:1919. doi: 10.1038/s41467-023-37633-3 (PMC10079688; doi:10.1038/s41467-023-37633-3)
Supplement: Supplementary file 9 — Reporting Summary [file 41467_2023_37633_MOESM9_ESM.pdf]

nature portfolio

Corresponding author(s): Maya Jeitany

Last updated by author(s): Feb 27, 2023

Reporting Summary

Nature Portfolio wishes to improve the reproducibility of the work that we publish. This form provides structure for consistency and transparency in reporting. For further information on Nature Portfolio policies, see our Editorial Policies and the Editorial Policy Checklist.

Statistics

For all statistical analyses, confirm that the following items are present in the figure legend, table legend, main text, or Methods section.

n/a	Confirmed	
☐	☒	The exact sample size (*n*) for each experimental group/condition, given as a discrete number and unit of measurement
☐	☒	A statement on whether measurements were taken from distinct samples or whether the same sample was measured repeatedly
☐	☒	The statistical test(s) used AND whether they are one- or two-sided<br>*Only common tests should be described solely by name; describe more complex techniques in the Methods section.*
☐	☒	A description of all covariates tested
☒	☐	A description of any assumptions or corrections, such as tests of normality and adjustment for multiple comparisons
☐	☒	A full description of the statistical parameters including central tendency (e.g. means) or other basic estimates (e.g. regression coefficient) AND variation (e.g. standard deviation) or associated estimates of uncertainty (e.g. confidence intervals)
☐	☒	For null hypothesis testing, the test statistic (e.g. *F*, *t*, *r*) with confidence intervals, effect sizes, degrees of freedom and *P* value noted<br>*Give P values as exact values whenever suitable.*
☒	☐	For Bayesian analysis, information on the choice of priors and Markov chain Monte Carlo settings
☒	☐	For hierarchical and complex designs, identification of the appropriate level for tests and full reporting of outcomes
☒	☐	Estimates of effect sizes (e.g. Cohen's *d*, Pearson's *r*), indicating how they were calculated

Our web collection on statistics for biologists contains articles on many of the points above.

Software and code

Policy information about availability of computer code

Data collection

No software was used to collect data in this study

Data analysis

For gene expression analyses: paired-end reads were pseudo-aligned and quantified to Gencode (GRCh38.p13) transcripts using the Kallisto program. Transcript level fragment counts were summarized to gene level using the TxImport Bioconductor package. Differential gene expression analysis was performed using the DESeq2 package. Gene expression was quantified in FPKM units for every gene using the DESeq2::fpkm function. Gene ontology analyses were done using the Goseq Bioconductor package.
For gene expression analysis in SAOS-2 cells lacking or overexpression JUN, paired-end reads were aligned to the reference genome using Hisat2 v2.0.5 [86]. FeatureCounts v1.5.0-p3 was used to count the reads numbers mapped to each gene. R package hclust was used for clustering genes while the cutree R package was used to generate subclusters. Differential expression analysis was performed using the DESeq2 R package (1.20.0).

For mass spectrometry analyses: For proteome and phosphoproteome, the raw files were processed with MaxQuant version 1.5.2.8 with preset standard settings for SILAC labeled samples . For the JUN IP, the raw files were processed with MaxQuant version 2.0.1.0.

For image analyses: FIJI (ImageJ 1.52p) software was used.

For combinatorial treatment analyses: SynergyFinderPlus was used.

For telomere length analysis (TRF): TeloTool v1.3 was used.

GraphPad Prism 9.4.1 (681) was used for data presentation and statistical analysis.

For manuscripts utilizing custom algorithms or software that are central to the research but not yet described in published literature, software must be made available to editors and reviewers. We strongly encourage code deposition in a community repository (e.g. GitHub). See the Nature Portfolio guidelines for submitting code & software for further information.

Data

Policy information about availability of data

All manuscripts must include a data availability statement. This statement should provide the following information, where applicable:

- Accession codes, unique identifiers, or web links for publicly available datasets
- A description of any restrictions on data availability
- For clinical datasets or third party data, please ensure that the statement adheres to our policy

-The RNA-sequencing data have been deposited to NCBI's Gene Expression Omnibus and are accessible through GEO Series accession number GSE190438 (https://www.ncbi.nlm.nih.gov/geo/query/acc.cgi?acc=GSE190438). For RNA-seq analysis, GRCh38.p13 dataset (Gencode) was used to align and quantify pair-ended reads.
-The mass spectrometry proteomics data have been deposited to the ProteomeXchange Consortium via the PRIDE partner repository with the dataset identifier PXD037501 (https://www.ebi.ac.uk/pride/archive/projects/PXD037501).

Human research participants

Policy information about studies involving human research participants and Sex and Gender in Research.

Reporting on sex and gender	not applicable
Population characteristics	not applicable
Recruitment	not applicable
Ethics oversight	not applicable

Note that full information on the approval of the study protocol must also be provided in the manuscript.

Field-specific reporting

Please select the one below that is the best fit for your research. If you are not sure, read the appropriate sections before making your selection.

☒ Life sciences ☐ Behavioural & social sciences ☐ Ecological, evolutionary & environmental sciences

For a reference copy of the document with all sections, see nature.com/documents/nr-reporting-summary-flat.pdf

Life sciences study design

All studies must disclose on these points even when the disclosure is negative.

Sample size	No sample-size calculation was performed. The sample size for each experiment was determined to ensure at least three biological replicates, which is sufficient to conduct statistical analyses.
Data exclusions	No data was excluded.
Replication	Replication and reproducibility of experiments were confirmed. When possible, experiments were performed by independent researchers to verify reproducibility.
Randomization	For in vivo studies, mice were assigned to each group in a way ensuring a comparable weight distribution in each group. For all the other experiments, the allocation was random.
Blinding	The investigators were blinded for the analysis of some immunostaining experiments (e.g: telomere aberrations (Figure 2F)). The images were numbered without identification before analyses.<br>For the rest of the experiments, the investigators were not blinded for group allocation during collection or analysis. Blinding was not necessary in these cases since it does no affect the results analyses and interpretation.

Reporting for specific materials, systems and methods

We require information from authors about some types of materials, experimental systems and methods used in many studies. Here, indicate whether each material, system or method listed is relevant to your study. If you are not sure if a list item applies to your research, read the appropriate section before selecting a response.

Materials & experimental systems

n/a	Involved in the study
☐	☒ Antibodies
☐	☒ Eukaryotic cell lines
☒	☐ Palaeontology and archaeology
☐	☒ Animals and other organisms
☒	☐ Clinical data
☒	☐ Dual use research of concern

Methods

n/a	Involved in the study
☒	☐ ChIP-seq
☒	☐ Flow cytometry
☒	☐ MRI-based neuroimaging

Antibodies

Antibodies used

Primary antibodies: clone/ source/ catalog #

γH2AX (pSer139 H2A.X): JBW301 Sigma 05-636
GAPDH: 0411 Cell signaling 2118
PML: PG-M3 Santa cruz sc-966
TRF2: polyclonal Novus biologicals NB110-57130
53BP1: polyclonal Novus biologicals NB100-304
pS33 RPA: polyclonal Bethyl laboratories A300-246
EPHA2: C-3 Santa cruz sc-398832
JUN: G-4 Santa cruz sc-74543
pS63 JUN: KM-1 Santa cruz sc-822
phospho-tyrosine (pY)-HRP: R&D systems Human phospho-RTK array, part 841403
ABL1: 24-11 Santa cruz sc-23
ABL1: Cell signaling 2862S
pT91/93 JUN: C-J 4C4/1 Santa cruz sc-53182
pS243 JUN: polyclonal ThermoFisher scientific PA5104747
β-actin: AC-74 Sigma A2228
RPA32/2: 9H8 Abcam ab2175
BrdU: IIB5 Santa cruz sc32323
ChromPure mouse IgG, whole molecule: Jackson ImmunoResearch 015-000-003
ATRX: polyclonal Santa Cruz sc-15408
SP100: 1G6 Santa Cruz sc-293458
PML: E-11 Santa Cruz sc-377390
FAM129A (NIBAN): F-10 Santa cruz sc-374636
ATF4 (CREB-2): B-3 Santa cruz sc-390063
P27 (CDKN1B/kip1): F-8 Santa cruz sc-1641
Phospho RB: E-10 Santa cruz sc-271930
Rb IF8: Santa cruz sc-102

Secondary antibodies: clone/ source/ catalog #

anti-mouse IgG Alexa Fluor 488: Cell signaling 4408
anti-Rabbit IgG Alexa Fluor 488: Cell signaling 4412
anti-mouse IgG Alexa Fluor 555: Cell signaling 4409
anti-mouse IgG Alexa Fluor 555: Life technologies A21424
anti-Rabbit IgG Alexa Fluor 488: Life technologies A11034
anti-mouse IgG Alexa Fluor 488: Life technologies A21202
Mouse TrueBlot® ULTRA: Anti-Mouse Ig HRP: eB144 Rockland antibodies and assays 18-8817-33
Rabbit TrueBlot®: Anti-Rabbit IgG HRP: eB182 Rockland antibodies and assays 18-8816-33
Anti mouse IgG-HRP: GE Healthcare NA931
Anti rabbit IgG-HRP: GE Healthcare NA934

Validation

All antibodies are validated commercially or by other research groups.

Primary antibodies validation
γH2AX (pSer139 H2A.X) manufacturer note: a well published antibody validated in ChIP, ICC, IF, WB. Cited in 2749 publications.
GAPDH manufacturer note: cited in 13568 publications, including 9173 publications for WB in human.
PML (PG-M3) manufacturer note: cited in 684 publications. Validated for IF application in human (PMID: 22213200)
TRF2 validated by genetic strategies by Novus Biologicals. Validated for IF application in human (PMID: 10669743; 15342490; 28082411)
53BP1 validated by genetic and biological strategies by Novus Biologicals. Cited in 594 publications, including 347 for IF application. IF application in human: (PMID: 19629043; 22365830)
pS33 RPA validated by the manufacturer for Western blot in humans after etoposide treatment; cited in 74 publications, including for

IF application in human (PMID: 23684611)
EPHA2 manufacturer note: cited in 24 publications; validated in this study by genetic knock-down of the protein
JUN manufacturer note: cited in 106 publications; validated in this study by genetic knock-down of the protein
pS63 JUN manufacturer note: cited in 580 publications, validated for WB for human by the manufacturer.
phospho-tyrosine (pY)-HRP manufacturer note: this kit has 218 citations (e.g for human: PMID: 34853306)
ABL1 (24-11) manufacturer note: cited in 192 publications; validated in this study by genetic knock-down of the protein
ABL1 manufacturer note: this antibody has 134 citations; validated for detection of human ABL1 by WB (PMID: 30559247)
pT91/93 JUN manufacturer note: cited in 12 publications; used for WB application in human (e.g: PMID: 32195020; 31685029)
pS243 JUN validated by the manufacturer by Western blot on phosphorylated and non phosphorylated peptides
β-actin manufacturer note: this antibody has 2532 citations, including for use for human WB
RPA32/2 manufacturer note: this antibody has 187 citations, including for use for human WB (e.g: PMID: 33500419; 30982887)
BrdU manufacturer note: cited in 236 publications, validated for immunoprecipitation (PMID: 30679424)
ATRX manufacturer note: cited in 30 publications, validated for human WB application (PMID: 21029860)
SP100 validated by manufacturer in overexpressing cells
PML (E-11) manufacturer note: this antibody has 19 citations, used and validated for human IF (PMID: 32183119; 32461215)
FAM129A (NIBAN) validated for WB use in human by the manufacturer
ATF4 (CREB-2) B-3 manufacturer note: this antibody has 86 citations, validated for WB for human protein (PMID: 32121537)
P27 (CDKN1B/kip1) F-8 manufacturer note: this antibody has 647 citations, validated for human WB use (PMID: 12531224; 32258031)
Phospho RB E-10 manufacturer note: this antibody has 14 citations, used for WB in human (PMID: 32559461)
Rb IF8 manufacturer note: this antibody has 363 citations, used for WB in human (e.g: PMID:9242374)

Eukaryotic cell lines

Policy information about cell lines and Sex and Gender in Research

Cell line source(s)	U2OS, HOS, HT161, MG63, 293T Phillip Koeffler lab; purchased from ATCC<br>SAOS-2, IMR90 Dennis Kappei lab; original source ATCC<br>CAL72 kindly provided by Dr. Natalie Rochet<br>SW26, SW39 kindly provided by Prof Jerry W. Shay<br>T1000, T778 kindly provided by Dr. Florence Pedeutour<br>TG16, TG20 kindly provided by Dr. François Boussin
Authentication	CAL72; HOS; MG63; SAOS-2; T778 and U2OS were authenticated by STR profiling. The other cell lines were not authenticated.
Mycoplasma contamination	Cell lines were tested negative for mycoplasma .
Commonly misidentified lines (See ICLAC register)	None used.

Animals and other research organisms

Policy information about studies involving animals; ARRIVE guidelines recommended for reporting animal research, and Sex and Gender in Research

Laboratory animals	6-8 weeks old NSG (NOD-SCID gamma) female mice were used in this study
Wild animals	The study did not involve wild animals
Reporting on sex	While our study involved only subcutaneous xenograft experiments, we used same sex mice (in this case, females) for the control and treatment groups, to avoid any potential sex-biased effect.
Field-collected samples	The study did not involve samples collected from the field
Ethics oversight	In vivo experiments were performed in compliance with ethical regulations of Institutional Animal Care and Use Committee (IACUC) of National University of Singapore.

Note that full information on the approval of the study protocol must also be provided in the manuscript.
