# Supplementary material for: Signalling inhibition by ponatinib disrupts productive alternative lengthening of telomeres (ALT)
Source: Nat Commun. 2023 Apr 6;14:1919. doi: 10.1038/s41467-023-37633-3 (PMC10079688; doi:10.1038/s41467-023-37633-3)
Supplement: Supplementary file 2 — Peer Review File [file 41467_2023_37633_MOESM2_ESM.pdf]

nature portfolio

Peer Review File

Signalling Inhibition by Ponatinib Disrupts Productive Alternative Lengthening of Telomeres (ALT)

Open Access This file is licensed under a Creative Commons Attribution 4.0 International License, which permits use, sharing, adaptation, distribution and reproduction in any medium or format, as long as you give appropriate credit to the original author(s) and the source, provide a link to the Creative Commons license, and indicate if changes were made. In the cases where the authors are anonymous, such as is the case for the reports of anonymous peer reviewers, author attribution should be to 'Anonymous Referee' followed by a clear attribution to the source work. The images or other third party material in this file are included in the article's Creative Commons license, unless indicated otherwise in a credit line to the material. If material is not included in the article's Creative Commons license and your intended use is not permitted by statutory regulation or exceeds the permitted use, you will need to obtain permission directly from the copyright holder. To view a copy of this license, visit http://creativecommons.org/licenses/by/4.0/.

Editorial Note: Parts of this Peer Review File have been redacted as indicated to remove third-party material where no permission to publish could be obtained. Parts of this Peer Review File have also been redacted to maintain the confidentiality of unpublished data.

REVIEWER COMMENTS

Reviewer #1 (Remarks to the Author):

In this manuscript, Kusuma et al show that Ponatinib, a receptor tyrosine kinase inhibitor, can kill ALT cancer cells which are relying on a telomerase-independent telomere maintenance mechanism. Ponatinib treatment leads to increase in ALT pathway, such as increase C-circle level, DNA replication stresses at telomeres, and single stranded telomeres etc. While this manuscript is written well and the flow is good, however, there are some major issues to be addressed, such as technical concerns in experimental procedure. Moreover, it is difficult to interpret the southern blots; (both Telomere gel blot and dot blot) some of which are saturated and not suitable quality for the publication in high profile journal.

1. Telomere probe issue: While the author performed all telomere southern using the TeloTAGGG telomere length Assay kit. the sequence of TelomereTAGGG telomere probe is unclear. Particularly, for the C-circle assay, C-rich sequence probe should be used. The probe validation should be included.
1a. Fig1C, S1D-F. C-circle assay: The films are fully saturated, not controlled. To avoid saturation issue, the author should perform at least three different amounts of genomic DNA for the quantification.
1b. Fig3A-B. Given C-circle assay result, this reviewer assume that TeloTAGGG probe is a C-rich probe. Since this manuscript is about ALT cells, The ssDNA measurement using G-rich probe should be included. Input blot (denaturing blot) should be done in same experimental condition as native condition, or the blot needed to be denatured after taking native condition. ExoIII treated DNA also should be run in the gel as a control of ExoIII reaction.
1c. Fig3B-D. Input are all saturated.
1d. FigS2D-E. Fragmented DNAs in EtBr staining condition might indicate that DSBs are also generated in genome-wide.
1e. FigS2G.The quality of telomere blot is not good enough to support the idea that single stranded telomeres were generated in Ponatinib treatment condition. Also, should include a loading control, such as EtBr staining.

2. ATSA (ALT telomere DNA synthesis in APBs) assay, (Zhang et al., 2019, Cell reports) should be done in Saos2 and U2OS cells to measure the ALT telomere synthesis.

Reviewer #2 (Remarks to the Author):

Title: Signalling Inhibition by Ponatinib Disrupts Productive Alternative Lengthening of Telomeres (ALT)
Authors: FK Kusuma, A Prabhu, and co-authors

Comments:
This work by Kusuma, Prabhu and co-authors represents an effort to describe the role of kinase inhibitor, Ponatinib, to telomere homeostasis in ALT cancer cells. To assess the mechanisms, the authors used panel of methods, including mass spectrometry and RNA-seq. Their findings are that Ponatinib inhibits ABL1-JNK-JUN signaling pathway, and that JUN might play a role in DNA damage repair pathways.

1. Please rewrite this part in Material and Methods: 'Titanium dioxide (TiO_2) beads (GL Sciences) were washed with solution C, added to the peptides (ratio 1:1 w/w) and incubated for 1 hour on a rotator at room temperature. The supernatant was then removed, and the beads loaded on C8 tips, washed with solutions C and B before eluting the enriched peptides with NH_4OH (400mM). Formic acid was added to the samples before loading them on C18 stage tips for mass spectrometry'. It gives the impression

that from the first sentence beads are in bulk and added to the samples, and then later it says the beads are in C8 tip-columns? Please give details of how much peptide was used to mix with the beads for phosphoproteomic analysis. It says w/w, but I am not sure how you weigh out your peptide pellet for this. I need more information in the text. I also assume that NH4OH was evaporated before formic acid was added to the samples? Were samples neutralized at all?
2. Please give details of the amount of peptides that was loaded and analyzed on LC-MS/MS.
3. Label-free identification of JUN interactome part, needs to be rewritten. Label-free quantitation (LFQ) is one of the methods in proteomics commonly used to quantify (relative values) proteins. The method name in this section is not appropriate for what the authors actually did. If I understand this part correctly, the aim here was to identify JUN interacting proteins, using immunoprecipitation. How was JUN immunoprecipitated for this experiment? I couldn't find details for this experiment. In the section, the authors say 'Immunoprecipitation experiments were performed on non-labelled SAOS-2 nuclear extracts as described above', however I couldn't find details for this above. There is a section for immunoprecipitation, that describes using less than 2 ug of antibodies per reaction, which is not enough for large scale experiments. We commonly use around 30-50 ug of antibodies to pool-down a protein for mass spectrometry analysis. Can you explain? My next question in this section is, how are the authors sure that reported interactors are real. What strategy was used to eliminate common contaminants and sticky proteins?
4. I have a technical question for Table S4: what do values in column C (number of proteins) represent here exactly?

Reviewer #3 (Remarks to the Author):

This work from Kusuma et al. explores the application of Ponatinib as an agent to selectively kill ALT cancer cells. Ponatinib is an available small molecule inhibitor of chimeric BCR-ABL kinase. Here the authors show that Ponatinib treatment deregulates ALT activity, causing an increase in C-circles, and promoting telomere damage and dysfunction. Examining a panel of ALT cancer cell lines, the authors show that Ponatinib treatment causes the accumulation of DDR associated activity (γH2AX and 53BP1), TERRA deregulation, replicative stress (RPA foci) and the acquisition of cellular senescent phenotypes such as beta-galactosidase positivity. Taking their findings in vivo, the authors show that Ponatinib suppresses tumor growth in xenograft mouse models. In pursuit of a target of Ponatinib, the authors conduct extensive phospho-proteomics and RNA sequencing before identifying JUN kinase as a candidate target. Subsequent validation experiments of JUN deficient cells reveal patterns consistent with JUN's involvement in regulating ALT and survival of ALT cancer cells. Lastly, the authors provide evidence of potential synergism of Ponatinib with other DNA repair factor inhibitors (CHK and ATM kinases).

The study is extensive, with a huge and impressive range of experimentation. They have apparently thrown a lot of effort and consideration into this study. Yet there are some important shortcomings (all Minor and major) that should be addressed before the manuscript could be accepted for publication. Addressing these would benefit the paper and the telomere/ALT field.

1. Throughout the study, the variation in outcomes of experiments is pretty striking. Within a single experiment, some experiments appear to show no or minimal effect. Yet, there are frequent outliers whose inclusion certainly augments the apparent effects of Ponatinib. (See Figure 1D, 2G, 2L, 3B, 4B, 6A, 6C). Some of the supporting blots for CC assays are not very convincing. The variation raises concerns of the consistency of outcomes in this study, and possibly future independent studies. This is an important issue.
2. The claims that Ponatinib causes replicative stress that drives cell fate outcomes is important and could be strengthened.
a. the authors claim that TERRA are increased. In fact, this increase is rather minimal. This should be re-examined.

b. The authors claim that APBs are not altered. This is of interest. But it seems that only Saos2 cells were examined (S2A). But in 2J, there seems to be an effect on telomere clustering. Is this APB/PML independent? Suggest examining APBs in other cell lines and verification of clustering effects.
c. The experiments to assess DNA nicks and gaps are interesting. Recent work from the Cantor lab has applied S1 nuclease instead of ExoIII to detect gaps in newly replicated DNA and this approach seems to be superior to what is presented here. It would be optimal to reassess the gap hypothesis to support the authors conclusions.
d. What is the mechanism of senescence activation here? It appears that p53 is not required. Is this driven by micronuclei and the cGAS pathway?
e. The effects of Ponatinib in normal, healthy cells are not examined.
3. Are the ALT cell lines used that exhibit sensitivity to Ponatinib ATRX-DAXX mutated? This could be added if there is a bias.
4. The in vivo studies are important and really going in a strong direction. Yet, the telomere length analysis is not at all convincing. This should be re-examined using Pulse Field Gel Electrophoresis (PFGE). Alternatively, have the authors considered doing TRF analysis with a lower, chronic dose of Ponatinib to explore telomere length alterations.
5. In Figure 6A and D, it would be a nice experiment to show that the increases of C-circles/telomere integrity can be suppressed in the JUN KO cells and restored in the complemented cells. Do JUN KO cells show defects in telomere integrity and replication? This would further corroborate the evidence that JUN has an important role in telomere maintenance.
6. In Figure 6C, what is the reference for calculation? Is it LacZ in OE-GFP for both sets of experiments? Considering fold change, it looks to me that the effect of OE-JUN is in fact minimal. Correct?
7. The blots in 6G are uninterpretable. This experiment should be repeated and an improved blot presented.
8. In Figure 7D, Ponatinib does not induce gH2AX in Saos2 cells. This is a lower dose 125nM instead of 250nM which is used throughout the rest of the story. Why was this the case?
9. Lastly, the data in the TG20/T16 glioma cell lines are very limited and could be expanded to further show the effects of Ponatinib on ALT.

Reviewer #4 (Remarks to the Author):

This is a potentially very interesting study that demonstrates effects of a clinically-approved drug on the telomere biology and viability of ALT cancer cells. The effects of this compound on telomere integrity in ALT cells appear to be well-supported by the data. Its effects on tumor growth in vivo are also particularly striking. However, I think there is quite a bit of work remaining to shore up the main conclusions of the study and the mechanism for the observed effects, as outlined below. With these extra data, this study has the potential to be a very important contribution to the field.

Major comments:

1) The introduction (line 63) cites a study showing a tendency for ALT cell lines to be particularly sensitive to ATR inhibitors; however, this conclusion was later brought into question (Deeg et al, Front Oncol 2016; Laroche-Clary et al Scientific Reports 2020). For this reason, it is necessary to apply a great deal of caution when claiming ALT-specific sensitivity to any drug. I would therefore like to see the following additional lines of evidence for ALT-specific sensitivity to ponatinib: i) comparison of a larger panel of ALT vs Tel cell lines than just 4 of each, ii) confirmation of relative drug sensitivities using a second assay type, and iii) comparison of an isogenic pair of cell lines in which ALT has been inhibited in one of them by overexpression of ATRX, as performed in Deeg et al 2016.

2) Since the authors showed that an ALT cell line with WT p53 (U2OS) did not experience global DNA damage in response to ponatinib (Fig S2C), the panel of cell lines referred to in my previous comment should include both p53 WT and p53-deficient TEL cell lines, to determine whether ponatinib sensitivity correlates with p53 status.

3) The ALT-specificity of the observations would also be strengthened by: i) determining whether knockdown of JUN impacts viability of TEL cell lines, and ii) determining whether the synergistic effect of ponatinib and triciribine extended to other ALT cell lines, and was not observed in TEL cell lines.

4) It is stated in the Discussion that "ponatinib's impact on telomeres and DNA damage is specific to ALT cells", but, except for effects on global gH2AX levels and C-circles (which TEL cells don't have), there wasn't any evidence presented for this. Does ponatinib affect levels of TIFs, telomere aberrations or telomere nicks in TEL cells, or in ALT cells other than SAOS-2 (the only cell line used in Fig 2E-J)?

5) A great deal of experimental effort was invested in determining that Abl1 mediates phosphorylation of JUN on several sites (Y170, S63, T91/93) via JNK kinases (for the latter two sites), but it's not clear to me that any of these phosphorylation events have anything to do with effects on ALT cell telomeres or viability, since a JNK inhibitor did not affect levels of C-circles (Fig S6L). This should be possible to test, using a rescue experiment like that in Figure 6C, but with phosphodeficient or phosphomimetic versions of JUN at each of these sites.

6) An effect of JUN on C-circle levels is demonstrated, but it's not clear if JUN affects any of the other markers of telomere integrity shown in Figs 2 and 3 (TIFs, telomere aberrations, telomeric nicks/gaps). The overall involvement of JUN in cellular viability via telomeres would be strengthened by measurement of at least one or two of these parameters after JUN knockdown.

7) By the end of the paper, the authors conclude that it is JUN's interaction with several DNA repair factors that is likely to be the cause of its effects on ALT telomeres, but there is no direct evidence for this. Does depletion of any of the identified interactors (lines 296, 297) recapitulate the effects of ponatinib or JUN knockdown on markers of telomere integrity in ALT cells?

8) The effects of ponatinib on CAL72 cells was different in vitro and in vivo, and this difference was not addressed in the text. Ponatinib caused an increase in t-circles in vitro but a decrease in vivo; it had no apparent effect on telomere length in vitro, but resulted in shorter telomeres in vivo. What are some possible reasons for these differences?

Minor comments:

i. Figure 1: for the viability assays, it is essential to specify in the figure legend the length of time for which cells were treated, in order to distinguish viability effects resulting from gradual telomere shortening (which would be expected to take time to manifest) from more rapid effects.

ii. Figure 1B: it's not clear if the data in the right graph represent one ALT cell line and one TEL cell line (if so, which ones?), or a compilation of data from all the cell lines in the left graph?

iii. Should the cell line referred to as H161 instead be HT161? (https://web.expasy.org/cellosaurus/CVCL_C834).

iv. The labelling in Figure 6E is not large enough to read.

v. The right panel of Figure 6G (Y170F JUN) is not very convincing because the background is so dark; this experiment should be repeated.

Response to reviewers' comments

Reviewer #1 (Remarks to the Author):

In this manuscript, Kusuma et al show that Ponatinib, a receptor tyrosine kinase inhibitor, can kill ALT cancer cells which are relying on a telomerase-independent telomere maintenance mechanism. Ponatinib treatment leads to increase in ALT pathway, such as increase C-circle level, DNA replication stresses at telomeres, and single stranded telomeres etc. While this manuscript is written well and the flow is good, however, there are some major issues to be addressed, such as technical concerns in experimental procedure. Moreover, it is difficult to interpret the southern blots; (both Telomere gel blot and dot blot) some of which are saturated and not suitable quality for the publication in high profile journal.

We thank the reviewer for their comments and have addressed their concerns below.

1. Telomere probe issue: While the author performed all telomere southern using the TeloTAGGG telomere length Assay kit. the sequence of TelomereTAGGG telomere probe is unclear. Particularly, for the C-circle assay, C-rich sequence probe should be used. The probe validation should be included.

Indeed, the exact sequence of the telomeric probe in the TeloTAGGG kit is not disclosed by the manufacturer. The kit's manual does mention, however, that it allows: *'Sensitive detection of telomeric DNA (telomeric sequence: TTAGGG)'.* We have confirmed this with Roche's technical team which replied that: '*The DIG-labeled probe (vial 9) is an oligonucleotide containing a complementary sequence specific to hybridize to telomeric repeats (sequence: TTAGGG in humans). The sequence of the DIG-labeled probe in vial 9 and the method of generating it are confidential.'*

In addition, in our C-circle assays, dCTP is omitted from the phi29-based amplification to specifically amplify the C-circles. Hence, the amplified G-rich products can only be detected with a C-rich probe. Our C-circle protocol has been also validated: signals are detected only in ALT-positive cells, in the +phi29 reaction and not in reactions without the polymerase, and these C-circles are lost when PML, the main component of the APBs, is down-regulated (**new data - Supplementary Figure 2f**).

Moreover, we confirmed that ponatinib increases the levels of telomeric C-circles by using a DIG-labeled C-rich probe ($(CCCTAA)_4$) (**new data - Supplementary Figure 2d**). The protocol of the probe labelling is now included in the Supplementary Methods section.

1a. Fig1C, S1D-F. C-circle assay: The films are fully saturated, not controlled. To avoid saturation issue, the author should perform at least three different amounts of genomic DNA for the quantification.

We agree with the reviewer that the C-circle assays in the mentioned figures contain some saturated signals in some treatment conditions.

Based on the standard protocol established by Roger Reddel's lab (Henson JD et al [1] and Henson JD et al, [2]), the linearity of the C-circle assay is compromised if high amounts of genomic DNA is used (Henson et al suggest using less than 40 ng of genomic DNA for the rolling circle amplification). In agreement with that, our optimization testing also indicated that the optimal quantity range of DNA to use is 5-15 ng for the tested cell lines.

In our experiments, we regularly use 5-7.5 ng for the rolling circle amplification, perform different exposure times of the membrane and quantify the exposure that gives the minimum quantifiable signal in the control sample (e.g., DMSO samples). In some assays, C-circle induction by ponatinib

treatment was too strong to obtain an exposure where the control signals are quantifiable (e.g., in **new data- Supplementary Figure 2d**: despite loading less amount of the CC products, a quantification would only be possible if the control samples are detected). Hence, we performed our quantifications using the exposure that allows a minimum detection of the control samples.

We have replaced the blots in Supplementary Figure S1D **(now Supplementary Figure 2a)** with another representative C-circle assay on these cell lines.

We have replaced Supplementary Figure S1F **(now Supplementary Figure 2c)** with a lower exposure time of the dot blot.

1b. Fig3A-B. Given C-circle assay result, this reviewer assume that TeloTAGGG probe is a C-rich probe. Since this manuscript is about ALT cells, The ssDNA measurement using G-rich probe should be included. Input blot (denaturing blot) should be done in same experimental condition as native condition, or the blot needed to be denatured after taking native condition. ExoIII treated DNA also should be run in the gel as a control of ExoIII reaction.

Re: Since this manuscript is about ALT cells, The ssDNA measurement using G-rich probe should be included.

The exonuclease assay for detection of ssDNA breaks and gaps is adapted from Zhang et al [3]. In their study, the authors performed an in-gel hybridization of the reactions using either a C-rich or a G-rich probe. They show that the breaks are present predominantly on the C-rich strand of telomeric DNA in U2OS cells (see below from their study).

[redacted]

From [3]-Fig3: Endogenous ssDNA break/gap or induced ssDNA break in C-rich strand stimulates formation of C-circles and 5' C-overhangs. (A) Experimental protocol to study strand specific (G-rich or C-rich) breaks/gaps on telomere is shown schematically. HinfI and RsaI digested genomic DNA was purified and further digested with Exo III to examine potential breaks/gaps on G-strand or C-strand of telomeres. If breaks/gaps occur on C-strand, Exo III would degrade all C-strand, leaving single-stranded G-strand that can be detected by hybridization with C-rich probe under native or denatured condition. Contrariwise, only C-strand can be detected if breaks/gaps occur on G-strand. (B) Breaks/gaps occur more frequently on C-rich strand of telomere.

To confirm that these breaks are more frequent on the C-strand, we repeated the exonuclease reactions on U2OS cells using our dot blot protocol. The same +/- exo reactions were divided into two samples and loaded in parallel on a membrane. The membrane was then cut and hybridized with either a C-rich or a G-rich probe to compare the signals generated after exonuclease digestion. Like the findings of Zhang et al, telomeric breaks/gaps were more significantly detected on the C-strand (**new data - Supplementary Figure 5a**). The sequence and labelling protocol of the G-rich probe are included in the Supplementary Methods section.

Re: Input blot (denaturing blot) should be done in same experimental condition as native condition, or the blot needed to be denatured after taking native condition.

The input blot is indeed performed in the same experimental condition as the native blot. After genomic DNA digestion with HinfI and RsaI and DNA purification, the eluted DNA is divided into three tubes (one for the no exo reaction, one for the + exo reaction and one is kept at -80°C to be used as input). The next day, following exonuclease digestion and sample purification, these samples are blotted on a membrane, together with the denatured thawed input samples. The membrane is then cut and the +/- exoIII samples are subjected to a native dot blot while the input samples are subjected to a denaturing dot blot in parallel.

Re: ExoIII treated DNA also should be run in the gel as a control of ExoIII reaction.

The appearance of a signal in native dot blot in the + ExoIII but not in the - ExoIII reactions indicates that the ExoIII was active (Figure 3a-b and Supplementary Figure 5a).

1c. Fig3B-D. Input are all saturated.

The reviewer may have missed it but, in Figures 3b and 3c, we show short exposures (unsaturated blots), as well as a longer exposure time. The quantification of the input was done on the short exposure blots. Concerning Figure 3d, we have now included in the same figure a shorter exposure of this blot.

1d. FigS2D-E. Fragmented DNAs in EtBr staining condition might indicate that DSBs are also generated in genomewide.

The DNA fragments obtained after ponatinib treatment (in now Supplementary Fig. 4a-b, previously Fig. S2D-E) are reminiscent of the fragments seen after topoisomerase inhibition by SN-38 [4]. We have previously sequenced these fragments and found that they were significantly enriched for subtelomeric regions [4]. However, we do agree with the reviewer that ponatinib might cause genomic DNA damage in addition to telomeric damage. We do mention this induction of global DNA damage in the text (lines 133 and 139-142).

1e. FigS2G.The quality of telomere blot is not good enough to support the idea that single stranded telomeres were generated in Ponatinib treatment condition. Also, should include a loading control, such as EtBr staining.

The aim of the TRF assays in FigS2G (now Supplementary Figure 4d) is to verify whether ponatinib treatment induces telomere elongation (as mentioned in line 154). We noticed smaller telomere fragments (not necessarily only single stranded) in SAOS-2 cells. We have, however, removed this hypothesis from the text as well as the first TRF blot of SAOS-2, to avoid confusion. In parallel, we have now included the EtBr staining for these TRF gels.

2. ATSA (ALT telomere DNA synthesis in APBs) assay, (Zhang et al., 2019, Cell reports) should be done in Saos2 and U2OS cells to measure the ALT telomere synthesis.

As suggested by the reviewer, we performed ATSA assay to detect telomere synthesis in APBs in G2 cells based on the protocol described in Zhang et al [5]. U2OS or SAOS-2 cells were treated first with either DMSO or ponatinib, then after 3 hours, a CDK1 inhibitor (RO-3306) was added for 15-16 hours to synchronize the cells in G2. Afterwards, EdU was added for 2 hours, and cells were fixed and stained for telomeric DNA (using a TelG-PNA-probe), EdU (using a Click-iT reaction), and PML bodies (using an antibody against SP100, a component and marker of the PML bodies [6] (we used this antibody because of its better and consistent performance in our hands compared to the latest batches of PML antibodies). A detailed protocol of the ATSA assay is now included in the Methods section.

By scoring the number of APBs per APB-positive cell, as well as the number of EdU + APBs, we found that ponatinib treatment reduced telomeric synthesis in APBs in both U2OS and SAOS-2 cells **(new data - Figure 3e-f)**, supporting the results obtained with the BrdU pull-down assays (Figure 3 c-d).

Reviewer #2 (Remarks to the Author):

Title: Signalling Inhibition by Ponatinib Disrupts Productive Alternative Lengthening of Telomeres (ALT)

Authors: FK Kusuma, A Prabhu, and co-authors

Comments: This work by Kusuma, Prabhu and co-authors represents an effort to describe the role of kinase inhibitor, Ponatinib, to telomere homeostasis in ALT cancer cells. To assess the mechanisms, the authors used panel of methods, including mass spectrometry and RNA-seq. Their findings are that Ponatinib inhibits ABL1-JNK-JUN signaling pathway, and that JUN might play a role in DNA damage repair pathways.

We thank the reviewer for their comments, and we have addressed their questions below.

1. Please rewrite this part in Material and Methods: 'Titanium dioxide (TiO_2) beads (GL Sciences) were washed with solution C, added to the peptides (ratio 1:1 w/w) and incubated for 1 hour on a rotator at room temperature. The supernatant was then removed, and the beads loaded on C8 tips, washed with solutions C and B before eluting the enriched peptides with NH_4OH (400mM). Formic acid was added to the samples before loading them on C18 stage tips for mass spectrometry'. It gives the impression that from the first sentence beads are in bulk and added to the samples, and then later it says the beads are in C8 tip-columns? Please give details of how much peptide was used to mix with the beads for phosphoproteomic analysis. It says w/w, but I am not sure how you weigh out your peptide pellet for this. I need more information in the text. I also assume that NH_4OH was evaporated before formic acid was added to the samples? Were samples neutralized at all?

We apologise for not being clearer in the methods description during the original submission.

Indeed, the beads are in solution during the incubation but as part of the clean-up step, they were applied to C8 stage tips for washing and elution, i.e., the packing material within the tip serves as a cushion to retain the beads while the washing and elution solutions can flow through.

Formic acid was added to the samples directly after NH_4OH elution to neutralize the samples, followed by standard de-salting step with C18 stage tips.

As suggested, we have now amended the text and provided overall more details in the Supplementary Methods section.

2. Please give details of the amount of peptides that was loaded and analyzed on LC-MS/MS.

We do not routinely quantify peptide amounts prior to loading for LC-MS/MS analysis. Rather, we normalise the protein quantities that are used as starting material for different applications (here: 5 mg for phosphoproteomics, 200 µg for proteomes, 300 µg of nuclear extracts for IPs) and we then load equal volumes of the desalted samples once eluted from C18 stage tips. We have now described these steps in detail as mentioned above. Concretely, for each sample, we obtain a final volume of 12 µl out of which 6 µl are placed on the HPLC, and we pick-up 5ul for injection. The remaining 6 µl are frozen and serve as a back-up in case any problem was encountered during the LC-MS/MS runs. Hence, effectively each reaction is based on half of the total input material.

3. Label-free identification of JUN interactome part, needs to be rewritten. Label-free quantitation (LFQ) is one of the methods in proteomics commonly used to quantify (relative values) proteins. The method name in this section is not appropriate for what the authors actually did. If I understand this part correctly, the aim here was to identify JUN interacting proteins, using immunoprecipitation. How was JUN immunoprecipitated for this experiment? I couldn't find details for this experiment. In the section, the authors say 'Immunoprecipitation experiments were performed on non-labelled SAOS-2 nuclear extracts as described above', however I couldn't find details for this above. There is a section for immunoprecipitation, that describes using less than 2 ug of antibodies per reaction, which is not enough for large scale experiments. We commonly use around 30-50 ug of antibodies to pool-down a protein for mass spectrometry analysis. Can you explain? My next question in this section is, how are the authors sure that reported interactors are real. What strategy was used to eliminate common contaminants and sticky proteins?

Again, we apologise for being too brief in the Methods section, and we now provide a more detailed description of the LFQ JUN interaction analysis in the Supplementary Methods section.

Indeed, we used quadruplicate reactions for each condition to immunoprecipitate JUN with antibody (sc-74543) in both SAOS-2 WT or sgJUN KO cells or SAOS-2 DMSO vs. SAOS-2 Ponatinib treated cells. Quadruplicate reactions were analysed by LC-MS/MS and with the MaxLFQ algorithm [7] to convert MS1 intensities into quantitative LFQ intensities for each quantified protein. The MaxQuant output flags common contaminants, reverse identifications and by site only identifications, which were all removed from the data. Subsequently, we log2 transformed all LFQ intensities and imputed missing values if in at least one of the experimental conditions 4 experimental values were obtained (e.g., all 4 replicates in the SAOS-2 WT samples for a specific protein were experimentally determined based on MS1 and MS2 spectra but samples in the SAOS-2 sgJUN control had missing values). Here, we generated a normal distribution from the lowest 5% of the measured intensity values and randomly drew values for imputation. We ran three separate iterations for this process and generated average values. The resulting variation is reported both as standard deviations for the fold change and p-value calculations and this is visualised in the volcano plots as two-dimensional error bars (displayed for the proteins above the cut-offs only for a better readability) (Figure 7a-b).

Two aspects in this analysis ensure that we report real interactors differentiated from sticky background proteins: (1) Using the affinity enrichment strategy described above allows us to quantitatively differentiate between genuinely enriched proteins and background proteins that correspond to data points at the bottom of the volcano plot. (2) By using a sgJUN KO control we rigorously control for antibody-specific background that the widely used standard IgG control cannot fully exclude.

Finally, we did indeed use 2 µg of antibody for each replicate reaction. Beyond our specific optimisation to find an ideal ratio between beads and antibodies vs. protein input material, the described quantities are not unusual, and we have used similar amounts in other publications (e.g., [8]). First, the utilized quantity is in line with the binding capacity of the 15 µl of magnetic Dynabeads Protein G that were used in this assay and is in line with the recommended range of 1-10 µg of antibody for each 50 µl of beads, based on the manufacturer's instructions. Furthermore, even several large-scale efforts with immunoprecipitations of thousands of tagged proteins used similar equivalents of antibody-coupled beads (e.g., [9, 10]). Moreover, all these quantities represent just a compromise for a standard protocol that works in average as different needs for reagent quantities do not only depend on the affinity/quality of the specific antibody, and the sensitivity of the LC-MS/MS setup but more strongly on the expression level of the bait protein. In other words, for a very lowly expressed bait protein adding more antibody (assuming the protein input quantity is kept constant)

will not improve the signal any further and in fact this protein might already be depleted from the protein extract. Hence, more antibody quantity can also mean overuse of reagents, and potential weaker signal-to-noise ratio due to the use of more beads, which inherently will increase the background signal from sticky proteins.

In sum, we have used state-of-the-art procedures to quantitatively identify JUN interaction partners by label-free quantitative mass spectrometry analysis, and we have clarified the experimental details in the Methods section accordingly.

4. I have a technical question for Table S4: what do values in column C (number of proteins) represent here exactly?

Column C in Table S4 (now Supplementary Table 4) corresponds to the number of proteins contained within each row. Due to the incomplete sequence coverage of LC-MS/MS data, researchers typically report “protein groups”. For instance, for JUN as the bait protein, we report 2 proteins within this group, namely P05412 & P17535, which correspond to two isoforms of JUN with 331 and 347 amino acids, respectively. Since we did not obtain unique peptides that can differentiate both isoforms, the MaxQuant analysis bins both isoforms sharing alignment for the identified 11 peptides. This logic of protein groups can refer both to isoforms of the same gene body (i.e., the same gene name), but also to proteins from separate genes that share high sequence identity and that could not be differentiated with unique peptides based on the data. For instance, H2AFV and H2AFZ are two separate genes located on chromosomes 7 and 4, respectively, but are listed together in the same row of our proteomics data. Both proteins differ in just 3 amino acids, and in the absence of a unique peptide that would clearly differentiate between both isoforms, we cannot know whether we identified one of them specifically or both.

All MS data have been uploaded to the PRIDE repository including the raw MS files, as well as the entire data output from MaxQuant, which contains further detailed information beyond what we are reporting in the Supplementary Tables.

Reviewer #3 (Remarks to the Author):

This work from Kusuma et al. explores the application of Ponatinib as an agent to selectively kill ALT cancer cells. Ponatinib is an available small molecule inhibitor of chimeric BCR-ABL kinase. Here the authors show that Ponatinib treatment deregulates ALT activity, causing an increase in C-circles, and promoting telomere damage and dysfunction. Examining a panel of ALT cancer cell lines, the authors show that Ponatinib treatment causes the accumulation of DDR associated activity (H2AX and 53BP1), TERRA deregulation, replicative stress (RPA foci) and the acquisition of cellular senescent phenotypes such as beta-galactosidase positivity. Taking their findings in vivo, the authors show that Ponatinib suppresses tumor growth in xenograft mouse models. In pursuit of a target of Ponatinib, the authors conduct extensive phospho-proteomics and RNA sequencing before identifying JUN kinase as a candidate target. Subsequent validation experiments of JUN deficient cells reveal patterns consistent with JUN’s involvement in regulating ALT and survival of ALT cancer cells. Lastly, the authors provide evidence of potential synergism of Ponatinib with other DNA repair factor inhibitors (CHK and ATM kinases).

The study is extensive, with a huge and impressive range of experimentation. They have apparently thrown a lot of effort and consideration into this study. Yet there are some important shortcomings (all Minor and major) that should be addressed before the manuscript could be accepted for publication. Addressing these would benefit the paper and the telomere/ALT field.

We thank the reviewer for their appreciation of the extensive investigation in this study. We have addressed their concerns below.

1. Throughout the study, the variation in outcomes of experiments is pretty striking. Within a single experiment, some experiments appear to show no or minimal effect. Yet, there are frequent outliers whose inclusion certainly augments the apparent effects of Ponatinib. (See Figure 1D, 2G, 2L, 3B, 4B, 6A, 6C). Some of the supporting blots for CC assays are not very convincing. The variation raises concerns of the consistency of outcomes in this study, and possibly future independent studies. This is an important issue.

We understand the reviewer's concerns. Besides Figure 4b where heterogeneity is within one experiment and due to the difficult engraftment properties of ALT CAL72 cells in mice, the graphs in the other mentioned figures represent a compilation of biological replicates including experiments performed either independently, or by different individuals and even in some cases in different lab settings. Moreover, two major findings of the paper (Figures 1d, 6a, 6c) have been validated at least once more either during the revision or in other experiments in the study. For instance, the effect of ponatinib on telomeric C-circles is reproduced in Figures 1d, 7e and Supplementary Figures 2d, 2f (**new data**), 8g (**new data**), 9h and 13c. The increase of telomeric C-circles after JUN down-regulation is observed in Figures 6a-c and Supplementary Figure 8g (**new data**).

Re: Some of the supporting blots for CC assays are not very convincing.

We have replaced some of the blots for the C-circle assays in Supplementary Figure 2a (previously Supplementary Figure S1D) with different representative blots. We have also replaced Supplementary Figure 2c (previously Supplementary Figure S1F) with a dot blot exposure for a shorter time.

2. The claims that Ponatinib causes replicative stress that drives cell fate outcomes is important and could be strengthened.

a. the authors claim that TERRA are increased. In fact, this increase is rather minimal. This should be re-examined.

The average increase of TERRA after either ponatinib or PD173074 treatment ranged between 2- to 3-fold, on at least 4 out of 5 tested chromosome-specific TERRA. While relatively moderate, this increase was consistent and statistically significant over several independent experiments. These changes are similar to the 2- to 4-fold increase of TERRA at damaged telomeres after TRF2 depletion (Porro et al, Nat com, 2014 [11]) and to the 2.5-3.5 -fold increase after inducing replication stress at telomeres by depleting FANCM (Silva et al, Nat com, 2019; [12]). We have cited these references in the original manuscript (lines 144-146).

b. The authors claim that APBs are not altered. This is of interest. But it seems that only Saos2 cells were examined (S2A). But in 2J, there seems to be an effect on telomere clustering. Is this APB/PML independent? Suggest examining APBs in other cell lines and verification of clustering effects.

Re: examining APBs in other cell lines and verification of clustering effects.

We show that the number of APB per cell does not change after 24 hours of ponatinib treatment in SAOS-2 cells and now also in CAL72 cells (**new data - Supplementary Figure 3a**). The telomere clustering shown in Fig 2J is after 48 hours of treatment. We have now verified that this clustering also happens at 24 hours post-treatment (**new data- Supplementary Figure 4d**), when no change in APB number was detected.

Re: Is this APB/PML independent?

While the number of APB does not change in our experiments, indicating no de novo assembly of APBs, the APBs may have become larger in size after ponatinib treatment. Such enlargement of APBs may result in telomere clustering (Draskovic et al, [13]). Moreover, these large telomeric foci were associated with markers of replicative stress (Fig. 2i and j, same experiments analyzed for telomere clustering and pS33 RPA association) or DNA damage (Fig. 2e), usually detected in the APBs of ALT cells.

To examine whether the effects of ponatinib is independent of APB/PML, we generated PML-deficient SAOS-2 cells and assessed whether ponatinib still increases telomeric C-circle levels. Ponatinib failed to induce aberrant levels of telomeric C-circles in PML-deficient cells (**new data - Supplementary Figure 2e-f**), and these cells were more resistant to ponatinib in clonogenic assays (**new data - Supplementary Figure 2g**). These results indicate that the effects of ponatinib on ALT activity depends at least partly on the presence of APBs.

c. The experiments to assess DNA nicks and gaps are interesting. Recent work from the Cantor lab has applied S1 nuclease instead of ExoIII to detect gaps in newly replicated DNA and this approach seems to be superior to what is presented here. It would be optimal to reassess the gap hypothesis to support the authors conclusions.

The S1 nuclease assay mentioned by the reviewer allows the detection of ssDNA gaps associated with replication stress. The assay is based on labeling ongoing replicating DNA/fork with IdU and CldU followed by a S1 nuclease treatment, DNA spreading and IdU and CldU detection. If ssDNA gaps were formed, S1 nuclease treatment will result in shorter CldU tracts (Quinet et al, 2017, methods Enzymol; [14]). Conceptually, by combining this technique with telomeric FISH, this assay could be used to detect gaps in newly replicated telomeres.

The ExoIII assay that we used allows to detect endogenous gaps (resulting from telomeric replication (ongoing or previous) as well as from intrinsic telomeric DNA strand breaks). The rationale is that the presence of these endogenous ssDNA may cause replication fork collapse or regression, leading to C-circle formation and ALT activity (Zhang et al, [3]).

Therefore, we think that the S1 nuclease-based assay may not be adequate in the context of ALT and endogenous damage-induced telomeric gaps.

d. What is the mechanism of senescence activation here? It appears that p53 is not required. Is this driven by micronuclei and the cGAS pathway?

We agree with the reviewer that p53 does not seem to be required since senescence activation was particularly evident in both SAOS-2 (p53 null) and CAL72 (p53 WT) cells.

We have assessed whether the cGAS-STING pathway is activated after ponatinib treatment. The presence of cytosolic DNA can activate this pathway, leading to activation of TANK-binding kinase 1 (TBK1) and IRF3 and increased transcription of type I interferon genes (Motwani et al, Nat Rev Genet 2019; [15]). We did not observe an increase in IRF3 activation (phosphorylation at serine 396) nor IRF7 activation (part of another cytosolic DNA response pathway) (**Figure R1**).

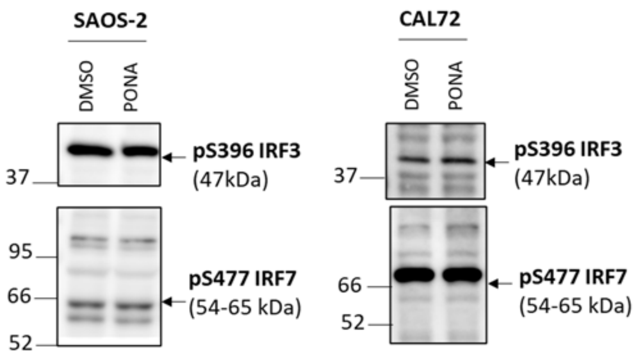

Figure R1: *Western blots showing levels of pS396 IRF3 (antibody (4D4G) #4947, Cell Signaling Technology) and pS477 IRF7 (antibody #PA5-64834, Invitrogen) after 24 hours of treatment with ponatinib in SAOS-2 (at 250 nM) and CAL72 cells (at 125 nM).*

Moreover, the transcript levels of cGAS-STING pathway target genes (including those coding type I interferons) were not induced after ponatinib treatment (**Figure R2**).

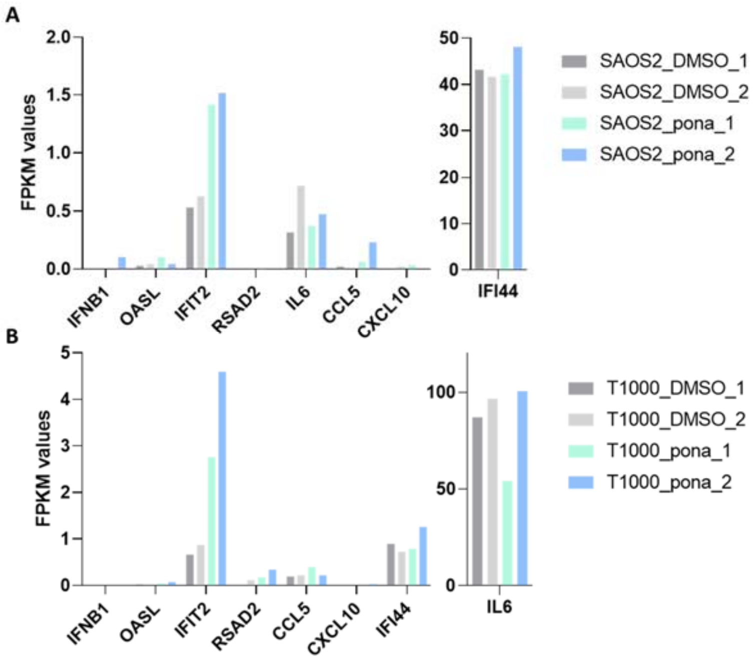

Figure R2: *Graphs showing FPKM transcript values of cGAS-STING pathway target genes in SAOS-2 and T1000 cells treated with either DMSO or ponatinib for 24 hours (from RNA-sequencing data).*

The induction of senescence after ponatinib treatment may be triggered by persistent DNA damage or telomere dysfunction through p53-independent pathways (e.g., p16INK4a/RB [16, 17]), or by other cellular stress produced by ponatinib which may activate other senescence pathways.

e. The effects of Ponatinib in normal, healthy cells are not examined.

We have not examined ponatinib's effects on healthy cells since it is an FDA-approved drug ([18]) and its safety as well as toxicity risks and adverse effects have already been assessed in humans in several clinical trials and real-world clinical data.

Concerning our study, Ponatinib reduced tumor burden in mice without impacting their body weight over 66 days of treatment (now Supplementary Fig 6a). Moreover, mice did not display any treatment-related clinical signs during this study.

3. Are the ALT cell lines used that exhibit sensitivity to Ponatinib ATRX-DAXX mutated? This could be added if there is a bias.

All ALT cell lines used in this study are ATRX deficient. CAL72, U2OS and SAOS-2 were previously shown to lack ATRX protein [19]. We assessed ATRX expression in T1000 LPS cells and have included this Western blot in the manuscript (**new data - Supplementary Figure 1d**) and have described this in line 111.

4. The in vivo studies are important and really going in a strong direction. Yet, the telomere length analysis is not at all convincing. This should be re-examined using Pulse Field Gel Electrophoresis (PFGE). Alternatively, have the authors considered doing TRF analysis with a lower, chronic dose of Ponatinib to explore telomere length alterations.

The telomere length analysis presented in Figure 4d is obtained using Pulse Field Gel Electrophoresis. Telomeres from CAL72 tumors seem to be less heterogeneous than those of CAL72 cells cultured in vitro. Nevertheless, the separation obtained with this PFGE was sufficient to estimate mean telomere length and see statistically significant differences (~ 2Kb on average) between the control and the ponatinib-treated groups.

Re: Alternatively, have the authors considered doing TRF analysis with a lower, chronic dose of Ponatinib to explore telomere length alterations.

We treated SAOS-2 cells with lower doses of ponatinib (50 and 100 nM) for one month. Cells were counted and re-seeded every 7 days and treated again with either ponatinib at 50 or 100 nM or DMSO as a control. Treatment with 50 nM ponatinib did not significantly affect cell viability while 100 nM ponatinib reduced cell number by ~ 20%. After 4 weeks of treatment, cells were collected and TRF analysis was performed (**Figure R3**). At both concentrations, ponatinib did not alter the telomere length distribution. This could be due to the use of low concentrations of ponatinib.

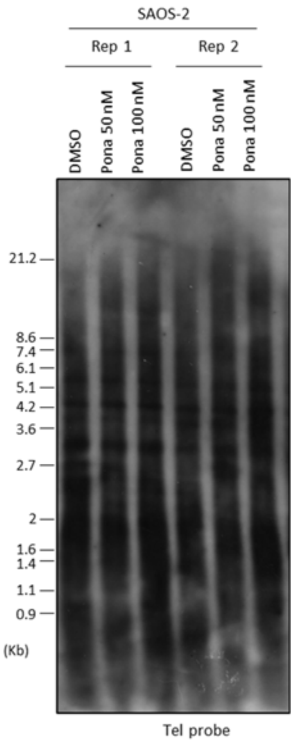

Figure R3: *TRF analysis by PFGE of two replicates of SAOS-2 cells treated weekly with ponatinib at 50 or 100 nM for one month.*

5. In Figure 6A and D, it would be a nice experiment to show that the increases of C-circles/telomere integrity can be suppressed in the JUN KO cells and restored in the complemented cells. Do JUN KO cells show defects in telomere integrity and replication? This would further corroborate the evidence that JUN has an important role in telomere maintenance.

We thank the reviewer for this interesting suggestion. We have assessed the levels of C-circles in JUN-deficient cells after ponatinib treatment. Cells lacking JUN show an increase of C-circles in comparison to control cells (LacZ sg) but do not exhibit a further increase after ponatinib treatment (**new data - Supplementary Figure 8g-h**). This indicates further that JUN is a major player in ponatinib's effect on telomeric C-circles. We have not performed the complementation in these experiments since dual lentivirus-based transduction followed by ponatinib treatment within the span of a short time of this experiment would be quite stressful to the cells.

Re: Do JUN KO cells show defects in telomere integrity and replication?

We quantified the levels of telomeric dysfunction in JUN-depleted SAOS-2 cells. These cells had increased levels of TIFs (telomere-dysfunction induced foci) when compared to control cells (LacZ sg) (**new data - Supplementary Figure 8i**). This was verified in two independent experiments.

6. In Figure 6C, what is the reference for calculation? Is it LacZ in OE-GFP for both sets of experiments? Considering fold change, it looks to me that the effect of OE-JUN is in fact minimal. Correct?

That is correct. The reference for calculation for all the conditions is LacZ sg in OE-GFP. The difference between lacZ sg in OE-JUN and OE-GFP was not statistically significant (p=0.1654) in this experiment, but we did see a clear tendency of reduced C-circles in OE-JUN (please see quantification values below).

OE-GFP		OE-JUN	
LacZ sg	Jun sg1	LacZ sg	Jun sg1
0.737097	3.998127	0.197446	1.2508
1.779147	2.381069	0.311874	2.489875
0.483756	4.812263	0.443496	0.58246

This was more evident in a second set of experiments (please see response to reviewer 4, point 5).

7. The blots in 6G are uninterpretable. This experiment should be repeated and an improved blot presented.

We have repeated the experiment and replaced the blots with new ones with lower exposure time (**new data - Figure 6g**).

8. In Figure 7D, Ponatinib does not induce gH2AX in Saos2 cells. This is a lower dose 125nM instead of 250nM which is used throughout the rest of the story. Why was this the case?

We used a lower concentration to highlight a better synergy between ponatinib and triciribine. We have now repeated the experiment with both concentrations (125 and 250 nM) and included the Western blots in the manuscript (**new data - Figure 7d and Supplementary Figure 11e**).

9. Lastly, the data in the TG20/T16 glioma cell lines are very limited and could be expanded to further show the effects of Ponatinib on ALT.

In the current study, we have illustrated the effect of ponatinib using sarcoma cell models. We only utilized TG20/TG16 cells to showcase that ponatinib may also be efficacious in other ALT-positive tumors. While we agree with the reviewer that an expansion and validation of our work on these cells and other glioma models would be interesting, we would be performing these experiments for subsequent studies given the substantial size of the current manuscript.

<u>Reviewer #4 (Remarks to the Author):</u>

This is a potentially very interesting study that demonstrates effects of a clinically-approved drug on the telomere biology and viability of ALT cancer cells. The effects of this compound on telomere integrity in ALT cells appear to be well-supported by the data. Its effects on tumor growth in vivo are also particularly striking. However, I think there is quite a bit of work remaining to shore up the main conclusions of the study and the mechanism for the observed effects, as outlined below. With these extra data, this study has the potential to be a very important contribution to the field.

We thank the reviewer for their appreciation of our data, and we agree that this study represents a significant contribution to the telomeric field given that it uncovers novel pathways that regulate telomere homeostasis in ALT cells, and that can be clinically targeted as well.

We have addressed the reviewer's concerns below.

Major comments:

1) The introduction (line 63) cites a study showing a tendency for ALT cell lines to be particularly sensitive to ATR inhibitors; however, this conclusion was later brought into question (Deeg et al, Front Oncol 2016; Laroche-Clary et al Scientific Reports 2020). For this reason, it is necessary to apply a great deal of caution when claiming ALT-specific sensitivity to any drug. I would therefore like to see the following additional lines of evidence for ALT-specific sensitivity to ponatinib: i) comparison of a larger panel of ALT vs Tel cell lines than just 4 of each, ii) confirmation of relative drug sensitivities using a second assay type, and iii) comparison of an isogenic pair of cell lines in which ALT has been inhibited in one of them by overexpression of ATRX, as performed in Deeg et al 2016.

Re: i) comparison of a larger panel of ALT vs Tel cell lines than just 4 of each

We share the reviewer's caution about making a claim for ALT-selective therapies, which we do not do here for ponatinib.

Ponatinib is clinically approved for chronic myeloid leukemia and Philadelphia chromosome-positive acute lymphoblastic leukemia (ALL). These tumors do not have a high frequency of the ALT phenotype [20-22] and the efficacy of ponatinib is related to inhibition of the BCR-ABL oncoprotein. Moreover, Ponatinib is a multi-Receptor Tyrosine Kinase inhibitor and inhibits other important proliferative pathways that can be equally important to ALT and telomerase-positive cells.

What we show in our study is that ponatinib may have an enhanced (not selective) activity on ALT cell viability by disrupting ALT, promoting dysfunctional telomeres, and inhibiting ALT-associated telomere synthesis (**new data - Figure 3e-f**). The viability comparisons (SW26 vs SW39; 3 OS ALT vs 3 tel+; T1000 vs T778 and TG20 vs TG16) show an enhanced killing for ALT cells but the separation between groups is not dichotomous. Hence, we do not propose a selective killing for ALT cells and do not rule out that ponatinib can be efficacious against cancer cells independent of their telomere maintenance mechanism. In fact, we find this scenario clinically more interesting in the case of heterogenous tumors having both ALT and telomerase-positive cancer cells. In addition, we do not dismiss that there will be ALT cells that can be inherently resistant to ponatinib.

We have interrogated Depmap portal for cell line response to ponatinib (**Figure R4**) of known ALT cancer cell lines or cell lines reported to have a potential ALT status [33] (https://doi.org/10.1158/1538-7445.AM2015-3768). While not exhaustive, this assessment showed that many ALT-positive cell lines across different cancer types had AUC scores lower than the average AUC score for the tumor subtype, indicating higher sensitivity to ponatinib.

[redacted]

Figure R4: *AUC (Area Under Curve) drug sensitivity scores of dataset GDSC1 downloaded from Depmap portal* (https://depmap.org/portal/)*. Each dot represents one cell line. Some of the known or putative ALT ce ll lines are annotated.*

Re: ii) confirmation of relative drug sensitivities using a second assay type

We assessed the relative sensitivity of our panel of osteosarcoma cell lines to ponatinib by clonogenic assays **(new data - Supplementary Figure 1e-f)**. ALT cell lines showed higher sensitivity to ponatinib in comparison to the telomerase-positive cells, confirming the findings in viability assays.

Re: iii) comparison of an isogenic pair of cell lines in which ALT has been inhibited in one of them by overexpression of ATRX, as performed in Deeg et al 2016.

Instead of using ATRX overexpression to inhibit ALT, we depleted PML in SAOS-2 cells using CRISPR-Cas9. PML sg transduced cells had reduced levels of telomeric C-circles **(new data -Supplementary Figure 2e-f)** and were less sensitive to ponatinib in clonogenic assays, despite their lower basal clonogenic potential **(new data - Supplementary Figure 2g)**. These results indicate that ponatinib's efficacy on ALT cells is at least partially due to their ALT properties.

2) Since the authors showed that an ALT cell line with WT p53 (U2OS) did not experience global DNA damage in response to ponatinib (Fig S2C), the panel of cell lines referred to in my previous comment

should include both p53 WT and p53-deficient TEL cell lines, to determine whether ponatinib sensitivity correlates with p53 status.

The table below shows the status of p53 in the panel of cell lines we used. Except for induction of global DNA damage, sensitivity to ponatinib does not seem to correlate with p53 status.

Cell line	TP53 status	Reference
U2OS	WT	[23]
CAL72	WT	[23]
SAOS-2	null	[24]
MG63	null	[25]
HT161	null	[26]
HOS	mutated	[24]
T1000	$TP53^{WT}/MDM2^{AMP}$	[27]
T778	$TP53^{WT}/MDM2^{AMP}$	[27]
TG20	mutated	[28]
TG16	mutated	[28]

3) The ALT-specificity of the observations would also be strengthened by: i) determining whether knockdown of JUN impacts viability of TEL cell lines, and ii) determining whether the synergistic effect of ponatinib and triciribine extended to other ALT cell lines, and was not observed in TEL cell lines.

Re: i) determining whether knockdown of JUN impacts viability of TEL cell lines

We depleted JUN in telomerase-positive cell lines HOS, HT161 and MG63 and performed clonogenic assays. JUN knockdown reduced clonogenic potential of HT161 but did not affect MG63 and HOS cells **(new data- Supplementary Figure 9d-e**). While JUN depletion impaired survival of ALT cells more significantly, we do expect that the absence of JUN may affect viability of other telomerase-positive cells, given its major role in transcription regulation. We have included these data in the manuscript but did not use it as an argument for ALT-specificity. The specificity of JUN in ALT cells is related to its role at telomeres.

Re: ii) determining whether the synergistic effect of ponatinib and triciribine extended to other ALT cell lines and was not observed in TEL cell lines.

We thank the reviewer for this suggestion. We tested ponatinib and triciribine combination on our ALT vs. telomerase-positive cell lines in MTT viability assays (**new data- Supplementary Figure 12**). The combination was more effective in reducing viability of ALT cells in comparison to telomerase-positive ones, with synergistic or additive effects in many combinations. We have calculated the synergy scores with an updated version of SynergyFinder (www.synergyfinder.org). These results, together with the synergistic effect on DNA damage and telomeric C-circles, suggest that this drug combination may be particularly effective in ALT cancers.

4) It is stated in the Discussion that “ponatinib’s impact on telomeres and DNA damage is specific to ALT cells”, but, except for effects on global gH2AX levels and C-circles (which TEL cells don’t have), there wasn’t any evidence presented for this. Does ponatinib affect levels of TIFs, telomere aberrations or telomere nicks in TEL cells, or in ALT cells other than SAOS-2 (the only cell line used in Fig 2E-J)?

This statement in the Discussion is in the context of comparing the effects of ponatinib to that of FANCM or SMARCAL1 depletion since the latter cause global DNA damage in both ALT and telomerase-positive cells. Additionally, SMARCAL1 depletion can cause telomeric damage in telomerase-positive cells [29].

We have now shown that ponatinib increases levels of TIFs in another ALT cell line (CAL72) (**new data - Supplementary Figure 3d**) but does not induce them in two telomerase-positive cells (HT161 and HOS) (**new data - Supplementary Figure 3e**).

5) A great deal of experimental effort was invested in determining that Abl1 mediates phosphorylation of JUN on several sites (Y170, S63, T91/93) via JNK kinases (for the latter two sites), but it's not clear to me that any of these phosphorylation events have anything to do with effects on ALT cell telomeres or viability, since a JNK inhibitor did not affect levels of C-circles (Fig S6L). This should be possible to test, using a rescue experiment like that in Figure 6C, but with phosphodeficient or phosphomimetic versions of JUN at each of these sites.

We deciphered the signalling mechanisms to identify the targets and mode of action of ponatinib on ALT. Ponatinib, by inhibiting ABL1 activity, reduces phosphorylation levels of several phosphorylation sites (S63, S73, T91 and/or T93) that regulate the protein stability of JUN. We show that ponatinib reduces these phosphorylations at early time points (Figure 6F), and total levels of JUN are subsequently reduced (Supplementary Figures S7 and S9C). This is stated in the text in lines 267-269: 'Loss of phosphorylation at these sites occurred as early as 30 min after treatment with ponatinib, potentially impacting the stability of JUN and subsequently leading to its degradation by 24 hours'.

We have also tried to explore the implication of single phosphorylations in regulating telomeric C-circles. We cloned the phosphodeficient JUN mutants into lentiviral overexpressing vectors and transduced SAOS-2 cells with either WT or single site-phosphodeficient forms of JUN, and checked for effects on C-circles (**Figure R5**). Phosphomutant JUN T91/93A partially restored levels of telomeric C-circles in comparison to JUN WT, but this effect could be attributed to the lower stability and levels of JUN expressed in this mutant. None of the other single mutants restored levels of C-circles.

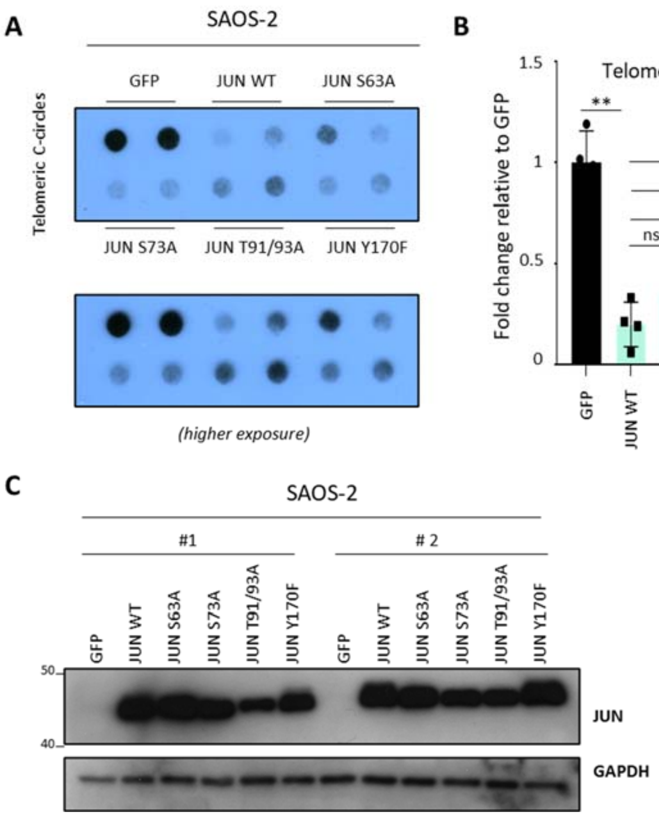

__Figure R5:__ (A) Representative blot of telomeric C-circle assay on SAOS-2 cells overexpressing either GFP, JUN WT or phosphomutant forms of JUN (S63A, S73A, T91/93A, Y170F). (B) Relative quantification of the C-circle assays on 4 replicates. (**p<0.01, *p<0.05, ns=not significant, as determined by paired t-test). (C) Western blot showing expression of JUN in the cell lines used in panels A and B.

Hence at this point, we propose that it is the decrease in JUN protein levels that affects ALT telomeres and viability, which is indicated by our experiments involving genetic depletion of JUN. However, we do not exclude the possibility that these phosphorylations can also regulate JUN's activity, given the results of JUN interactome after ponatinib treatment for 3 hours (at which phosphorylation but not total protein levels are reduced).

6) An effect of JUN on C-circle levels is demonstrated, but it's not clear if JUN affects any of the other markers of telomere integrity shown in Figs 2 and 3 (TIFs, telomere aberrations, telomeric nicks/gaps). The overall involvement of JUN in cellular viability via telomeres would be strengthened by measurement of at least one or two of these parameters after JUN knockdown.

We quantified the levels of telomeric dysfunction in JUN-depleted SAOS-2 cells. These cells had increased levels of TIFs (telomere-dysfunction induced foci) when compared to control cells (LacZ sg) (**new data - Supplementary Figure 8i**). This was verified in two independent experiments.

7) By the end of the paper, the authors conclude that it is JUN's interaction with several DNA repair factors that is likely to be the cause of its effects on ALT telomeres, but there is no direct evidence for this. Does depletion of any of the identified interactors (lines 296, 297) recapitulate the effects of ponatinib or JUN knockdown on markers of telomere integrity in ALT cells?

[Redacted]

[Redacted]

8) The effects of ponatinib on CAL72 cells was different in vitro and in vivo, and this difference was not addressed in the text. Ponatinib caused an increase in t-circles in vitro but a decrease in vivo; it had no apparent effect on telomere length in vitro, but resulted in shorter telomeres in vivo. What are some possible reasons for these differences?

One possible hypothesis that we put forward (lines 196-198) is that cells with higher ALT activity we targeted during the long treatment and the resultant remaining tumors were formed by cells with lower ALT activity. While we are looking at immediate short-term effects in vitro, the significantly long period of treatment and the selection pressure in vivo allowed us to see the final outcome of ponatinib on ALT tumors, especially regarding telomeric C-circle formation. On the other hand, the telomere shortening in vivo confirms the in vitro results concerning the inhibition of telomere synthesis. However, with this experimental set up, we cannot determine whether this shortening is an active loss of telomeres or a selection of cells with shorter telomeres and lower ALT activity.

Minor comments:

i. Figure 1: for the viability assays, it is essential to specify in the figure legend the length of time for which cells were treated, in order to distinguish viability effects resulting from gradual telomere shortening (which would be expected to take time to manifest) from more rapid effects.

The treatment for these assays was for 72 hours. We have added this to the legend. The effects on viability are not due to telomere shortening, but we rather think it is partly caused by excessive telomeric dysfunction and aberrant ALT activity.

ii. Figure 1B: it's not clear if the data in the right graph represent one ALT cell line and one TEL cell line (if so, which ones?), or a compilation of data from all the cell lines in the left graph?

The right graphs are a compilation of all the cell lines shown in the left graph, we have clarified this information in the figure legend.

iii. Should the cell line referred to as H161 instead be HT161? (https://web.expasy.org/cellosaurus/CVCL_C834).

Yes, we have corrected the name of the cell line to HT161.

iv. The labelling in Figure 6E is not large enough to read.

We have increased the labelling font for Figure 6e.

v. The right panel of Figure 6G (Y170F JUN) is not very convincing because the background is so dark; this experiment should be repeated.

We have repeated the experiment for both JUN WT and JUN Y170F and replaced the blots with lower exposure time (**new data - Figure 6g**).

References

1. Henson, J.D., et al., *DNA C-circles are specific and quantifiable markers of alternative-lengthening-of-telomeres activity.* Nat Biotechnol, 2009. **27**(12): p. 1181-5.
2. Henson, J.D., et al., *The C-Circle Assay for alternative-lengthening-of-telomeres activity.* Methods, 2017. **114**: p. 74-84.
3. Zhang, T., et al., *Strand break-induced replication fork collapse leads to C-circles, C-overhangs and telomeric recombination.* PLoS Genet, 2019. **15**(2): p. e1007925.
4. Ahmed, S.M., et al., *The chromatin structuring protein HMGA2 influences human subtelomere stability and cancer chemosensitivity.* PLoS One, 2019. **14**(5): p. e0215696.
5. Zhang, J.M., et al., *Alternative Lengthening of Telomeres through Two Distinct Break-Induced Replication Pathways.* Cell Rep, 2019. **26**(4): p. 955-968 e3.
6. Lang, M., et al., *Three-dimensional organization of promyelocytic leukemia nuclear bodies.* J Cell Sci, 2010. **123**(Pt 3): p. 392-400.
7. Cox, J., et al., *Accurate proteome-wide label-free quantification by delayed normalization and maximal peptide ratio extraction, termed MaxLFQ.* Mol Cell Proteomics, 2014. **13**(9): p. 2513-26.
8. Desi, N., et al., *Global analysis of RNA-binding proteins identifies a positive feedback loop between LARP1 and MYC that promotes tumorigenesis.* Cell Mol Life Sci, 2022. **79**(3): p. 147.
9. Hein, M.Y., et al., *A human interactome in three quantitative dimensions organized by stoichiometries and abundances.* Cell, 2015. **163**(3): p. 712-23.
10. Huttlin, E.L., et al., *The BioPlex Network: A Systematic Exploration of the Human Interactome.* Cell, 2015. **162**(2): p. 425-440.
11. Porro, A., et al., *Functional characterization of the TERRA transcriptome at damaged telomeres.* Nat Commun, 2014. **5**: p. 5379.
12. Silva, B., et al., *FANCM limits ALT activity by restricting telomeric replication stress induced by deregulated BLM and R-loops.* Nat Commun, 2019. **10**(1): p. 2253.
13. Draskovic, I., et al., *Probing PML body function in ALT cells reveals spatiotemporal requirements for telomere recombination.* Proc Natl Acad Sci U S A, 2009. **106**(37): p. 15726-31.
14. Quinet, A., et al., *DNA Fiber Analysis: Mind the Gap!* Methods Enzymol, 2017. **591**: p. 55-82.
15. Motwani, M., S. Pesiridis, and K.A. Fitzgerald, *DNA sensing by the cGAS-STING pathway in health and disease.* Nat Rev Genet, 2019. **20**(11): p. 657-674.
16. Beausejour, C.M., et al., *Reversal of human cellular senescence: roles of the p53 and p16 pathways.* EMBO J, 2003. **22**(16): p. 4212-22.
17. Jacobs, J.J. and T. de Lange, *Significant role for p16INK4a in p53-independent telomere-directed senescence.* Curr Biol, 2004. **14**(24): p. 2302-8.
18. Luciano, L., et al., *The multi-tyrosine kinase inhibitor ponatinib for chronic myeloid leukemia: Real-world data.* Eur J Haematol, 2020. **105**(1): p. 3-15.
19. Flynn, R.L., et al., *Alternative lengthening of telomeres renders cancer cells hypersensitive to ATR inhibitors.* Science, 2015. **347**(6219): p. 273-7.
20. Lansdorp, P.M., *Maintenance of telomere length in AML.* Blood Adv, 2017. **1**(25): p. 2467-2472.
21. Ohyashiki, K., et al., *Telomerase activity and cytogenetic changes in chronic myeloid leukemia with disease progression.* Leukemia, 1997. **11**(2): p. 190-4.
22. Samassekou, O., et al., *Presence of alternative lengthening of telomeres associated circular extrachromosome telomere repeats in primary leukemia cells of chronic myeloid leukemia.* J Hematol Oncol, 2013. **6**: p. 26.

23. Bamford, S., et al., *The COSMIC (Catalogue of Somatic Mutations in Cancer) database and website.* Br J Cancer, 2004. **91**(2): p. 355-8.
24. Oshima, Y., et al., *Antitumor effect of adenovirus-mediated p53 family gene transfer on osteosarcoma cell lines.* Cancer Biol Ther, 2007. **6**(7): p. 1058-66.
25. Chandar, N., et al., *Inactivation of p53 gene in human and murine osteosarcoma cells.* Br J Cancer, 1992. **65**(2): p. 208-14.
26. Diller, L., et al., *p53 functions as a cell cycle control protein in osteosarcomas.* Mol Cell Biol, 1990. **10**(11): p. 5772-81.
27. Muret, J., et al., *Attenuation of soft-tissue sarcomas resistance to the cytotoxic action of TNF-alpha by restoring p53 function.* PLoS One, 2012. **7**(6): p. e38808.
28. Silvestre, D.C., et al., *Alternative lengthening of telomeres in human glioma stem cells.* Stem Cells, 2011. **29**(3): p. 440-51.
29. Poole, L.A., et al., *SMARCAL1 maintains telomere integrity during DNA replication.* Proc Natl Acad Sci U S A, 2015. **112**(48): p. 14864-9.

[Redacted]

.

33. Andrei G. Malykh; Jonathan R. Doyle; Fang Tian; Joyce HY Lee; Jeremy D. Henson; Roger R. Reddel. Abstract 3768: Identification of new alternative lengthening of telomeres (ALT)-positive cancer cell lines using the C-circle assay. Cancer Res (2015) 75 (15_Supplement): 3768. (https://doi.org/10.1158/1538-7445.AM2015-3768).

Reviewers' comments:

Reviewer #1 (Remarks to the Author):

The authors have revised the manuscript extensively. In spite of the fact that the authors addressed most of the concerns, the Exo III assay (Fig3a-b) is still not convincing. In the original paper (Zhang et al), the ssDNA break/gap was well demonstrated using in gel-hybridization. However, the author utilized membranes to detect the gaps/breaks in ssDNA after crosslinking the membranes. There is a significant difference between the original EXOIII assay and the author's assay. In the event that the author is not able to perform the assay using in-gel hybridization, Fig3a-b should be omitted from the manuscript. The evidence presented in Figure 2i is sufficient to support the the author's conclusion that Ponatinib treatment produces ssDNAs at telomeres.

Reviewer #3 (Remarks to the Author):

While the authors have addressed some of the points raised, it was disappointing that many have not been adequately addressed, with some remaining entirely unanswered (eg. effects in healthy cells, complementation, telomere length analysis in tumors remain unconvincing and over-interpreted). Other aspects that have been addressed remain somewhat unclear. For instance, why do the cells activate senescence and how if they lack p53? Also, what impact Jun has on telomeres in ALT cells remains completely unclear (aside from a modest effect on TIFs). Unfortunately, this is all a bit unsatisfactory. I sincerely hoped that the revised manuscript and clarifying the issues raised would provide greater insights and assurances, such that it would then sail through to publication. However, this reviewer can't help but feel disappointed with the revision.

Reviewer #4 (Remarks to the Author):

The authors have satisfactorily addressed all of my concerns.

Response to the Reviewers' comments:

<u>Reviewer #1 (Remarks to the Author):</u>

The authors have revised the manuscript extensively. In spite of the fact that the authors addressed most of the concerns, the Exo III assay (Fig3a-b) is still not convincing. In the original paper (Zhang et al), the ssDNA break/gap was well demonstrated using in gel-hybridization. However, the author utilized membranes to detect the gaps/breaks in ssDNA after crosslinking the membranes. There is a significant difference between the original EXOIII assay and the author's assay. In the event that the author is not able to perform the assay using in-gel hybridization, Fig3a-b should be omitted from the manuscript. The evidence presented in Figure 2i is sufficient to support the the author's conclusion that Ponatinib treatment produces ssDNAs at telomeres.

We thank the reviewer for the appreciation of the extensive revision of the manuscript. We agree with the reviewer that the results in Figure 2i already support the ponatinib-induced increase of ssDNAs at telomeres. Hence, we have omitted the ExoIII assays for telomeric ssDNA from the manuscript (previously Fig 3a-b).

<u>Reviewer #3 (Remarks to the Author):</u>

While the authors have addressed some of the points raised, it was disappointing that many have not been adequately addressed, with some remaining entirely unanswered (eg. effects in healthy cells, complementation, telomere length analysis in tumors remain unconvincing and over-interpreted). Other aspects that have been addressed remain somewhat unclear. For instance, why do the cells activate senescence and how if they lack p53? Also, what impact Jun has on telomeres in ALT cells remains completely unclear (aside from a modest effect on TIFs). Unfortunately, this is all a bit unsatisfactory. I sincerely hoped that the revised manuscript and clarifying the issues raised would provide greater insights and assurances, such that it would then sail through to publication. However, this reviewer can't help but feel disappointed with the revision.

We regret to hear that our efforts to answer the reviewer’s previous questions were not satisfactory, especially since we had genuinely aimed to address all concerns, including the role of APBs in response to ponatinib, the cGAS pathway in ponatinib-induced senescence, the ATRX status in ALT cells, the TRF measurements *in vitro* on cells upon long-term treatment with low doses of ponatinib, the effect of ponatinib in JUN KO cells, the effect of JUN overexpression, and repeating the experiment in Figure 6g and testing two concentrations of ponatinib with triciribine for ϒH2AX induction.

We now provide additional data to address the remaining concerns.

- <u>Effect of ponatinib on normal healthy cells</u>

Ponatinib is an FDA-approved drug and is already in clinical use [1] indicating that the effects of ponatinib on tumor cells outweigh any side toxicity on non-transformed (healthy) cells. As mentioned in our initial response, a solid indication of the effect of ponatinib on normal cells is the preclinical data in mice. We show that ponatinib reduced tumor size without affecting

the body weight of the mice (representing here the normal healthy body) (Supplementary Fig. 6a, shown below) nor altering any obvious physiological functions. Such preclinical evidence proves the specific anti-tumor potential of ponatinib compared to somatic cells and is in agreement with the FDA-approval that ponatinib is likely safe to use.

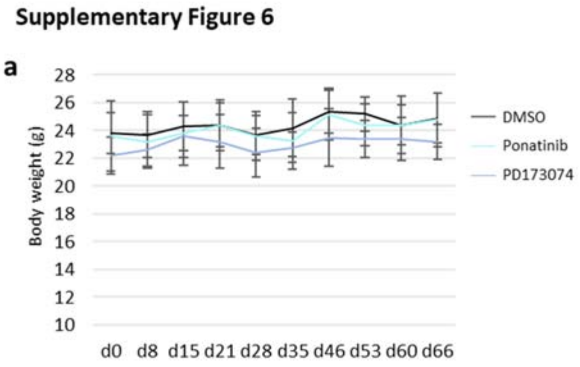

Concerning the effect of ponatinib on telomeres, we had shown that, in contrast to ALT-positive cancer cells, ponatinib does not induce telomeric dysfunction in telomerase-positive cancer cells (Supplementary Fig. 3e). To verify whether ponatinib affects telomeres of normal human cells, we treated IMR90 (foetal lung fibroblasts) with ponatinib and assessed its effect on viability and telomere damage. The cytotoxicity of ponatinib on IMR90 cells was in a similar range to telomerase-positive cells, with an IC50 value of ~ 1µM (**new data-Supplementary Fig. 1e**). Importantly, challenged IMR90 cells did neither produce telomeric C-circles (**new data-Supplementary Fig. 2d**) nor exhibit an increase in damaged telomeres/TIFs (assessed by 53BP1 and telomere staining) (**new data-Supplementary Fig. 3f**).

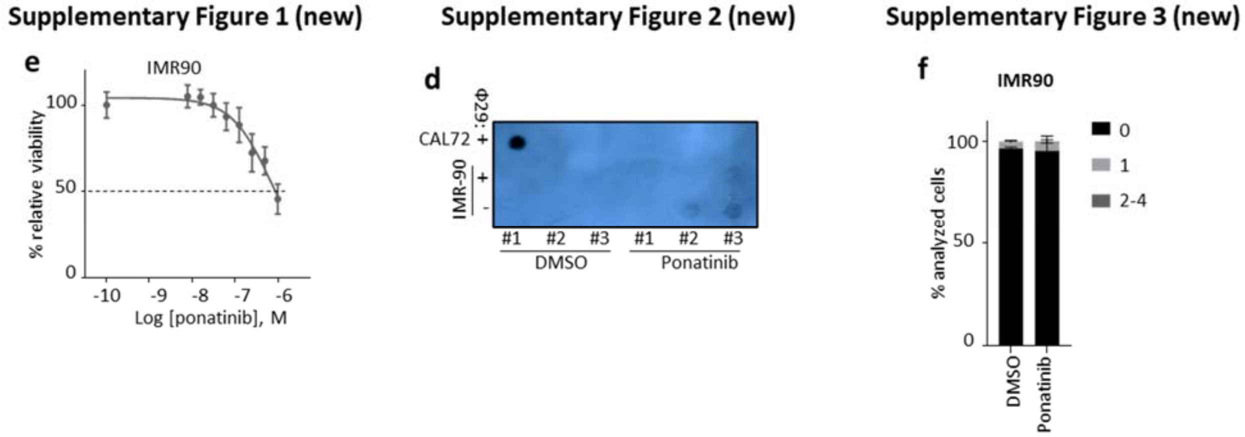

- <u>Ponatinib effect on JUN KO cells (+complementation) + impact of JUN on telomeres</u>

We appreciated this important previous suggestion from the reviewer to treat JUN-depleted cells with ponatinib and assess levels of telomeric C-circles (Supplementary Figures 8g-h, shown below). These results further strengthened the central role of JUN in ponatinib's action on telomeres.

Supplementary Figure 8

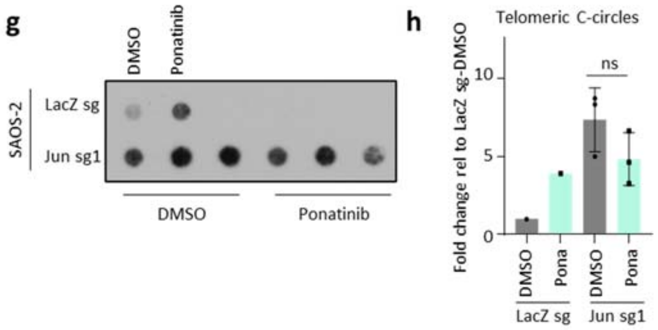

The reviewer further suggested performing a complementation assay in this set of experiments. We did not perform this experiment in the first round of revision because the cells displayed a substantially stressed morphology after dual lentiviral transduction, and we speculated that treating the cells in addition with ponatinib will very likely give inaccurate results. We have now generated these cell lines again and as observed earlier, dual-transduced control (GFP+ LacZ sg) as well as complemented (JUN-OE + jun sg1) cells exhibit a stressed morphology, which was even more evident after ponatinib treatment (Figure R1a). We nevertheless performed the C-circle assays on these cells. Ponatinib did not induce C-circles even in the GFP-LacZ sg cells used as a positive control (Fig. R1b), confirming our initial skepticism in using these cells. We infer that this is caused by an unphysiological cell state that interferes with drug response.

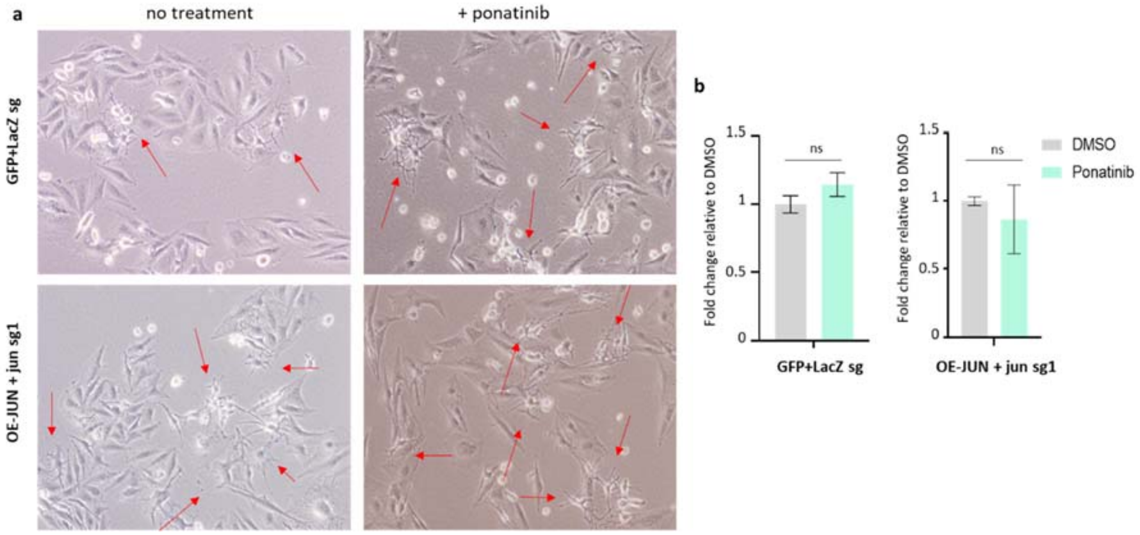

Figure R1: *(a) Representative images of SAOS-2 cells transduced with either lacZ sg and GFP-OE or jun sg1 and JUN WT-OE. Red arrows point to cells with stressed morphology. Cells were treated with ponatinib (250 nM) for 72 hours. (b) Relative quantification of telomeric C-circles in cells generated in (a). (n=6, ns=not significant, as determined by unpaired t-test).*

However, we would like to draw the reviewer's attention to our earlier complementation approach, which demonstrates JUN's function in response to ponatinib (Figure 6c, shown below). Here, cells lacking endogenous JUN but expressing exogenous JUN behave similarly to control WT cells in terms of C-circle levels. In our opinion, this experiment, together with Supplementary Figures 8g-h, provides convincing enough evidence of JUN's impact on telomeres in response to ponatinib treatment.

Figure 6

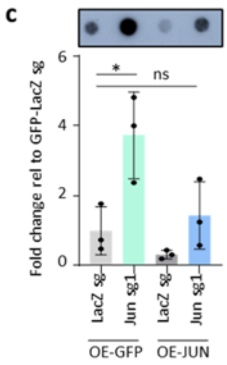

In addition, we had also shown that JUN-deficient cells have increased levels of telomere dysfunction (Supplementary Figure 8i). While the reviewer considers this increase (2-fold) moderate, this effect is similar to the effect of ponatinib treatment (Figure 2e). It is important to highlight that the significance of such fold changes in TIFs have been reported and well supported in different contexts, such as the increase seen after depletion of important regulators of ALT such as POLD3 (Dilley et al, Nature, 2016 [2]), RAD51AP1 (Barroso-Gonzalez et al, Mol Cell, 2019 [3]), or HMBOX1 (Feng et al, J Cell Sci, 2013 [4]), to cite a few.

It is essential to note here that experiments are performed on JUN-deficient cells around 7-10 days post-lentiviral transduction, a time at which we are sure that JUN is depleted, and cell proliferation begins to be compromised. We do not carry experiments beyond this time interval because we are not certain that surviving cells will not undergo adaptations to counteract loss of JUN. Hence, we find that the telomeric C-circles, which can be passed to daughter cells after cell division, are a sensitive gauge of perturbance of ALT during the process of loss of JUN.

[Redacted]

[Redacted]

In sum, these results clearly indicate that JUN impacts ALT activity.

- Telomere length analysis in vivo

The reviewer initially pointed out that the TRF *in vivo* should be done using Pulse Field Gel Electrophoresis (PFGE). We explained in our response that this had already been the case and that these tumors did not have the same telomere length heterogeneity as cells cultured *in vitro*, or what is generally expected and known for ALT cell lines *in vitro*. In fact, we had compared this when setting up the conditions for the TRF of the tumors (please see Figure R3a where we ran samples from CAL72 tumors, together with extracts from CAL72 cells *in vitro*).

Contrary to the heterogeneous distribution typically found in ALT cells in vitro, these tumors have a narrower distribution of telomeres (Fig. R3a), probably due to a selection pressure in vivo. Since this is the first report of CAL72 xenografts analysed by TRF, we could not compare our results with previously published TRFs, especially given that established ALT xenograft models in general are exceedingly rare. However, we would like to stress that our PFGE-based TRF analysis follows established telomere lengths analysis protocols, i.e., widely accepted field standards.

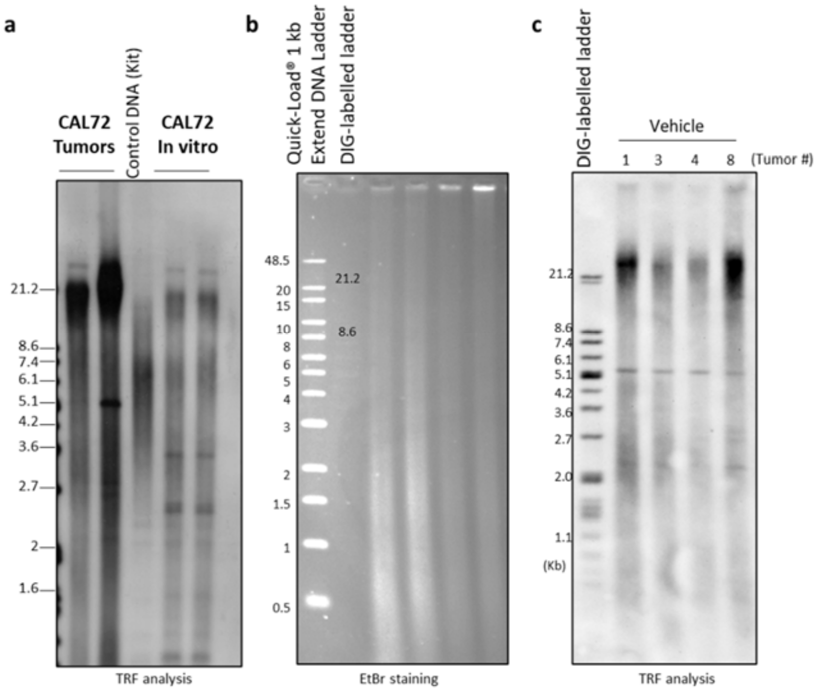

<u>Figure R3:</u> *(a) TRF assay comparing telomere length distribution in CAL72 tumors vs. CAL72 cells in vitro. (b-c) TRF assay on CAL72 tumors from mice treated with vehicle control (same samples as ones used in Figure 4d). Ethidium Bromide staining in (b) shows a 1kb extended ladder with a clear separation of molecules at least up to 48.5 Kb. Telomere probing after Southern transfer is shown in (c).*

Unfortunately, we did not have DNA samples left from tumors in the ponatinib group, and while we tried to extract DNA from frozen remaining tissue, the DNA quality and integrity were not acceptable for many samples for downstream analysis. Nevertheless, to show that the telomere distribution seen in these tumors is not due to an improper separation of high molecular weight DNA, we repeated the PFGE using some vehicle-treated tumors based on the same conditions as before (Fig. R3b-c). Here, we added a 1kb-extend DNA ladder (NEB) and show that at least molecules up to 48.5 Kb can clearly be resolved by our PFGE conditions.

To prevent overinterpretation of our conclusion concerning telomere length distributions comparisons between control and ponatinib-treated tumors, we simply propose now that residual tumors had lower levels of C-circles and slightly shorter telomeres, potentially indicating a perturbance of ALT activity *in vivo* or a drug selection pressure of cells with lower ALT activity (**lines 199-205**, shown below). The effects of ponatinib on telomeric C-circles (Figure 4c) are already a strong result showing ponatinib's impact on the ALT phenotype of these tumors.

Lines 199-205: "*By Telomere Restriction Fragment (TRF) analysis, we noticed that remaining tumors from mice treated with ponatinib had a slightly shorter average telomere length when compared to tumors from the control group (Fig. 4d, Supplementary Fig. 6b). These results potentially indicate that cells with higher ALT activity were particularly sensitive to ponatinib during initial treatments and were inhibited for in vivo growth or that the remaining tumors*

have a lower ALT potential. We conclude that ponatinib is potent at altering the telomeric homeostasis of ALT cells both in vitro and in vivo."

We also edited the discussion and removed the conclusion pointing to ponatinib reducing telomere synthesis *in vivo*: ''We showed that ALT cells treated with ponatinib accumulated telomeric replicative stress but were no longer able to engage in either productive telomere synthesis or extension mechanisms in vitro.~~, as well as in vivo.~~" (**lines 362-364**).

- Mechanism of senescence. Is it driven by cGAS pathway? Why do the cells activate senescence and how if they lack p53?

-Is it driven by cGAS pathway?

The hypothesis that an accumulation of telomeric C-circles and micronuclei activates the cGAS pathway leading to senescence would have been a neat model to explain the ponatinib-induced senescence. However, in the first round of revision, we tested this and found that the cGAS pathway was not activated in ponatinib-treated cells. This result was not surprising given that ALT cells have been reported to be defective in sensing cytosolic DNA and unable to activate the cGAS-STING pathway (Chen et al, Nat Struct Mol Biol, 2017 [5]).

-Mechanism of senescence + Requirement of p53

We agree with the reviewer that p53 does not seem to be required for ponatinib's-induced senescence.

The senescence observed after ponatinib treatment is very likely to be distinct from the replicative senescence that happens after extensive culture and telomere shortening. We think this is more of an accelerated senescence triggered by DNA damage, genotoxic or oxidative stress, and is frequently seen in cells treated with anti-cancer agents ([6] [7]; [8]; [9]). Such senescent phenotype can be induced in cancer cells lacking p53, therefore p53 seems to play a limited role in this therapy-induced senescence ([10] [11]). For instance, DNA-damaging agents (camptothecin [12], cisplatin [7], doxorubicin [7], diaziquone [13], etoposide [7]) as well as gamma irradiation [8] induce senescence in p53-deficient cells, including SAOS-2 cells [7].

Moreover, damaged, uncapped, or dysfunctional telomeres (for instance after TRF2 depletion or inhibition ([14] [15] [16]) can induce such acute senescence. This can happen through p53-dependent or p53-independent pathways ([17] [18] [14]).

We assessed the main senescence pathways that could be induced after ponatinib treatment and tested the canonical p53/p21 and p16/Rb pathways, as well as p27 induction and the stress-driven ATF4-dependent pathway [19, 20].

By real-time qPCR, we quantified transcript levels of the cell cycle inhibitors $p16^{INK4a}$, p21, p27, as well as the transcription factor ATF4, in both SAOS-2 (p53 and Rb-deficient [21]) and CAL72 (p53 WT and Rb WT) after 72 hours of ponatinib treatment. Upregulation of these genes drives senescence and can happen through a p53-dependent (e.g., p21) or -independent signalling (e.g., $p16^{INK4a}$, p21, p27, ATF4). In both SAOS-2 and CAL72 cells, only levels of p27

and ATF4 transcripts were significantly up-regulated after ponatinib treatment (**new data-Supplementary Fig. 4f**).

Supplementary Figure 4 (new)

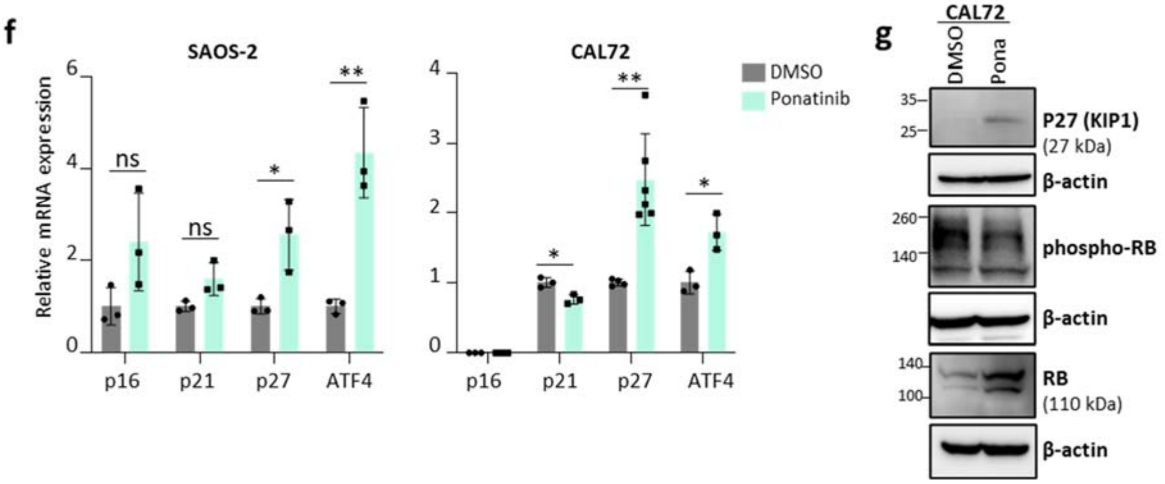

We further confirmed the induction of p27 by Western blot in CAL72 cells (**new data-Supplementary Fig. 4g**). Moreover, in CAL72 cells, an induction of Rb and a reduction of relative phosphorylation of Rb were detected by Western blot after 72 hours of treatment with ponatinib (**new data-Supplementary Fig. 4g).**

The up-regulation of ATF4, as well as an endoplasmic reticulum stress-inducible protein FAM129A/Niban [22] by ponatinib was also seen by RNA-seq in both SAOS-2 and T1000 ALT cells after 24 hours of treatment (Supplementary Figure 7b) and confirmed by Western blot (**new data-Supplementary Figure 7c**).

Supplementary Figure 7 (new)

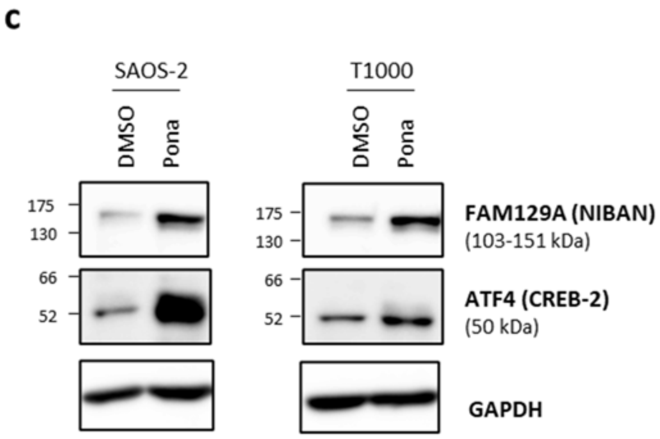

Altogether, while it is not straightforward to decipher the precise mechanism of senescence implicated here, these results indicate that ponatinib may activate several distinct senescence pathways, including a p27-Rb axis, an ATF4- and/or p27-dependent pathway. Up-regulation of ATF4 and p27 can drive senescence in a p53-independent manner [23], and p27 has been reported to induce senescence in the absence of Rb, including in SAOS-2 cells [24, 25]. While

ATF4 can modulate expression of p27 [26], it is not clear whether they are effectors in the same senescence pathway.

In parallel, we cannot rule out that other factors might be contributing to the onset of ponatinib-induced senescence. For instance, the loss of DNA repair gene expression was described as one hallmark and cause of several types of cellular senescence [27]. Given that ponatinib reduced expression of DNA repair and replication genes (Figure 5e), this might be an additional concomitant cause of senescence induction.

In sum, ponatinib-treated cells could be undergoing senescence due to several stimuli that drive acute cellular senescence: dysfunctional telomeres (e.g., in SAOS-2 and CAL72 cells), DNA damage (e.g., in SAOS-2 and T1000 cells) and potential other drug-induced cellular stress (e.g., endoplasmic reticulum stress, genotoxic/oxidative stress), and could, in different cellular and genetic contexts, be driven by several molecular pathways, including ATF4 and p27.

Lastly, it is important to realize that senescence (seen in less than 10% of treated cells) is one but not the only way through which ponatinib affects cell viability.

- <u>Heterogeneity of some results</u>

Regarding the heterogeneity in the outcome of some experiments, as mentioned in our initial response, these results are based on genuine biological replicates. Moreover, the major findings were reproduced at least once more during revision. Rather than raising concerns, these data robustly demonstrate the reproducibility of ponatinib's effects on ALT, especially since these were seen across a variety of assays:

- effects on viability (assessed by 3 assays: proliferation, clonogenic assays and *in vivo* xenograft experiments).

- various effects on telomeres and ALT characteristics (assessed by at least 10 assays: telomeric C-circles (all cell lines), DNA damage by western blots (T1000 and SAOS-2) and TIF (CAL72 and T1000) assays, telomere aberrations by FISH (SAOS-2), telomere transcription by qRT-PCR (SAOS-2), replication stress by micronuclei analysis, pS33 RPA and telomere co-localizations by FISH-IF (SAOS-2), telomere synthesis by BrdU pull-downs (U2OS) and ATSA (U2OS and SAOS-2)).

Given the concerns with the outliers in Figure 1d, we have repeated once more the C-circle assay for CAL72 and T1000 cells and replaced the outlier values with the new results (as shown below). The new graph confirms our previous findings and demonstrates that despite the heterogeneity in the C-circle assay analysis, the outcome is robust and reproducible.

Figure 1 **Figure 1 (new)**

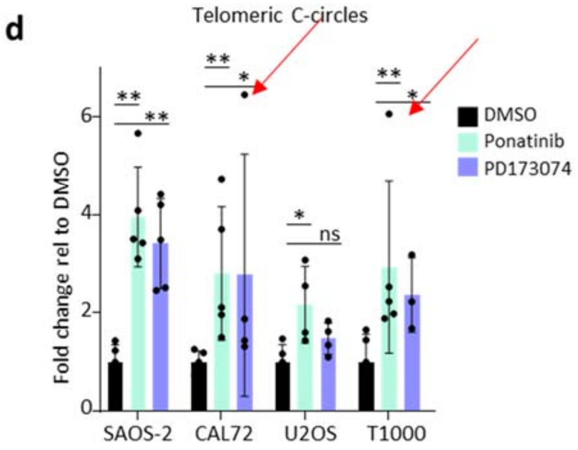

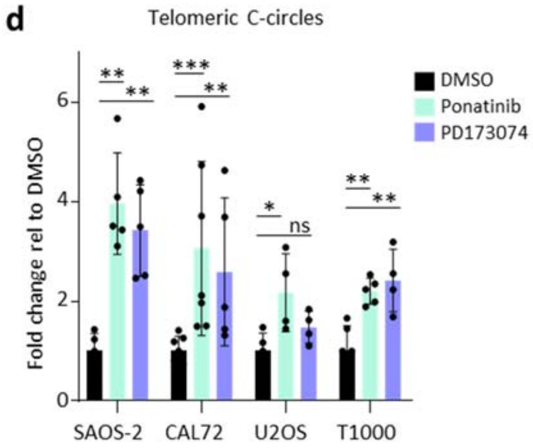

Importantly, our choice of using telomeric C-circles as a marker of ALT activity is based on the established findings in the field that C-circles are robust bona fide quantifiable markers of ALT activity, both in research and in clinical settings. Indeed, being rapidly responsive to changes in ALT activity, telomeric C-circles have been used as a tool for detection of ALT [28, 29], and for identification of key regulators of ALT mechanisms [2, 3, 30]. Our results show that while ALT cells had different response potential to ponatinib (e.g., DNA damage, senescence, etc..); they all showed an increase in their telomeric C-circles upon treatment with ponatinib. This indicates that the use of telomeric C-circles is more reliable and especially more sensitive in detecting ALT phenotypic changes compared to analyzing immuno- and FISH-staining experiments.

We did not see an increase in APB numbers. However, importantly, the ALT-associated telomere synthesis which occurs in APBs was significantly and rapidly inhibited after ponatinib treatment (Figure 3), indicating an impact on functional APBs, and pointing out that the increase of C-circles that we observe may stem from unresolved damage or replication defects at telomeres rather than from an increase of productive ALT activity, for which one possible scenario would be a concomitant increase in the number of APBs. We have included this in the discussion in **lines 373-377**.

Reviewer #4 (Remarks to the Author):

The authors have satisfactorily addressed all of my concerns.

We thank the reviewer for their feed-back and we are happy to have addressed all their concerns.

References

1. Luciano, L., et al., *The multi-tyrosine kinase inhibitor ponatinib for chronic myeloid leukemia: Real-world data.* Eur J Haematol, 2020. **105**(1): p. 3-15.
2. Dilley, R.L., et al., *Break-induced telomere synthesis underlies alternative telomere maintenance.* Nature, 2016. **539**(7627): p. 54-58.
3. Barroso-Gonzalez, J., et al., *RAD51AP1 Is an Essential Mediator of Alternative Lengthening of Telomeres.* Mol Cell, 2019. **76**(1): p. 11-26 e7.
4. Feng, X., et al., *The telomere-associated homeobox-containing protein TAH1/HMBOX1 participates in telomere maintenance in ALT cells.* J Cell Sci, 2013. **126**(Pt 17): p. 3982-9.
5. Chen, Y.A., et al., *Extrachromosomal telomere repeat DNA is linked to ALT development via cGAS-STING DNA sensing pathway.* Nat Struct Mol Biol, 2017. **24**(12): p. 1124-1131.
6. Ewald, J.A., et al., *Therapy-induced senescence in cancer.* J Natl Cancer Inst, 2010. **102**(20): p. 1536-46.
7. Chang, B.D., et al., *A senescence-like phenotype distinguishes tumor cells that undergo terminal proliferation arrest after exposure to anticancer agents.* Cancer Res, 1999. **59**(15): p. 3761-7.
8. Gewirtz, D.A., S.E. Holt, and L.W. Elmore, *Accelerated senescence: an emerging role in tumor cell response to chemotherapy and radiation.* Biochem Pharmacol, 2008. **76**(8): p. 947-57.
9. Roberson, R.S., et al., *Escape from therapy-induced accelerated cellular senescence in p53-null lung cancer cells and in human lung cancers.* Cancer Res, 2005. **65**(7): p. 2795-803.
10. Chang, B.D., et al., *Molecular determinants of terminal growth arrest induced in tumor cells by a chemotherapeutic agent.* Proc Natl Acad Sci U S A, 2002. **99**(1): p. 389-94.
11. Chang, B.D., et al., *Role of p53 and p21waf1/cip1 in senescence-like terminal proliferation arrest induced in human tumor cells by chemotherapeutic drugs.* Oncogene, 1999. **18**(34): p. 4808-18.
12. Han, Z., et al., *Role of p21 in apoptosis and senescence of human colon cancer cells treated with camptothecin.* J Biol Chem, 2002. **277**(19): p. 17154-60.
13. Ewald, J.A., et al., *A high-throughput method to identify novel senescence-inducing compounds.* J Biomol Screen, 2009. **14**(7): p. 853-8.
14. Smogorzewska, A. and T. de Lange, *Different telomere damage signaling pathways in human and mouse cells.* EMBO J, 2002. **21**(16): p. 4338-48.
15. Takai, H., A. Smogorzewska, and T. de Lange, *DNA damage foci at dysfunctional telomeres.* Curr Biol, 2003. **13**(17): p. 1549-56.
16. Wu, L., et al., *Pot1 deficiency initiates DNA damage checkpoint activation and aberrant homologous recombination at telomeres.* Cell, 2006. **126**(1): p. 49-62.
17. Beausejour, C.M., et al., *Reversal of human cellular senescence: roles of the p53 and p16 pathways.* EMBO J, 2003. **22**(16): p. 4212-22.
18. Jacobs, J.J. and T. de Lange, *Significant role for p16INK4a in p53-independent telomere-directed senescence.* Curr Biol, 2004. **14**(24): p. 2302-8.
19. Abbadie, C. and O. Pluquet, *Unfolded Protein Response (UPR) Controls Major Senescence Hallmarks.* Trends Biochem Sci, 2020. **45**(5): p. 371-374.
20. Pluquet, O., A. Pourtier, and C. Abbadie, *The unfolded protein response and cellular senescence. A review in the theme: cellular mechanisms of endoplasmic reticulum stress signaling in health and disease.* Am J Physiol Cell Physiol, 2015. **308**(6): p. C415-25.
21. Zoumpoulidou, G., et al., *Therapeutic vulnerability to PARP1,2 inhibition in RB1-mutant osteosarcoma.* Nat Commun, 2021. **12**(1): p. 7064.
22. Sun, G.D., et al., *The endoplasmic reticulum stress-inducible protein Niban regulates eIF2alpha and S6K1/4E-BP1 phosphorylation.* Biochem Biophys Res Commun, 2007. **360**(1): p. 181-7.
23. Lin, H.K., et al., *Skp2 targeting suppresses tumorigenesis by Arf-p53-independent cellular senescence.* Nature, 2010. **464**(7287): p. 374-9.

24. Alexander, K. and P.W. Hinds, *Requirement for p27(KIP1) in retinoblastoma protein-mediated senescence.* Mol Cell Biol, 2001. **21**(11): p. 3616-31.
25. Collado, M., et al., *Inhibition of the phosphoinositide 3-kinase pathway induces a senescence-like arrest mediated by p27Kip1.* J Biol Chem, 2000. **275**(29): p. 21960-8.
26. Bagheri-Yarmand, R., R.K. Vadlamudi, and R. Kumar, *Activating transcription factor 4 overexpression inhibits proliferation and differentiation of mammary epithelium resulting in impaired lactation and accelerated involution.* J Biol Chem, 2003. **278**(19): p. 17421-9.
27. Collin, G., et al., *Transcriptional repression of DNA repair genes is a hallmark and a cause of cellular senescence.* Cell Death Dis, 2018. **9**(3): p. 259.
28. Henson, J.D., et al., *DNA C-circles are specific and quantifiable markers of alternative-lengthening-of-telomeres activity.* Nat Biotechnol, 2009. **27**(12): p. 1181-5.
29. Henson, J.D., et al., *The C-Circle Assay for alternative-lengthening-of-telomeres activity.* Methods, 2017. **114**: p. 74-84.
30. O'Sullivan, R.J., et al., *Rapid induction of alternative lengthening of telomeres by depletion of the histone chaperone ASF1.* Nat Struct Mol Biol, 2014. **21**(2): p. 167-74.

REVIEWERS' COMMENTS

Reviewer #3 (Remarks to the Author):

I want to thank the reviewers for the extra effort in addressing the questions that were forthrightly and genuinely raised with the intent of improving the quality and message(s) of the paper.

Many aspects of the study remain unclear to me (and probably to the authors also). So, I wish them good luck with their further experiments.

Response to the reviewer's comments

Reviewer #3 (Remarks to the Author):

I want to thank the reviewers for the extra effort in addressing the questions that were forthrightly and genuinely raised with the intent of improving the quality and message(s) of the paper.

Many aspects of the study remain unclear to me (and probably to the authors also). So, I wish them good luck with their further experiments.

We thank the reviewer for the appreciation of the revision efforts of the manuscript and hope to address the remaining aspects in future studies.
